# Closing the Loop: Low‐Waste Phosphorus Functionalization Enabled by Simple Disulfides

**DOI:** 10.1002/cssc.202401895

**Published:** 2024-11-25

**Authors:** Thomas M. Horsley Downie, Ajdin Velić, Luis A. Coelho, Robert Wolf, Daniel J. Scott

**Affiliations:** ^1^ Institute of Inorganic Chemistry University of Regensburg 93040 Regensburg Germany; ^2^ Department of Chemistry University of Bath, Claverton Down Bath BA2 7AY UK

**Keywords:** Homogeneous catalysis, Oxidation, Phosphanes, Sulfur, White phosphorus

## Abstract

Useful monophosphorus products are obtained from both white and red phosphorus *via* a simple strategy involving initial oxidation by aryl disulfides followed by quenching with nucleophiles. Direct transformations of elemental phosphorus are usually very challenging, forcing chemists to instead rely on inefficient and hazardous multi‐step methods. However, here they are achieved using inexpensive and easy‐to‐handle reagents, providing access to diverse P−C, P−N and P−O bonded products in good yields. By isolating the thiolate byproducts of these reactions, a simple, closed loop can be achieved that produces only minimal, benign waste byproducts, in contrast to other direct methods. This closed loop can even be elaborated into a true (electro)catalytic cycle, which is extremely rare in the field of elemental phosphorus functionalization.

## Graphical abstract

Remarkably simple disulfides can be used to mediate the oxidative transformation of elemental phosphorus and diverse nucleophiles directly into useful P_1_ products. The reaction can be employed as part of a two‐step closed loop which produces minimal waste, and the key disulfide can even be employed in a fully catalytic manner.

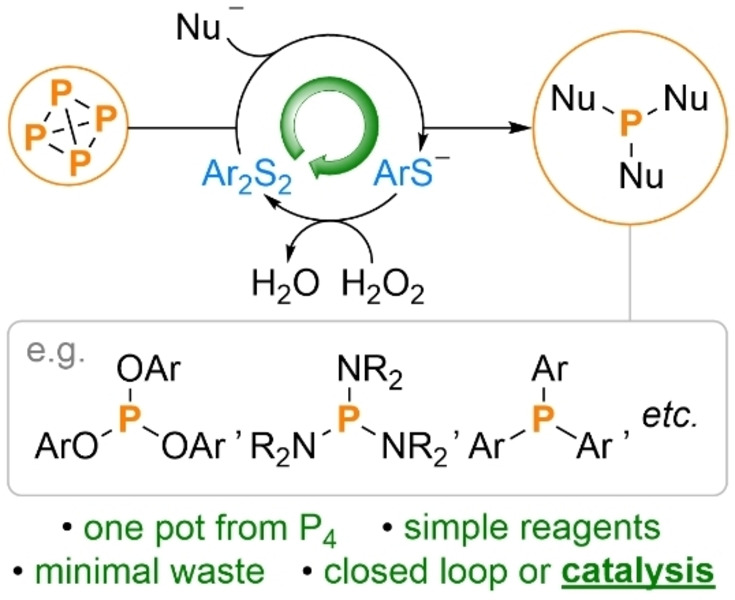

## Introduction

**Scheme 1:**
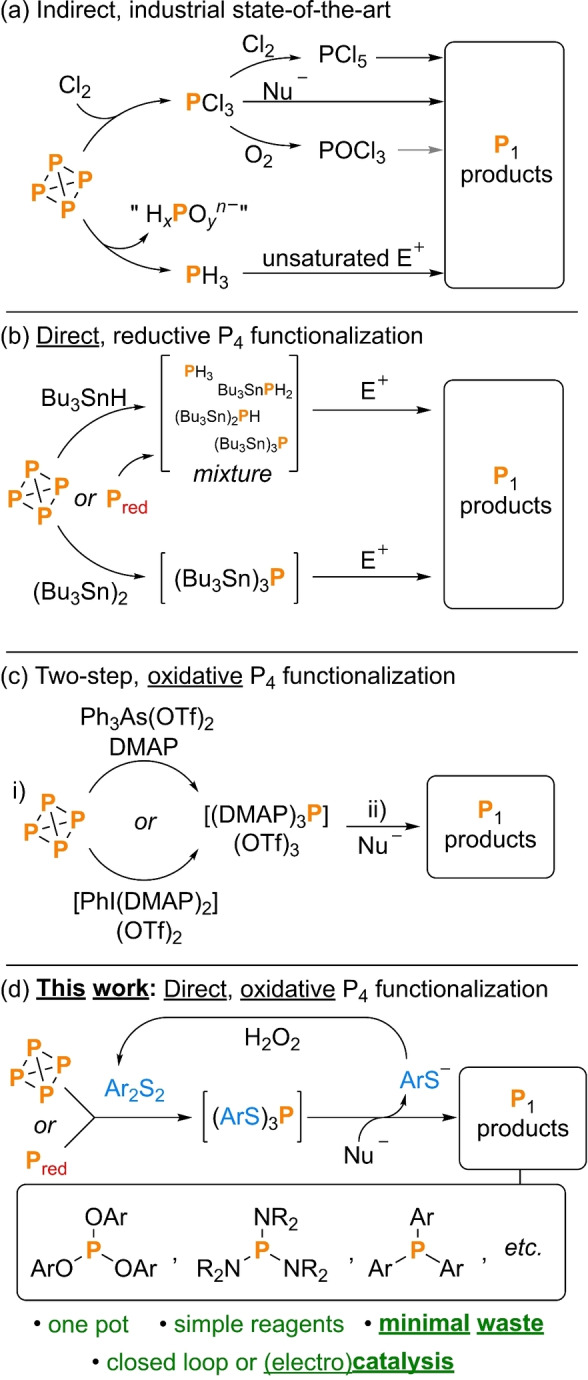
(a) Indirect industrial routes for the transformation of elemental phosphorus; (b) direct, reductive routes reported recently;[^47^, ^48^, ^49^, ^50^] (c) a two‐step oxidative route reported recently;^[51]^ and (d) direct, low‐waste oxidative functionalization, described herein. “H_*x*_PO_*y*_^*n−*^” indicates reaction‐specific oxidized byproducts such as H_3_PO_4_ and NaH_2_PO_2_. Nu^−^ and E^+^ indicate generic nucleophiles and electrophiles, respectively. DMAP=4–dimethylaminopyridine.

White phosphorus (P_4_) is the common starting point from which almost all commercially relevant monophosphorus compounds are prepared.[^1^, ^2^, ^3^] The combined importance of these P_1_ products both industrially and academically is hard to overstate, and the methods used to transform elemental P are thus exceedingly important. Probably the most widely used methods are the oxidation of P_4_ to PCl_3_ (which may be oxidized further to PCl_5_ or POCl_3_ in an additional step) and the disproportionation of P_4_ to form PH_3_ (alongside oxidized P_1_ species), as shown in Scheme 1a.[^4^, ^5^] These initial P_1_ intermediates, which have little independent value, can then be reacted in subsequent steps with nucleophiles (for PCl_3_, PCl_5_, POCl_3_) or unsaturated electrophiles (for PH_3_) to give simple, initial P_1_ products of interest.^[6]^

The obvious reliance of these routes on multiple reaction steps and extremely hazardous reagents and intermediates (e. g. Cl_2_, PH_3_) has long prompted calls for the development of alternatives,^[7]^ and particularly for direct routes that might achieve the same net transformations in a single reaction without the need for isolation or manipulation of potentially harmful intermediates.^[8]^ Efforts have also been made towards improving the synthesis of P_4_ itself,[^9^, ^10^, ^11^] or even bypassing P_4_ entirely.[^12^, ^13^, ^14^, ^15^, ^16^, ^17^] Although the fundamental reactivity of P_4_ has been investigated for decades,[^18^, ^19^, ^20^, ^21^, ^22^] it is only very recently that reports of direct, alternative reactions have begun to emerge in the chemical literature.[^23^, ^24^, ^25^] These reports provide encouraging proof that such transformations are possible. However, the number of examples to date remains extremely limited and those that have been described remain at very early stages of development, with each suffering from significant drawbacks related to expense, atom economy, hazards, and so on.[^26^, ^27^, ^28^, ^29^, ^30^, ^31^, ^32^, ^33^, ^34^, ^35^, ^36^, ^37^, ^38^, ^39^, ^40^, ^41^, ^42^] Of particularly general concern is waste production. Although current industrial methods are often criticized for producing theoretically unnecessary waste products (e. g. chloride salts), far *more* waste is produced by the direct methods that have been reported so far, much of which is also potentially extremely toxic (e. g. cyanides, organo‐Sn/As compounds).

There thus remains an acute need to develop new strategies in this area, particularly ones that can use simple, inexpensive reagents and produce only minimal and benign stoichiometric waste. A related goal is the development of efficient *catalytic* methods for the activation and transformation of P_4_. Catalysis is a cornerstone of modern green chemistry, but examples in the field of P_4_ chemistry remain exceedingly scarce, especially for the formation of P(III) products.[^43^, ^44^, ^45^, ^46^] A third goal is to develop methods that can activate not only P_4_ but also the much less reactive allotrope red phosphorus (P_red_). While P_red_ is prepared commercially *via* thermolysis of P_4_, and this extra step makes it less attractive as a precursor for industrial applications,^[2]^ use of P_red_ would be far preferable in most smaller scale laboratories, where safety concerns about the toxicity and pyrophoricity of P_4_ are not always easily surmounted. Similar safety concerns also surround many other simple P_1_ sources that might otherwise be ideal for laboratory use (e. g. PCl_3_, PH_3_; *vide supra*).

As part of our own contribution to the field of direct elemental P functionalization we have recently described methods by which P_4_ can first undergo reductive (hydro)stannylation to form either a mixture of P_1_ intermediates, (Bu_3_Sn)_*x*_PH_3‐*x*_ (*x*=0–3),[^47^, ^48^] or the single P_1_ intermediate (Bu_3_Sn)_3_P.^[49]^ Either option can then function as a simple “P^3−^” synthon that will react with electrophiles to furnish useful P_1_ products (Scheme 1b). Various products are accessible by these routes; however, the development of catalytic reactions based on this methodology has proven challenging, with only a single, very specific example achieved so far for a single substrate. Moreover, extending the observed reactivity from P_4_ to P_red_ was challenging, requiring significant changes to reaction conditions including significantly extended reaction times, more hazardous near UV irradiation, and a large excess of P_red_.^[50]^

More fundamentally, the scope of these reactions is inherently limited by the need for electrophilic substrates for the final functionalization step. For many target products a suitable electrophilic synthon may be unavailable, or impractical in comparison with an alternative, *nucleophilic* synthon. For example, aryl, amino and aryloxy motifs are hard to install using electrophilic reagents, but readily installable using common nucleophiles (e. g. ArMgX, R_2_NH, ROH).^[24]^ In this context, Weigand and co‐workers have recently reported an impressive and complementary procedure for transforming P_4_ into the “P^3+^” synthon (DMAP)_3_P^3+^ (DMAP=*N,N*‐dimethylaminopyridine), which can subsequently be derivatized with nucleophiles (Scheme 1c).^[51]^ However, so far this has only been achieved as two separate reaction steps, and it remains unclear whether the same net procedure could be achieved in a ‘one pot’ fashion.^[24]^ The procedure also requires relatively elaborate oxidants (e. g. Ph_3_As(OTf)_2_, PhI(OAc)_2_) in combination with a stoichiometric, coordinating base (e. g. DMAP), and while these can be regenerated by isolation of the reaction byproducts doing so involves relatively complex, multi‐step procedures.

With this in mind, we were motivated to investigate the development of an alternative strategy for oxidative phosphorus activation that would complement our previous reductive routes by allowing for subsequent functionalization using nucleophiles. Such a method would mimic conceptually the Cl_2_‐based routes currently employed by industry, achieving the same net transformations but with greater step economy and fewer hazardous reagents. We also wished to target from the outset a system whose design would be well suited to the development of catalytic reactivity and would ideally allow for functionalization of P_red_ as well as P_4_. Finally, we chose to target a system that would in principle produce only minor amounts of relatively benign stoichiometric waste. As noted above, stoichiometric waste formation is often highlighted as an undesirable aspect of the current industrial state‐of‐the‐art; however, in practice, the development of alternative, direct P_4_ transformation reactions has thus far always led to significant *decreases* in atom economy relative to these established methods.

From this starting point, we describe herein the development of a strategy for the atom‐efficient activation and subsequent functionalisation of elemental phosphorus. This method uses simple disulfides as mediators and can activate both P_4_ and P_red_ to furnish a wide variety of P_1_ products containing P−C, P−N, and P−O bonds. By recycling the thiolate byproducts these reactions can be made to produce only minimal and benign stoichiometric waste and can even be adapted to operate as part of a catalytic or electrocatalytic cycle, with excellent turnover numbers compared with other state‐of‐the‐art direct methods.

## Results and Discussion

The traditional synthesis of most P_1_ compounds employs a two‐step procedure. In the first step, elemental phosphorus is transformed into a “P^3+^” surrogate, such as PCl_3_. This intermediate is then converted into the desired products through reaction with suitable nucleophiles, often accompanied by the formation of salt waste. It is evident that such a two‐step strategy is challenging to convert into a catalytic protocol. We therefore sought an alternative strategy by which P_4_ could be cleanly oxidised using a single, simple reagent and directly transformed in ‘one pot’ with nucleophiles, Nu^−^, to give desired products Nu_3_P alongside a byproduct that could be directly and easily re‐oxidised to regenerate the starting oxidant. It was anticipated that these ambitious goals might be achievable using disulfides as the key oxidants (Scheme 1d). Wu reported in a brief communication almost 60 years ago that direct oxidation of P_4_ by disulfides R_2_S_2_ can be mediated by simple bases in polar solvents, resulting in the corresponding trithiophosphites (RS)_3_P.^[52]^ Follow‐up reports in the decades since have described modifications to the thiolation reaction, as well as related reactions for the synthesis of P(V) products, including recent notable work by the Tang group.[^53^, ^54^, ^55^, ^56^, ^57^, ^58^, ^59^, ^60^] However, surprisingly, the reactivity of isolated trithiophosphites themselves towards nucleophiles, as a strategy for P(III) product formation, does not appear to have attracted significant previous study. It was speculated that the good leaving group ability of simple thiolate moieties should allow trithiophosphites to act as convenient, electrophilic “P^3+^” synthons, forming desirable products upon addition of nucleophiles alongside liberation of “RS^−^” (as the thiol or a simple thiolate salt). Indeed, similar substitution of RS^−^ has been observed from or en route to a number of related P(V) species such as trithiophosphates, including in cases where these are derived from P_4_.[^35^, ^53^, ^55^, ^61^] Crucially, re‐oxidation of such “RS^−^” to R_2_S_2_ is well known and can be achieved using convenient, “green” oxidants such as H_2_O_2_.[^62^, ^63^] Recovery of “RS^−^” would thus furnish a simple, closed loop cycle requiring only inexpensive reagents and proceeding with good atom economy while producing only benign stoichiometric byproducts (e. g. H_2_O, simple halide salts). Moreover, because RS^−^ can be oxidised more easily than many other nucleophiles (e. g. RO^−^), incorporation of this step into a catalytic cycle should be feasible (*vide infra*). Gratifyingly, strong support for this mechanistic rationale was provided during this project′s early stages by Tang and coworkers, who reported a conceptually related system based on the diselenide Ph_2_Se_2_.^[46]^ However, it should be emphasised that this study was much more limited in scope (focusing exclusively on production of electron‐rich triarylphosphites from P_4_) and attempts to replace the diselenide with the more common disulfide motif led to very poor catalytic performance (*vide infra*).

### Stoichiometric Reaction Development

**Scheme 2:**
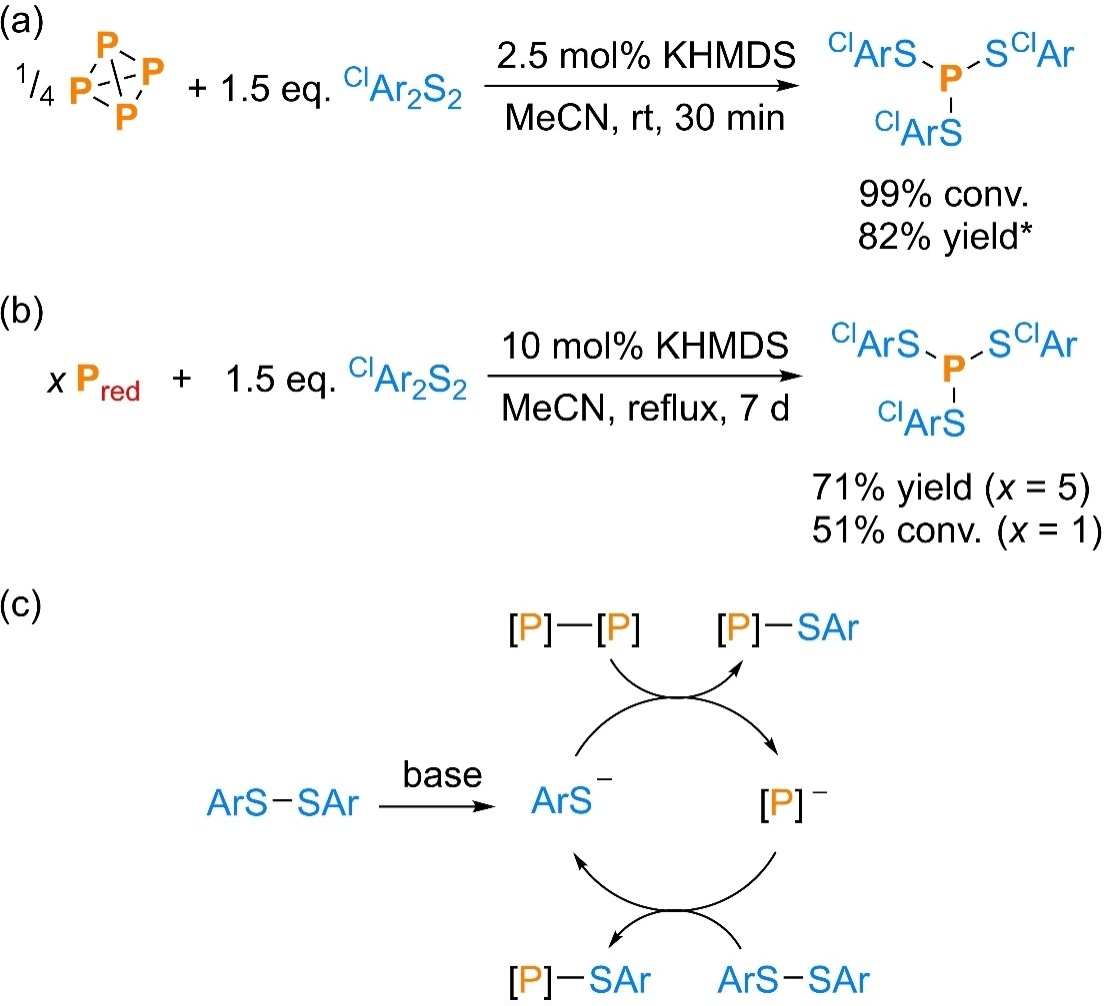
Base‐catalysed preparation of a trithiophosphite, (^Cl^ArS)_3_P, from the corresponding disulfide, ^Cl^Ar_2_S_2_, and (a) P_4_ or (b) P_red_; and (c) proposed mechanism.^[60]^ ^Cl^Ar=4‐chlorophenyl. [P]−[P] represents a generic P−P bond. “Yield” refers to isolated yield. Conversions (“conv.”) measured spectroscopically by quantitative ^31^P{^1^H} NMR spectroscopy. *Reaction stirred at rt for 18 h.

Our investigations began with a re‐validation of the P_4_ thiolation reaction described in Wu′s initial, brief report.^[52]^ Aryl disulfides Ar_2_S_2_ were chosen as the primary focus due to their easy electronic tunability (*vide infra*), combined with their relatively robust C−S bonds. Gratifyingly, clean conversion of P_4_ to (ArS)_3_P was observed upon addition of various Ar_2_S_2_ and catalytic quantities of simple bases in MeCN, fully consistent with the prior report. As a representative example, the 4‐chlorophenyl derivative (^Cl^ArS)_3_P could be formed essentially quantitatively in as little as 30 min at room temperature (rt; see Figure S5) and the product could easily be isolated at preparative scale in excellent yield (82 %; Scheme 2a). Similar reactivity was also observed in THF.

Satisfyingly, while Wu focused exclusively on P_4_, initial experiments also quickly confirmed the ability of the same method to transform P_red_ (Scheme 2b). While longer reaction times and refluxing were needed to achieve good conversion, little other alteration of the conditions used to transform P_4_ was required. Thus, (^Cl^ArS)_3_P was isolated cleanly and in good yield (71 %) from a reaction using 5 eq. P_red_. Moreover, good spectroscopic conversion (51 %) could be achieved even without excess P_red_, in marked contrast to reductive routes using Bu_3_SnH (*vide supra*).^[50]^ We assume that this P_red_ thiolation proceeds *via* an analogous mechanism to that proposed previously for P_4_, in which each P−P bond is cleaved by attack of ArS^−^, followed by attack of the resulting “P^−^” moiety on an ArS−SAr bond (Scheme 2c).^[60]^ As an aside, we note that this is an example of elemental phosphorus being broken down “*one P−P bond at a time*”, which we have recently argued is highly beneficial for P_4_ functionalization processes.^[47]^

**Scheme 3:**
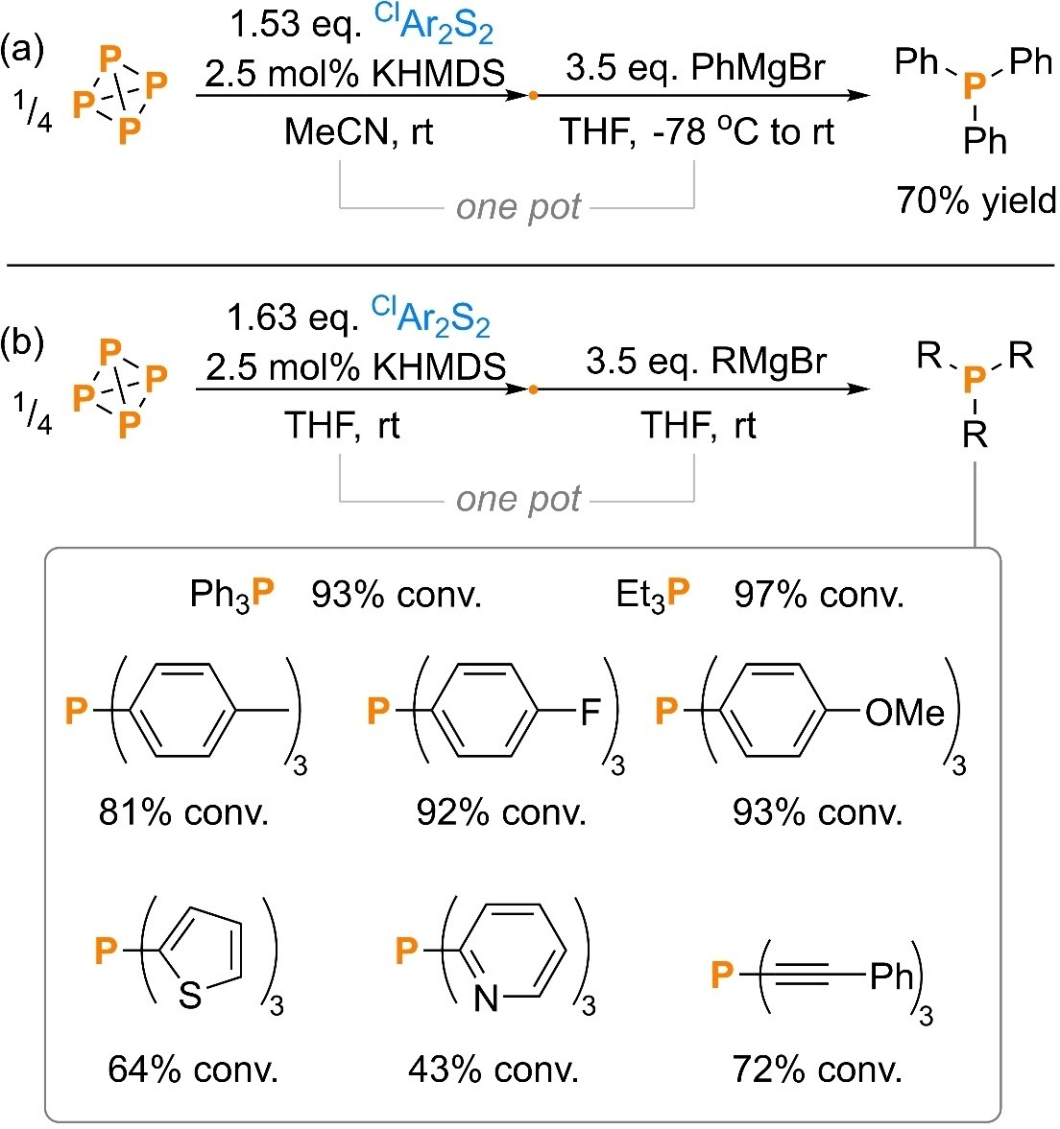
(a) Synthesis and isolation of Ph_3_P and (b) synthesis and quantification of other R_3_P directly from P_4_. “Yield” refers to isolated yield. Conversions (“conv.”) measured spectroscopically by quantitative ^31^P{^1^H} NMR spectroscopy. ^Cl^Ar=4‐chlorophenyl.

Since the viability of simple trithiophosphites as electrophilic P_1_ synthons for P(III) products does not seem to have been studied to any significant extent, we continued our investigations by first establishing the elementary reactivity of (^Cl^ArS)_3_P towards common organometallic nucleophiles. Ph_3_P is an extremely important industrial compound, finding use both as a ligand for transition metal catalysts and as a reagent for Wittig chemistry (e. g. for vitamin A synthesis),^[4]^ and is therefore often invoked as an ‘archetypal’ P_1_ product whose synthesis directly from P_4_ would be highly desirable.^[24]^ Thus, it was very satisfying to observe that addition of a slight excess of PhMgBr (3.5 eq. per P atom) to a solution of *in situ* generated (^Cl^ArS)_3_P resulted in excellent spectroscopic conversion to the desired product, which at larger scale could be isolated from the crude reaction mixture by simple sublimation in a very good 70 % yield (Scheme 3a and b). To our knowledge this is only the second example of Ph_3_P being prepared in a synthetically useful yield from P_4_ in a single reaction, with the first requiring a far more elaborate Ti^III^ reagent as mediator (a two‐step synthesis is also known, as are one‐step syntheses of more bulky Ar_3_P).[^41^, ^43^, ^44^, ^45^, ^51^]

Gratifyingly, we observed similar ‘one pot’ results when PhMgBr was replaced with other Grignard reagents. Thus, a variety of tertiary phosphine products could be generated with good to excellent conversion (up to 97 % spectroscopic yield), including electron rich and electron poor aryl, heteroaryl, and alkyl derivatives (Scheme 3b). Similar product formation was also observed using more electron‐rich disulfides, such as the parent Ph_2_S_2_, in place of ^Cl^Ar_2_S_2_. However, this generally led to reduced selectivity and correspondingly poorer spectroscopic yields. This is tentatively attributed to the improved leaving group ability of the ^Cl^ArS^−^ moieties resulting in better selectivity for the desired substitution reaction at P over competing, unproductive attack at S (see SI, section 3.2.2 for additional discussion).

**Scheme 4:**
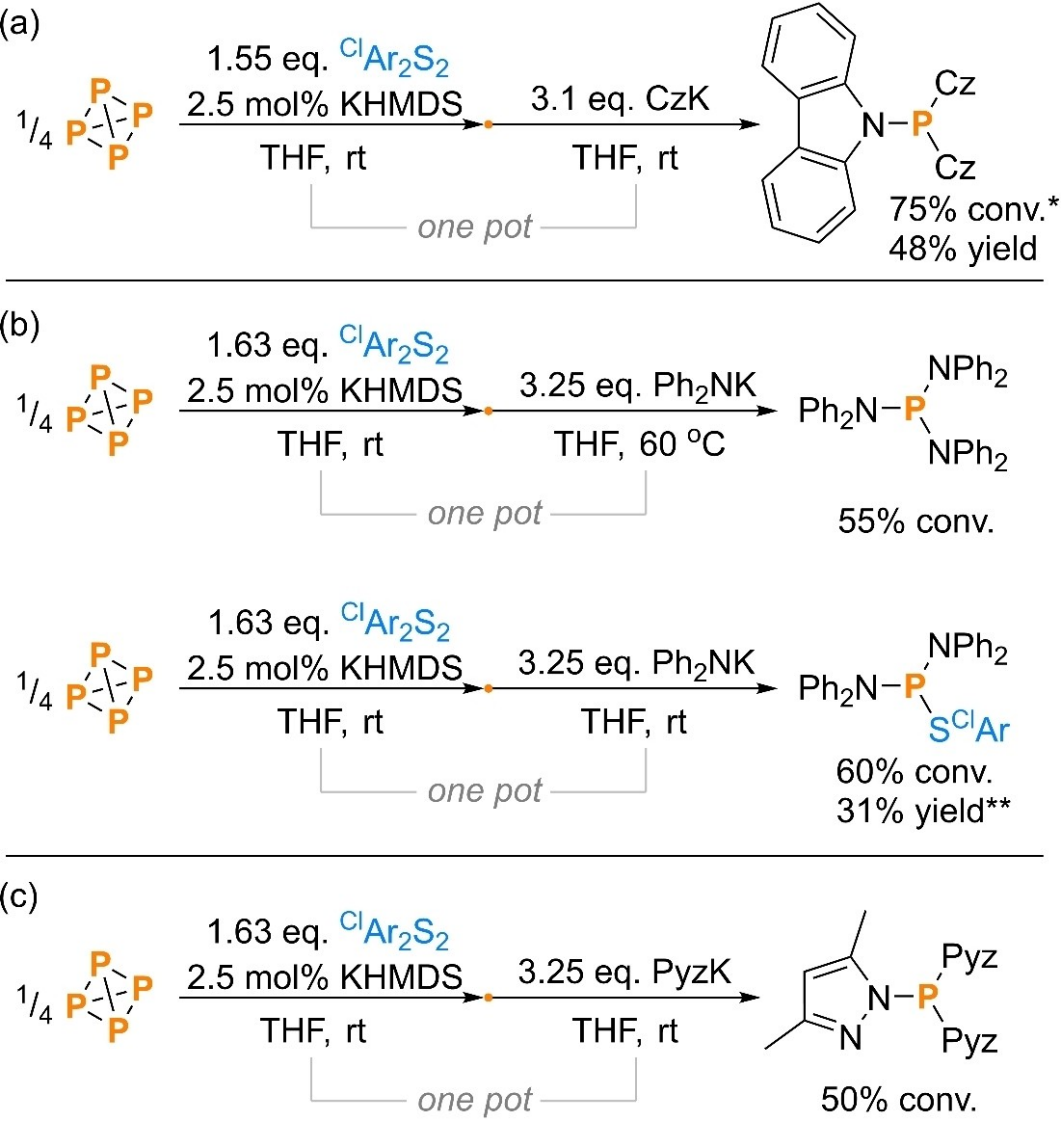
(a) Synthesis and isolation of Cz_3_P; (b) synthesis and quantification of (Ph_2_N)‐substituted P_1_ products; and (c) synthesis and quantification of Pyz_3_P directly from P_4_. “Yield” refers to isolated yield. Conversions (“conv.”) measured spectroscopically by quantitative ^31^P{^1^H} NMR spectroscopy. ^Cl^Ar=4‐chlorophenyl. *Reaction using 1.63 eq. ^Cl^Ar_2_S_2_, 3.25 eq. CzK, CzK added at 0 °C and warmed to rt. **Reaction using 2.0 eq. Ph_2_NK.

To generalize the observed reactivity still further, we also investigated the use of *N*‐centered nucleophiles. Thus, through adaptation of the previous procedure using potassium carbazolide (CzK) in place of the organometallic reagent, the formation of the target product Cz_3_P was observed with good spectroscopic conversion (75 %), and after scaleup was isolated in a synthetically useful yield (48 %; Scheme 4a). These P−N bond forming reactions were found to be less predictable than the previous P−C bond formations. For example, attempted reactions using the aliphatic amides potassium diethylamide and potassium pyrrolidide or the primary aromatic amides Ph(H)NM (M = Li or K) gave unsatisfactory results.^[64]^ Meanwhile, replacement of the carbazolyl nucleophile with the very similar diphenylamido potassium led to formation of the expected product (Ph_2_N)_3_P only after heating to 60 °C (see SI, section 3.2.2, and Figure S30). At rt an alternative product was formed selectively and with good spectroscopic conversion (60 %), which was assigned based on its ^31^P NMR shift as the partially substituted product (Ph_2_N)_2_PS^Cl^Ar (Scheme 4b).^[65]^ Upon repeating this reaction with a more proper stoichiometry, (Ph_2_N)_2_PS^Cl^Ar was readily isolated in modest yield (31 %) as colorless crystals, permitting the verification of its structure by X‐ray crystallography (Figure S75). In contrast, a further reaction using potassium 3,5‐dimethylpyrazolide, PyzK, as the nucleophile more reliably mimicked the initial reactivity of CzK, leading to formation of the fully substituted product, Pyz_3_P, with reasonable spectroscopic conversion (50 %; Scheme 4c). The reason for this more temperamental reactivity with *N*‐centred than with *C*‐centred nucleophiles has not been investigated in detail. However, we speculate that it may be due to the reduced reactivity of the former allowing subtle differences in aggregation to have a greater impact on reactivity. If so, this suggests the potential for further improvements through more extensive reaction optimization in the future.

**Scheme 5:**
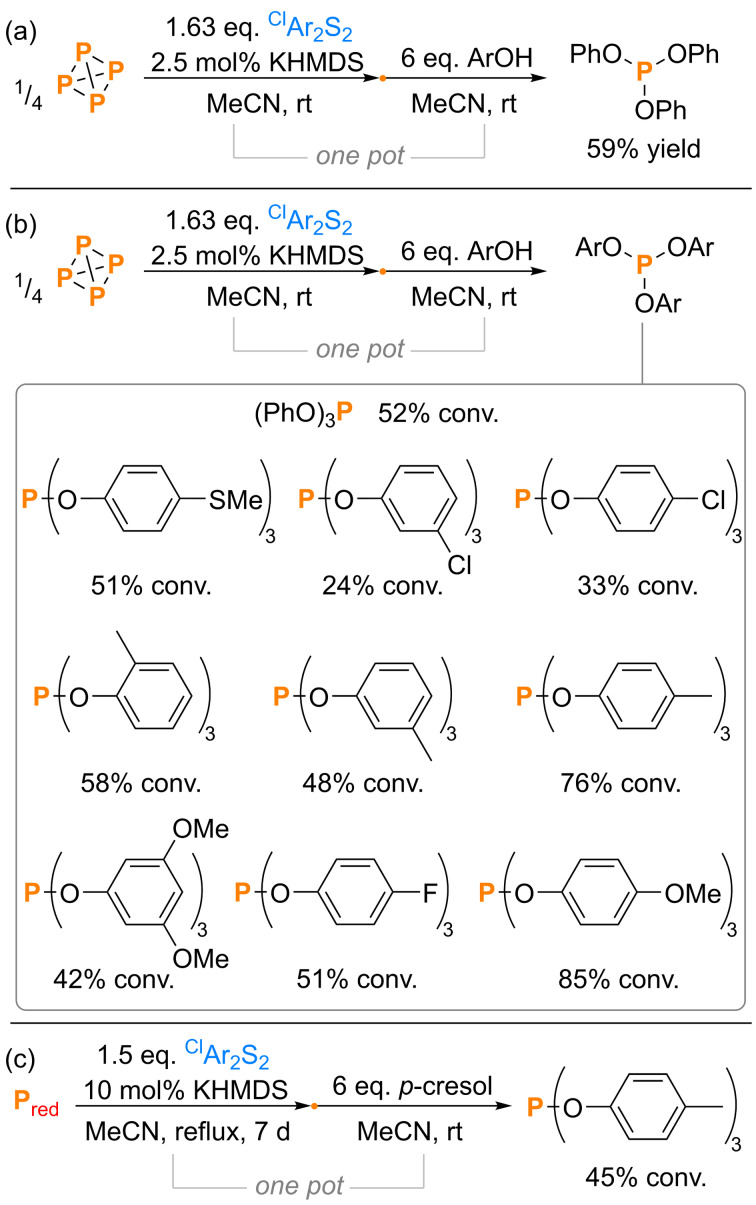
(a) Synthesis and isolation of (PhO)_3_P directly from P_4_;^[66]^ (b) synthesis and quantification of other (ArO)_3_P directly from P_4_; and (c) synthesis and quantification of (ArO)_3_P directly from P_red_. “Yield” refers to isolated yield. Conversions (“conv.”) measured spectroscopically by quantitative ^31^P{^1^H} NMR spectroscopy. ^Cl^Ar=4‐chlorophenyl.

Having investigated *C*‐ and *N*‐centered nucleophiles, we explored *O*‐centered nucleophiles as a final substrate class. Again, without any further optimization, PhOH was added as a simple model substrate to *in situ* generated (^Cl^ArS)_3_P, resulting in direct formation of (PhO)_3_P, which was isolated cleanly and in good yield at preparative scale *via* distillation (59 %; Scheme 5a).^[66]^ NMR‐scale experiments confirmed similar (ArO)_3_P formation for a variety of other substituted phenol derivates with spectroscopic conversions up to 85 % and with the best results generally being observed for more electron rich substrates (Scheme 5b), consistent with their higher nucleophilicity. As a proof of concept, formation of (PhO)_3_P starting from P_red_ was also achieved with reasonable spectroscopic conversion, even using P_red_ as the limiting reagent (45 %, Scheme 5c; this compares very well with the 51 % conversion to (^Cl^ArS)_3_P shown in Scheme 2b). Because no excess of P_red_ is required, this reaction already significantly outperforms our previous efforts on reductive P_red_ functionalization in terms of P atom economy.^[50]^

### Thiol Recovery and Closed Loop Synthesis

**Scheme 6:**
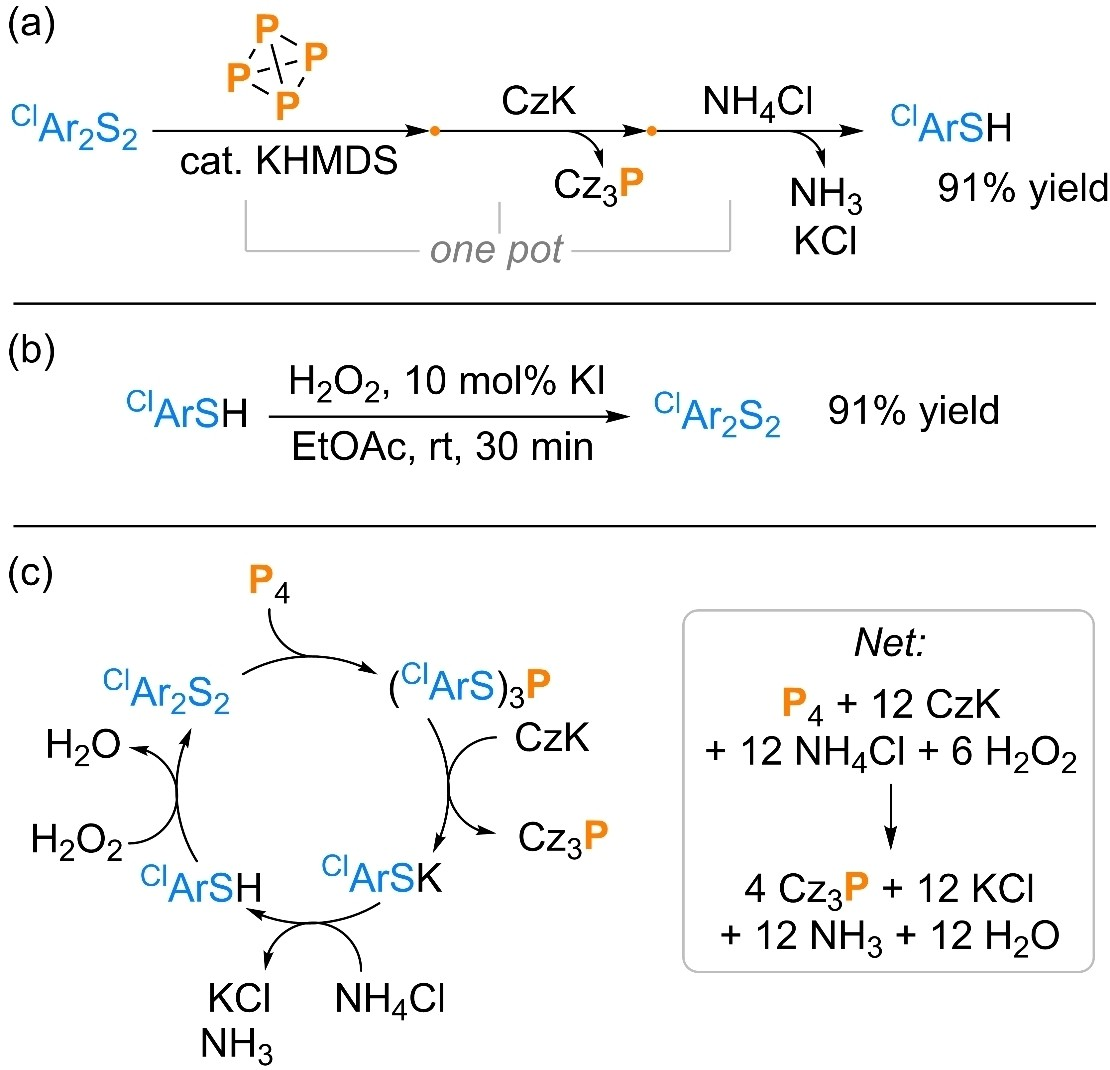
Low waste transformation of P_4_ into Cz_3_P: (a) synthesis and isolation of Cz_3_P and recovery of ^Cl^ArSH; (b) green oxidation of ^Cl^ArSH to ^Cl^Ar_2_S_2_; and (c) net ‘closed’ synthetic loop. “Yield” refers to isolated yield. ^Cl^Ar=4‐chlorophenyl.

Having established both the ability of trithiophosphites to act as versatile electrophilic precursors to useful P_1_ compounds and the viability of telescoping their generation and consumption into a single, ‘one pot’ reaction, we turned our attention to closing the synthetic cycle illustrated in Scheme 1d, by recovering and recycling the corresponding thiolate byproducts. The synthesis of Cz_3_P was chosen as a representative example and, gratifyingly, it was confirmed that during workup the thiolate byproduct could easily be recovered as the corresponding thiol after quenching with NH_4_Cl, in excellent isolated yield (91 %, Scheme 6a).

It is well known that aryl thiols can be easily re‐oxidized to the corresponding disulfides, with high yields achievable using extremely cheap, “green” terminal oxidants such as O_2_ and H_2_O_2_.[^62^, ^63^] As a confirmation, in our hands, the isolated ^Cl^ArSH could be preparatively re‐oxidized to ^Cl^Ar_2_S_2_ in an excellent 91 % isolated yield using H_2_O_2_ and catalytic KI (Scheme 6b). Combining this step with the reactions demonstrated previously provides the closed loop shown in Scheme 6c. Crucially, the combined cycle produces H_2_O, NH_3_ (from NH_4_Cl) and the simple, benign metal salt KCl as the sole stoichiometric byproducts. Thus, in principle, this method allows direct P_4_ functionalization to be achieved while producing only minimal and relatively harmless net waste, competitive with the current industrial state of the art.^[4]^

### Catalytic Reaction Development

With stoichiometric demonstration of the proposed synthetic cycle having been achieved, we shifted our attention to the development of a truly catalytic cycle, as effective examples of catalytic P_4_ transformation remain stubbornly rare. The synthesis of triarylphosphites was chosen as a model catalytic reaction, since phenoxide moieties should be significantly harder to oxidize than the catalytic thiolate, ArS^−^, allowing compatibility between the nucleophile and terminal oxidant. Moreover, triarylphosphites are versatile reagents, ligands and additives and have broad applications both industrially and in academia.^[4]^ However, to our knowledge Tang′s diselenide system noted above is the sole example of catalytic conversion of P_4_ into phosphite esters that has been reported previously,^[46]^ and this required high loadings of the diselenide catalyst alongside a significant excess of the phenol, while being limited to relatively electron‐rich substrates.

**Scheme 7:**
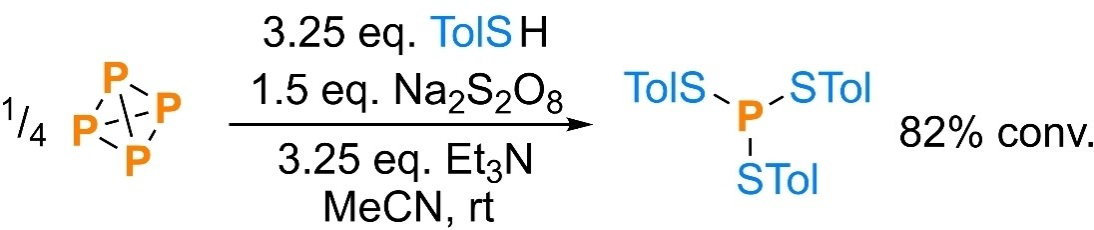
Synthesis of a trithiophosphite directly from P_4_ and the corresponding thiol. Tol=4–methylphenyl. Conversion (“conv.”) measured spectroscopically by quantitative ^31^P{^1^H} NMR spectroscopy.

To provide an initial, ‘stoichiometric’ proof‐of‐concept and identify an appropriate terminal oxidant, P_4_ was combined with a simple, model thiol TolSH (Tol=4‐methylphenyl) and various oxidizing reagents in the presence of Et_3_N as a base. The most effective proved to be inexpensive sodium persulfate, Na_2_S_2_O_8_, which was employed to give clean conversion to (TolS)_3_P, consistent with the desired thiol oxidation and consecutive P_4_ thiolation (Scheme 7).^[67]^ Only trace P_4_ consumption was observed in the absence of an oxidant. When the reaction was repeated in the presence of PhOH an almost identical outcome was observed, differing only by the formation of a small amount of (PhO)_3_P alongside (TolS)_3_P, confirming that the desired oxidation step is compatible with the presence of this model nucleophile (see SI, section 4.1.2).

**Scheme 8:**
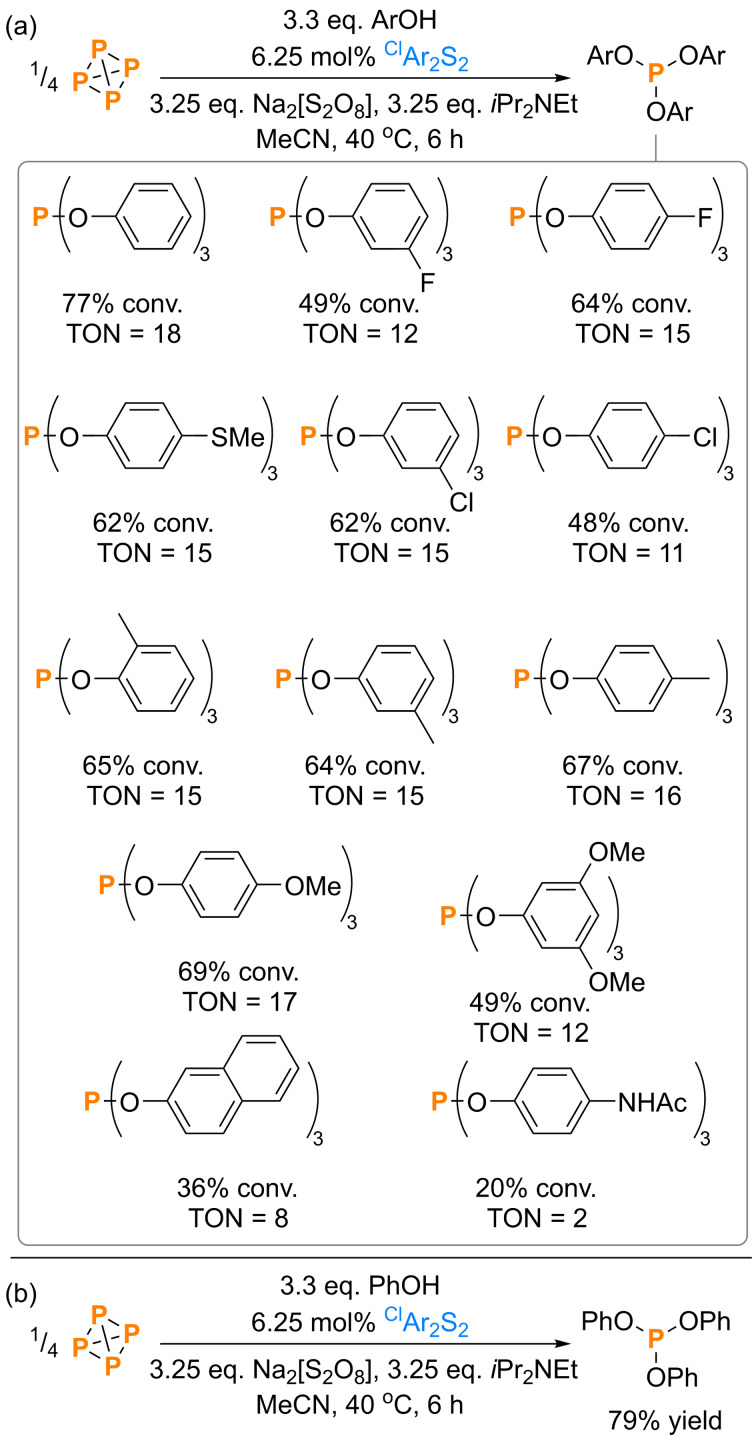
(a) Catalytic synthesis and quantification of triarylphosphites and (b) catalytic synthesis and isolation of (PhO)_3_P directly from P_4_.^[66]^ “Yield” refers to isolated yield. Conversions (“conv.”) measured spectroscopically by quantitative ^31^P{^1^H} NMR spectroscopy. ^Cl^Ar=4‐chlorophenyl.

With the mutual compatibility of the necessary reagents and reaction steps thus illustrated, we finally pursued genuine catalysis. Following optimization of the disulfide catalyst, auxiliary base, and reaction conditions the model substrate (PhO)_3_P could be generated in a very good 77 % spectroscopic conversion after just 6 h at 40 °C, using just 6.25 mol % ^Cl^Ar_2_S_2_ per P atom, and without a significant excess of substrate or base, although a *ca*. two‐fold excess of the terminal oxidant was employed (Scheme 8a; for comparison, Tang and coworkers use 25 mol % Ph_2_Se_2_ and K_3_PO_4_, a two‐fold excess of ArOH, and DMSO co‐solvent as the terminal oxidant).^[46]^ This result equates to a turnover number (TON) for the disulfide catalyst of approximately 18 (TON is defined here with respect to the number of P−P bonds cleaved, consistent with other recent reports).^[48]^ This compares very well with the handful of other known examples of catalytic P_4_ chemistry (for example: from our own recent contributions, TON≈11,^[48]^ TON≈10;^[49]^ from Tang and coworkers,^[46]^ maximum TON<6 with Ph_2_Se_2_, <3 with disulfides).

The same conditions were used without any further modifications to prepare a variety of other triarylphosphites, including both sterically hindered and unhindered examples, and products containing both electron‐donating and electron‐withdrawing groups (*cf*. electron‐rich substrates only in ref. ^46^). Almost all achieved similar spectroscopic conversions (*ca*. 50–70 %) and TONs. Unlike the stoichiometric regime there is no obvious correlation between conversions and substrate electronics (*cf*. Scheme 5) and notably, for many substrates even higher conversions were observed than in the equivalent stoichiometric reactions. As a proof of principle, the synthesis of the model product (PhO)_3_P was also performed at preparative scale, which allowed isolation of the product in very good yield (79 %, Scheme 8b).^[66]^

**Scheme 9:**
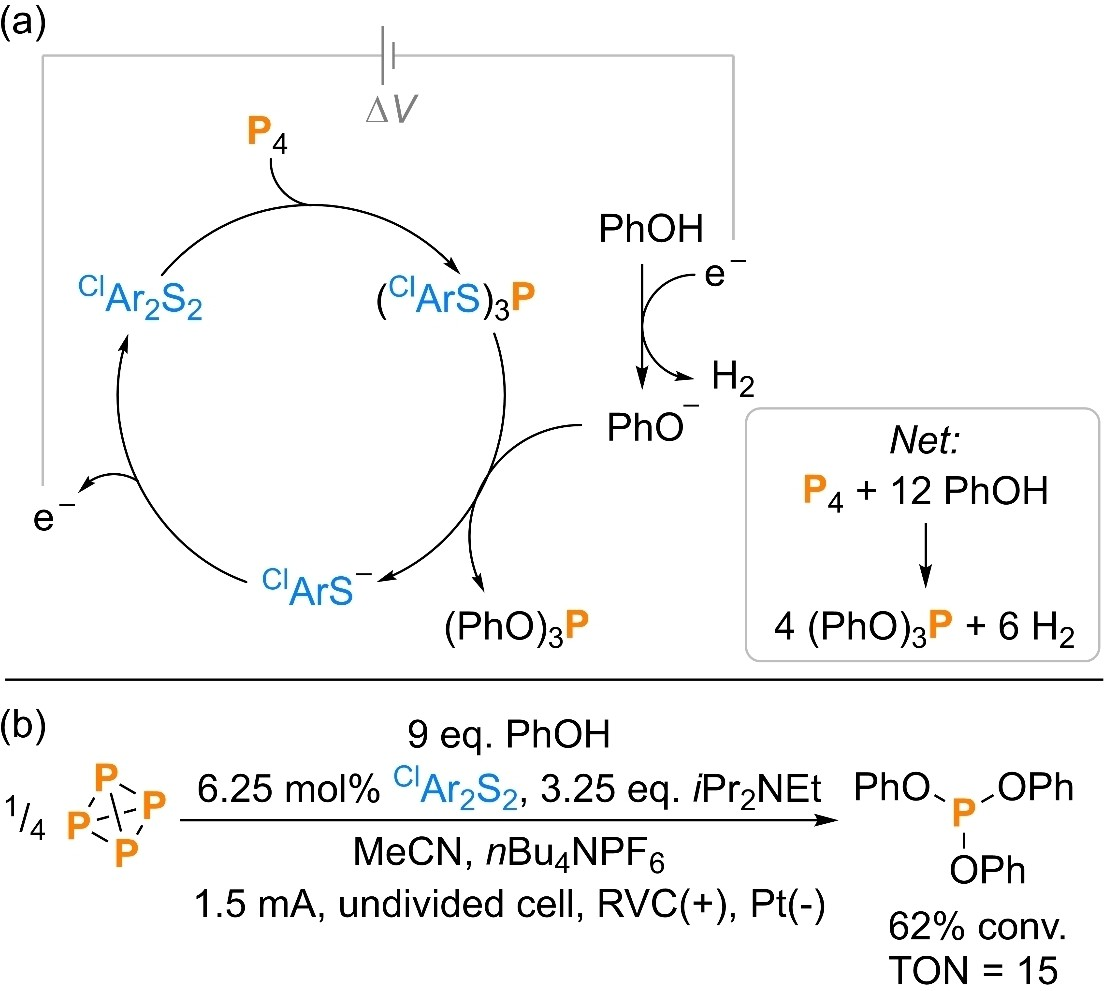
(a) Proposed catalytic cycle and (b) experimental proof of concept for electrocatalytic synthesis of (PhO)_3_P directly from P_4_. Conversion (“conv.”) measured spectroscopically by quantitative ^31^P{^1^H} NMR spectroscopy. ^Cl^Ar=4‐chlorophenyl.

Finally, we also pursued the *electro*catalytic functionalization of P_4_.^[68]^ While the specific oxidants used in this study are benign, the use of a chemical oxidant still inevitably contributes to waste formation (e. g. sulfate salts for the catalytic reactions shown in Scheme 8). This could be mitigated further by using electrochemical methods of oxidation. As a model, it was anticipated that oxidative P_4_ functionalization at the anode might be combined with hydrogen evolution (HER) from phenol at the cathode to generate the active phenoxide nucleophile, formally producing H_2_ as the sole stoichiometric byproduct (Scheme 9a).

As a proof of concept, the previously optimized conditions for the catalytic synthesis of (PhO)_3_P were translated directly into an electrochemical procedure, with only very minor experimental alterations (see SI section 5.1). Even without any further optimization, controlled current electrolysis in an undivided cell with *n*Bu_4_NPF_6_ electrolyte, RVC working electrode and Pt counter‐electrode led to formation of the target product (PhO)_3_P in 62 % spectroscopic yield (Scheme 9b). This corresponds to a TON of 15, which is already comparable to the optimized non‐electrochemical procedure (*cf*. Scheme 8a).

## Conclusions

We have described herein the development of an oxidative method for the direct transformation of P_4_ into a variety of different P_1_ products containing P−C, P−N and P−O bonds. Direct reactions of this type are extremely rare, and compared to the few existing examples this strategy is both highly versatile and unusually atom economical. It can be optimized to produce only minimal and benign stoichiometric waste, either through simple ‘closed loop’ strategies or true catalysis, and it is practically simple, requiring only very cheap, commercially available reagents. Indeed, one of the most striking features of this new method is the remarkable simplicity of the key Ar_2_S_2_ redox mediator/catalyst, whose successful use clearly emphasises that functionalization of elemental phosphorus (even *catalytic* functionalization, which is usually considered especially challenging) need not necessarily require elaborate reagents or additives.

Alongside these primary studies we have also provided clear, successful proofs of concept for two even more ambitious goals: *electrocatalytic* P_4_ functionalization, and the functionalization of P_red_, all using the same, simple Ar_2_S_2_‐based strategy. Studies to further optimize and generalize both of these methods are ongoing in our laboratories and will be reported in due course.

## Author Contributions

DJS performed initial studies and developed proof‐of‐concept stoichiometric and catalytic procedures. TMHD optimized the stoichiometric reactions. AV optimized the catalytic reactions. LAC developed the electrochemical reaction and scale‐up of the catalytic reaction. DJS and RW conceived, oversaw, and directed the project. DJS prepared the manuscript, with input from all authors.

## Conflict of Interests

A patent relating to the work described herein has been filed by DJS, RW and the University of Regensburg (EP 23 152 062.8). The authors declare no other conflicts of interest.

1

## Supporting information

As a service to our authors and readers, this journal provides supporting information supplied by the authors. Such materials are peer reviewed and may be re‐organized for online delivery, but are not copy‐edited or typeset. Technical support issues arising from supporting information (other than missing files) should be addressed to the authors.

Supporting Information

## Data Availability

The data that support the findings of this study are available in the supplementary material of this article.
