# Supplementary material for: Closing the Loop: Low‐Waste Phosphorus Functionalization Enabled by Simple Disulfides
Source: ChemSusChem. 2024 Nov 25;18(7):e202401895. doi: 10.1002/cssc.202401895 (PMC11960583; doi:10.1002/cssc.202401895)
Supplement: Supplementary file 1 — Supporting Information [file CSSC-18-e202401895-s001.pdf]

ChemSusChem

Supporting Information

Closing the Loop: Low-Waste Phosphorus Functionalization Enabled by Simple Disulfides

Thomas M. Horsley Downie, Ajdin Velić, Luis A. Coelho, Robert Wolf,* and Daniel J. Scott*

Supporting Information

Closing the Loop: Low-Waste Phosphorus Functionalisation Enabled by Simple Disulfides

Thomas M. Horsley Downie,[a] Ajdin Velić,[a] Luis A. Coelho,[a,b] Robert Wolf,*[a] Daniel J. Scott*[b]

[a] Institute of Inorganic Chemistry, University of Regensburg, 93040 Regensburg, Germany

[b] Department of Chemistry, University of Bath, Claverton Down, Bath, BA2 7AY, United Kingdom

E-mail: robert.wolf@ur.de, ds2630@bath.ac.uk

Table of contents

1. General experimental methods **S4**

2. Synthesis of starting materials **S7**

2.1. Synthesis of *bis*(4-chlorophenyl) disulfide S7

3. Stoichiometric reaction development **S9**

3.1. Generation of $(ArS)_3P$ from P_4 and Ar_2S_2 **S9**

3.1.1. Transformation and *in situ* measurement of conversion S9

3.1.2. Synthesis and isolation of $(ArS)_3P$ S10

3.2. One pot synthesis of tertiary phosphines from P_4 *via* $(ArS)_3P$ **S13**

3.2.1. Synthesis and isolation of Ph_3P S13

3.2.2. Discussion of differences in reaction outcome using different Ar_2S_2 S16

3.2.3. Generation of Ar_3P and *in situ* measurement of conversion S17

3.3. One pot synthesis of P–N bonded P_1 products from P_4 *via* $(ArS)_3P$ **S26**

3.3.1. Synthesis and isolation of tris(*N*-carbazolyl)phosphine S26

3.3.2. Generation of P–N bonded P_1 products and *in situ* measurement of conversion S30

3.3.3. Synthesis and isolation of $(Ph_2N)_2PS^{Cl}Ar$ S34

3.4. One pot synthesis of phosphite esters *via* $(ArS)_3P$ **S37**

3.4.1. Synthesis and isolation of triphenylphosphite S37

3.4.2. Generation of phosphite esters from P_4 and *in situ* measurement of conversion S39

3.4.3. Generation of phosphite ester from P_{red} and *in situ* measurement of conversion S49

4. Catalytic reaction development **S51**

4.1. Initial reaction development **S51**

4.1.1. Generation of $(TolS)_3P$ from P_4 and TolSH S51

4.1.2. Generation of $(TolS)_3P$ from P_4 and TolSH in the presence of PhOH S53

4.1.3. Optimisation of the catalytic synthesis of $(PhO)_3P$ from P_4 and PhOH S55

4.2. Catalytic synthesis of $(ArO)_3P$ from P_4 and ArOH **S62**

4.2.1. NMR scale procedure for *in situ* measurement of conversion S62

4.2.2. Larger scale catalytic synthesis of $(PhO)_3P$ S76

5. Electrocatalytic reaction development **S78**

5.1. Proof-of-concept experiment **S78**

5.1.1 Electrocatalytic generation of $(PhO)_3P$ and *in situ* measurement of conversion S78

6. XRD data for $(Ph_2N)_2PS^{Cl}Ar$ **S82**

7. References for supporting information **S84**

1. General experimental methods

Unless stated otherwise, all reactions and manipulations were performed under an N_2 atmosphere (< 0.1 ppm O_2, H_2O) through use of MBraun Unilab and GS MEGA Line gloveboxes, and standard Schlenk line techniques. All glassware was oven-dried (160 °C) overnight prior to use.

PhMe and THF were purified using an MBraun SPS-800 system and stored over molecular sieves (3 Å). DME, 1,4-dioxane and PhH were distilled from Na metal in the presence of benzophenone and stored over molecular sieves (3 Å). MeCN was distilled from CaH_2 and stored over molecular sieves (3 Å). DMSO was purchased dry and additionally dried over three sequential batches of molecular sieves (3 Å). DMF was dried over molecular sieves (3 Å).

C_6D_6 was distilled from K metal and stored over molecular sieves (3 Å).

P_4 was sublimed prior to use. Aryl disulfides Ar_2S_2 were either purchased from major chemical suppliers or prepared in accordance with the literature (see section 2.1). All other materials were purchased from major suppliers. Unless noted otherwise, solids were dried under vacuum and liquids were degassed and dried over molecular sieves (3 Å) unless already supplied under inert atmosphere.

Unless noted otherwise, NMR spectra were recorded at room temperature on Bruker Avance 400 (400 MHz) spectrometers and were processed using Topspin 3.2. Chemical shifts, δ, are reported in parts per million (ppm); ^1H NMR and ^{13}C NMR shifts are reported relative to $SiMe_4$ and were referenced internally to residual solvent peaks, while ^{31}P NMR shifts were referenced externally to 85 % H_3PO_4 (aq.). The abbreviations s, d, t, q, m are used to indicate singlets, doublets, triplets, quartets and multiplets, respectively. Except where indicated otherwise, integrals for $^{31}P\{^1H\}$ and ^{31}P spectra are provided for the purposes of qualitative comparison only, and should not be considered quantitatively accurate.

Reactions conducted on NMR scale for which spectroscopic conversions/yields of a product are reported were analyzed by $^{31}P\{^1H\}$ spectra using only a single scan (DS = 0, D1 = 2 s), as we have previously reported.[45] Since $(ArO)_3P$ products feature prominently in this report, this methodology has been re-validated for this compound class. The quantitative accuracy of this method was confirmed by preparing solutions of $(PhO)_3P$ and 0.05 mmol Ph_3PO (as a stock solution in MeCN, 0.08M, 625 μL) and comparing the measured and expected relative integrations. Excellent agreement was observed, as shown below in Figure S1 and Table S1.

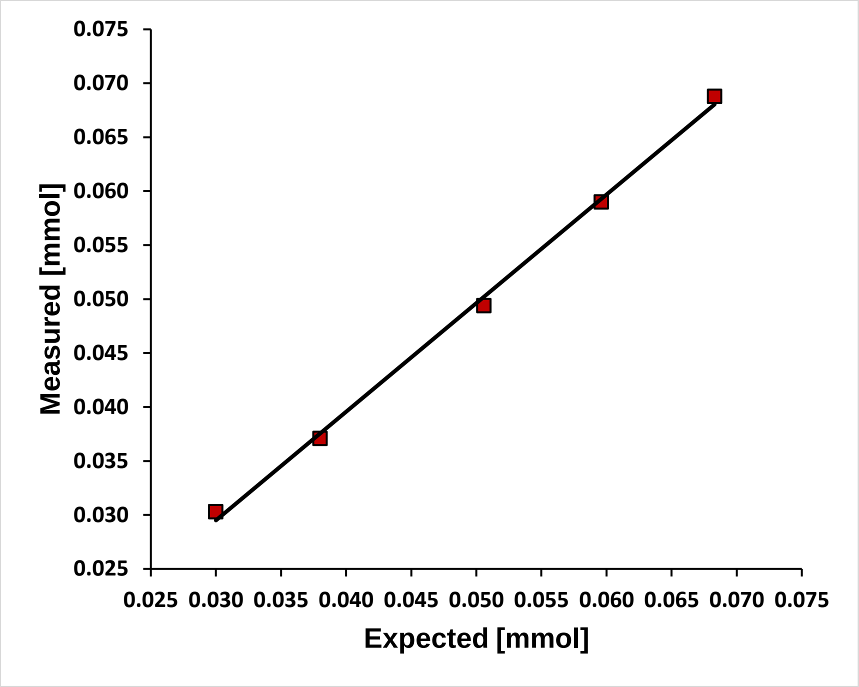

Figure S1. A plot showing the consistency between measured (by integration against 0.05 mmol Ph_3PO using a $^{31}P\{^1H\}$ experiment (zgpg) with a single scan) and expected (based on mass added) molar quantities of $P(OPh)_3$ in MeCN (625 μL) solutions.

Table S1. Masses and molar quantities of $(PhO)_3P$ used in solutions for validation of the quantitative $^{31}P\{^1H\}$ NMR measurements, with molar quantites measured by integration against an 0.05 mmol Ph_3PO.

Mass $P(OPh)_3$	Expected mmol	Measured mmol
9.3 mg	0.0300	0.0303
11.8 mg	0.0380	0.0371
15.7 mg	0.0506	0.0494
18.5 mg	0.0596	0.0590
21.2 mg	0.0683	0.0688

Electrochemical reactions were performed using tetrabutylammonium hexafluorophosphate supporting electrolyte ($^{n}Bu_4NPF_6$), which was dried under vacuum at 120 °C for 24 h prior to use. Reactions were conducted in two-electrode cells with an IKA ElectraSyn 2.0 potentiostat/galvanostat, using RVC foam (6.35 x 150 x 150 mm, 96.5% porosity, 24 pores/cm, purchased from GoodFellow GmbH, product code: VC00-FA-000125) as anode material and a commercially available platinum foil electrode (IKA Electrochemistry Kit, purchased from IKA-Werke GmbH & Co. KG, product code: 0040005015) as cathode. RVC electrodes were sonicated in acetone (30 min) and oven-dried (160 °C) overnight prior to use. Platinum foil electrodes were sonicated both in acetone (30 min) and HCl 2 M (1 h), rinsed thoroughly with distilled water and acetone, and dried with a heat gun prior to use. Reactions were conducted in commercially available undivided cells (IKA Electrochemistry Kit, vial 10 mL, 18 mm (internal diameter) x 64 mm, purchased from IKA-Werke GmbH & Co. KG, product code: 0040003170).

2. Synthesis of starting materials

The oxidant $(Bu_4N)_2S_2O_8$ was prepared in accordance with the literature.[70] Aryl disulfides that were not commercially available (Ar_2S_2; Ar = 4-trifluoromethylphenyl, 3-fluorophenyl, 3-chlorophenyl, 4-chlorophenyl) were prepared following literature precedent.[62,63] Because the synthesis of *bis*(4-chlorophenyl) disulfide forms part of our model, "closed loop" thiol recycling strategy, this particular synthesis is described in full in the following section.

2.1. Synthesis of *bis*(4-chlorophenyl) disulfide[62,63]

To a stirring suspension of 4-chlorothiophenol (7.15 g, 49 mmol, 1 eq.) and potassium iodide (0.81 g, 4.9 mmol, 0.1 eq.) in EtOAc (60 mL) was added dropwise *via* syringe an aqueous solution of 34.5–36.5% hydrogen peroxide (4.3 mL, 49 mmol, 1 eq.). Upon this addition an immediate color change was observed (from colorless to orange/brown). After the addition was completed, the reaction was left stirring at room temperature for 30 min. A saturated solution of sodium thiosulfate was then added until the orange/brown color disappeared. The resulting reaction mixture was extracted with EtOAc (2 × 10 mL) and the combined organic layers washed with brine and dried over magnesium sulfate. The solvent was then removed *in vacuo* to provide the target product as a pale yellow powder (6.43 g, 91%).

^{1}H NMR (300 MHz, 300 K, $CDCl_3$): δ = 7.36-7.43 (4H, m), 7.25-7.31 ppm (4H, m).

^{13}C{^{1}H} NMR (75 MHz, 300 K, $CDCl_3$): δ = 135.3 (s), 133.8 (s), 129.5 ppm (s).

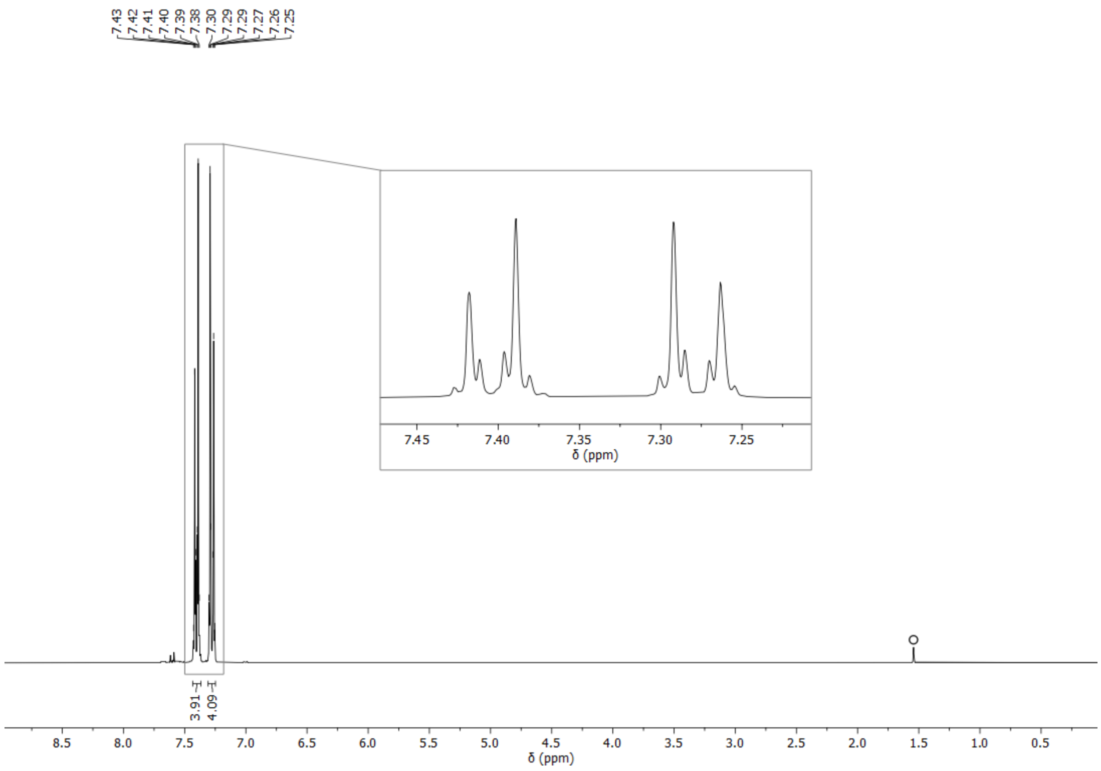

Figure S2. ^{1}H NMR spectrum of *bis*(4-chlorophenyl) disulfide in $CDCl_3$; ○ = H_2O.

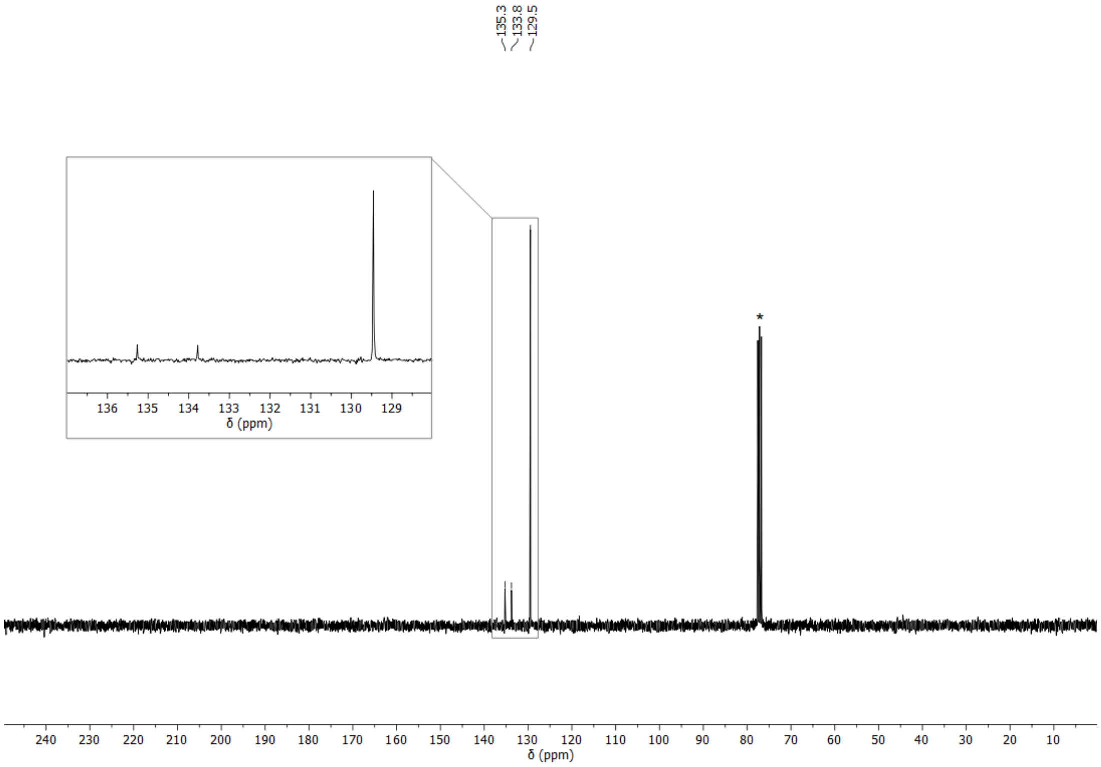

Figure S3. $^{13}C\{^{1}H\}$ NMR spectrum of *bis*(4-chlorophenyl) disulfide in $CDCl_3$ (*).

3. Stoichiometric reaction development

3.1. Generation of $(ArS)_3P$ from P_4 and Ar_2S_2

3.1.1. Transformation and *in situ* measurement of conversion

To a suspension of bis(4-chlorophenyl)disulfide (18.7 mg, 0.065 mmol, 1.67 eq. per P atom) and $KN(SiMe_3)_2$ (0.2 mg, 0.001 mmol, 0.025 eq. per P atom) in MeCN (0.5 mL) was added a stock solution of P_4 (0.01 mmol, 100 µL, 0.1 M in C_6H_6). After stirring for 30 min, an internal standard of Ph_3PO (0.02 mmol, 250 µL, 0.08 M in MeCN) was added. The reaction was transferred to an NMR tube equipped with a C_6D_6 capillary, and submitted for $^{31}P\{^1H\}$ NMR spectroscopy.

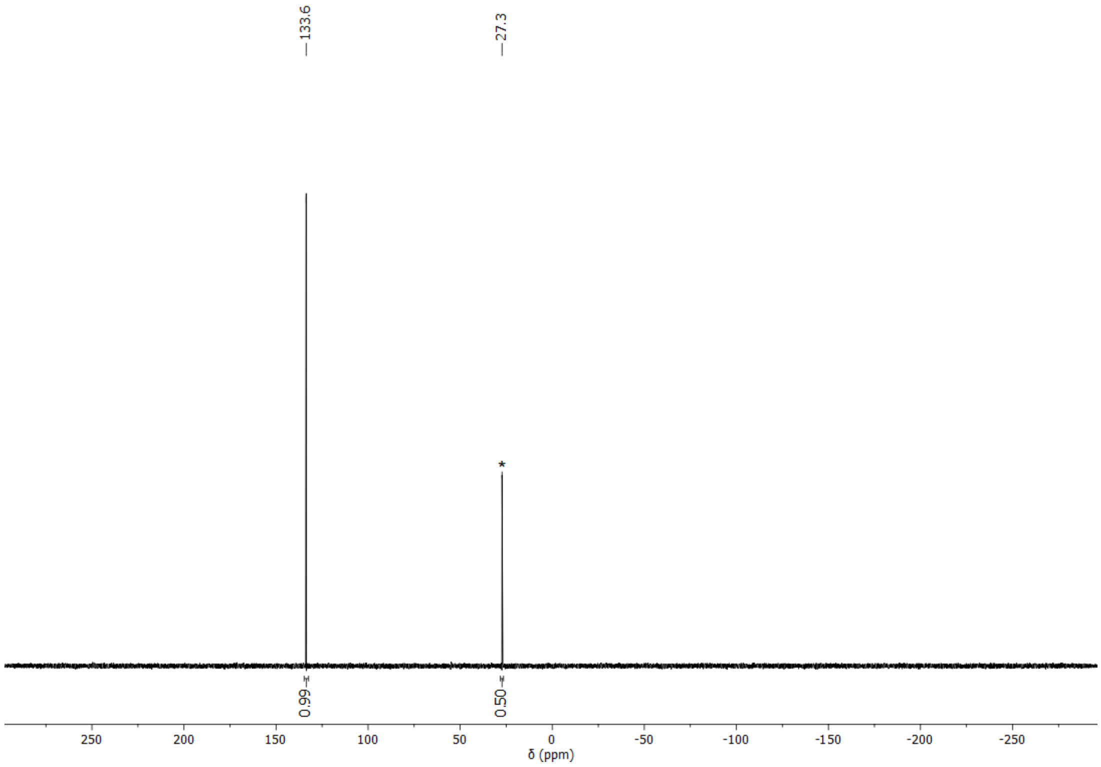

Figure S4. Quantitative single-scan $^{31}P\{^1H\}$ NMR spectrum of tris(4-chlorophenyl)thiophosphite generated *in situ* from P_4 in MeCN. * = Ph_3PO (internal standard, 0.02 mmol).

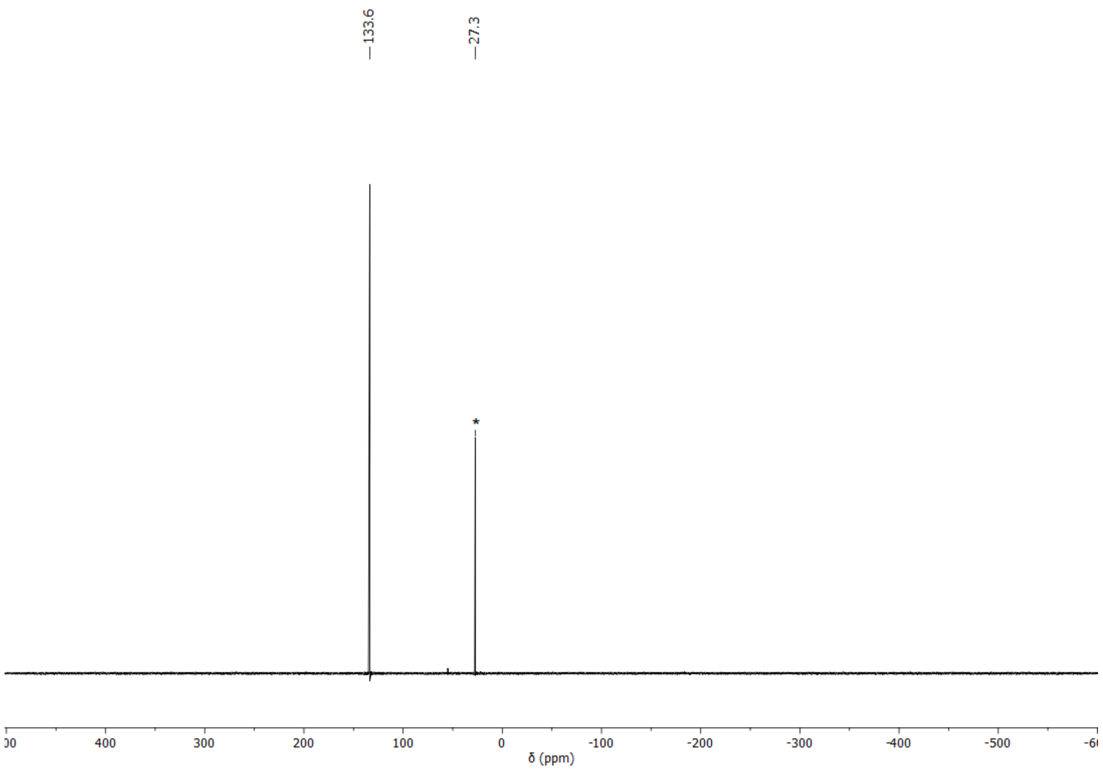

Figure S5. $^{31}P\{^{1}H\}$ NMR spectrum of tris(4-chlorophenyl)thiophosphite generated *in situ* from P_4 in MeCN. * = Ph_3PO (internal standard, 0.02 mmol). The spectral width of the spectrum extends to −600 ppm to illustrate the quantitative consumption of P_4, which resonates at ca. −520 ppm.

3.1.2. Synthesis and isolation of $(ArS)_3P$

Synthesis and isolation of tris(4-chlorophenyl)thiophosphite from P_4

A solution of bis(4-chlorophenyl)disulfide (862 mg, 3.0 mmol, 1.5 eq. per P atom) and $KN(SiMe_3)_2$ (9 mg, 0.05 mmol, 0.025 eq. per P atom) in MeCN (50 mL) was added to a stirring suspension of P_4 (62 mg, 0.50 mmol) in MeCN (10 mL). The resulting mixture was stirred for 18 hours. Volatiles were removed *in vacuo* and the resulting solid was washed with *n*-hexane (10 mL) to give tris(4-chlorophenyl)thiophosphite (758 mg, 1.64 mmol, 82%) as a white powder.

^{1}H NMR (400 MHz, C_6D_6) δ 7.14 – 7.10 (m, 6H), 6.89 – 6.84 ppm (m, 6H).

$^{31}P\{^{1}H\}$ NMR (162 MHz, C_6D_6) δ 133.4 ppm (s).

$^{13}C\{^{1}H\}$ NMR (101 MHz, C_6D_6) δ 135.9 (d, J_{CP} = 4.4 Hz), 135.5 (d, J_{CP} = 2.6 Hz), 130.3 (d, J_{CP} = 12.5 Hz), 129.7.

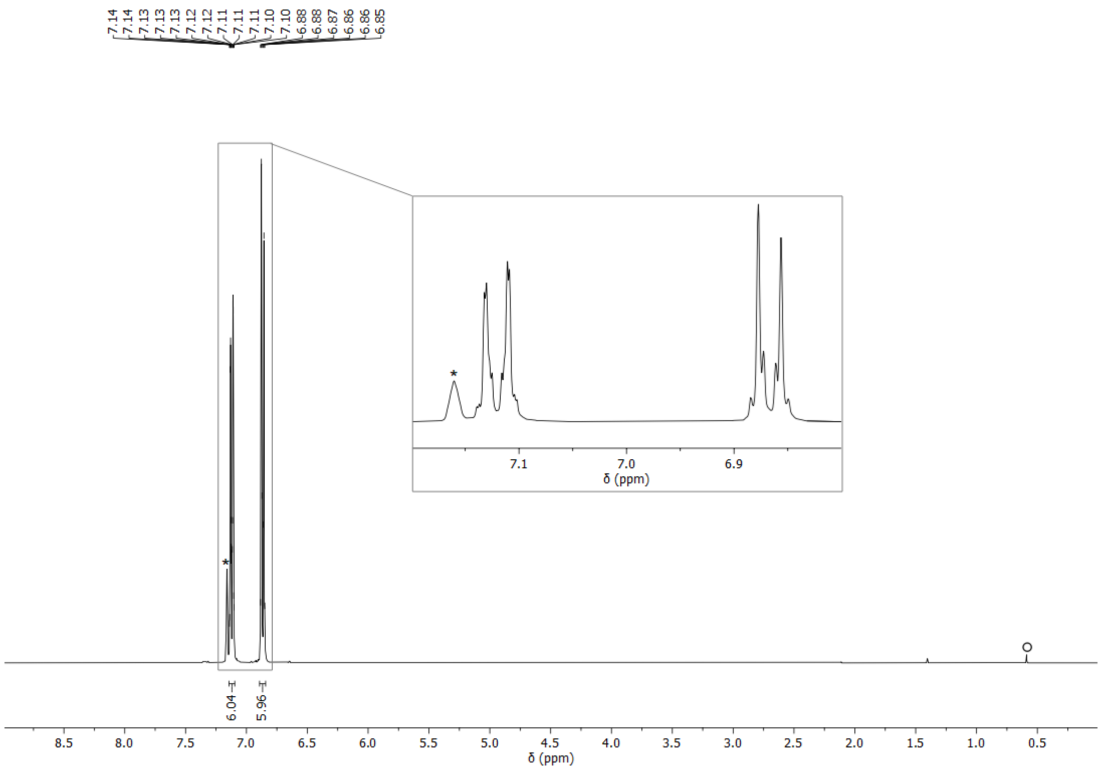

Figure S6. ^{1}H NMR spectrum of tris(4-chlorophenyl)thiophosphite (from P_4) in C_6D_6 (*); ○ = MeCN.

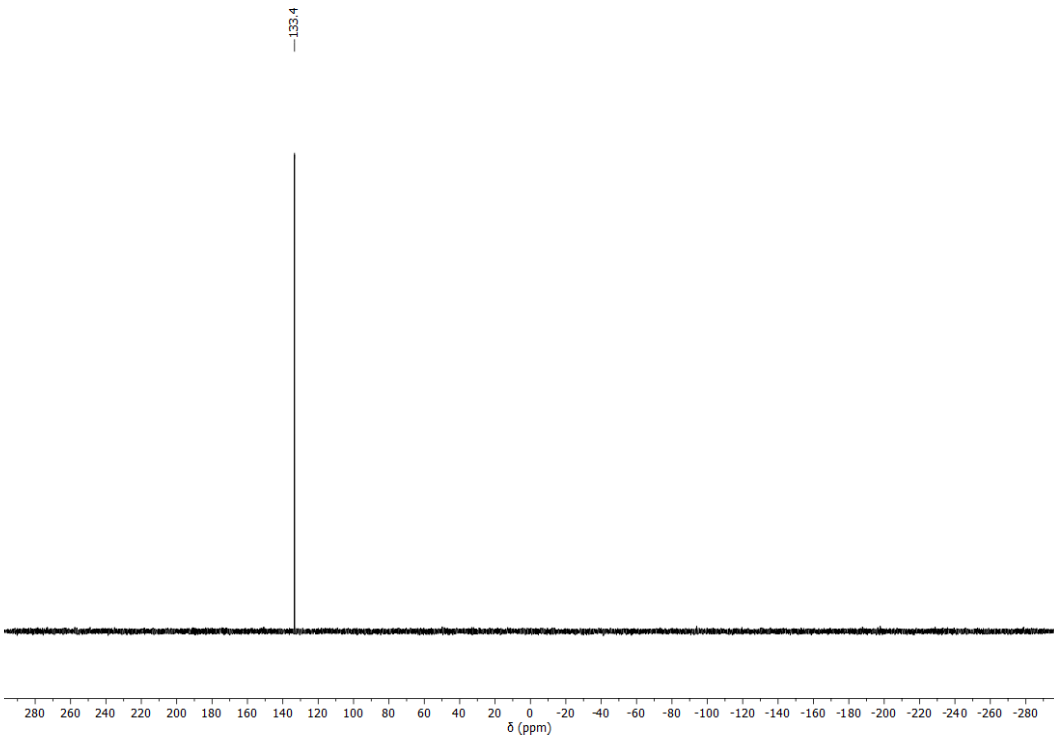

Figure S7. $^{31}P\{^1H\}$ NMR spectrum of tris(4-chlorophenyl)thiophosphite (from P_4) in C_6D_6.

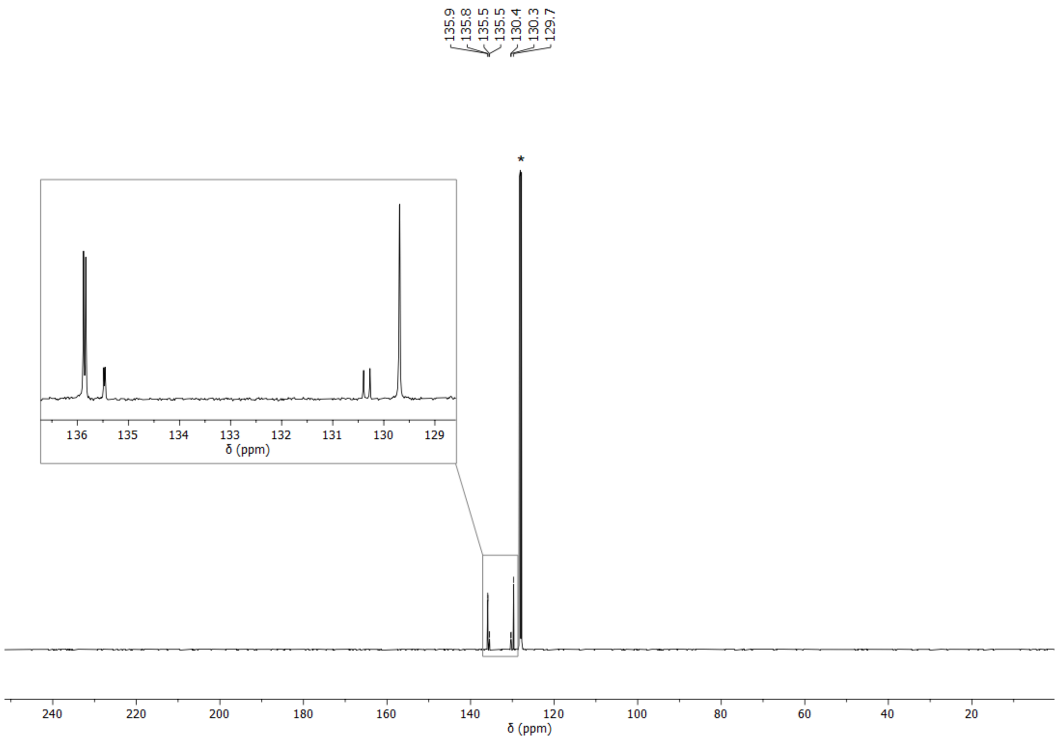

Figure S8. $^{13}C\{^1H\}$ NMR spectrum of tris(4-chlorophenyl)thiophosphite (from P_4) in C_6D_6 (*).

Synthesis and isolation of tris(4-chlorophenyl)thiophosphite from P_{red}

With excess P_{red}:

MeCN (50 mL) was added to a round-bottomed Schlenk flask charged with a stirrer bar, P_{red} (310 mg, 10 mmol, 5 eq.), bis(4-chlorophenyl)disulfide (862 mg, 3.0 mmol, 1.5 eq.), and $KN(SiMe_3)_2$ (40 mg, 0.2 mmol, 0.1 eq.). A reflux condenser was attached, and the mixture was stirred under reflux for 7 days. Upon cooling, the mixture was filtered. Volatiles were removed from the filtrate *in vacuo* to give pale yellow solid residues. These were extracted with *n*-hexane (2 × 30 mL). Removal of the *n*-hexane *in vacuo* provided tris(4-chlorophenyl)thiophosphite (660 mg, 1.43 mmol, 71% based on $^{Cl}Ar_2S_2$) as a white microcrystalline solid. NMR data are identical to those of the same product prepared from P_4.

With stoichiometric P_{red}:

In a glovebox, a photoreactor tube was charged with a stirrer bar, bis(4-chlorophenyl)disulfide (46.0 mg, 0.16 mmol, 1.6 eq.), $KN(SiMe_3)_2$ (2.0 mg, 0.01 mmol, 0.1 eq.) and red phosphorus (3.1 mg, 0.1 mmol). MeCN (0.6 mL) was added, and the resulting suspension was removed from the glovebox and heated to 80 °C. After stirring for 7 days at this temperature, the reaction was brought into the glove box. The reaction mixture was filtered and transferred to an NMR tube equipped with a C_6D_6 capillary. An internal standard of Ph_3PO

(0.02 mmol, 250 μL, 0.08 M in MeCN) was added, and the solution was submitted for $^{31}P\{^{1}H\}$ NMR spectroscopy. The resulting spectrum is shown in Figure S9, below.

Note that this procedure is less attractive than the prodecure with excess P_{red} (above) for preparative synthesis of $(^{Cl}ArS)_3P$ due to difficulties in separating the product from residual unreacted $^{Cl}Ar_2S_2$.

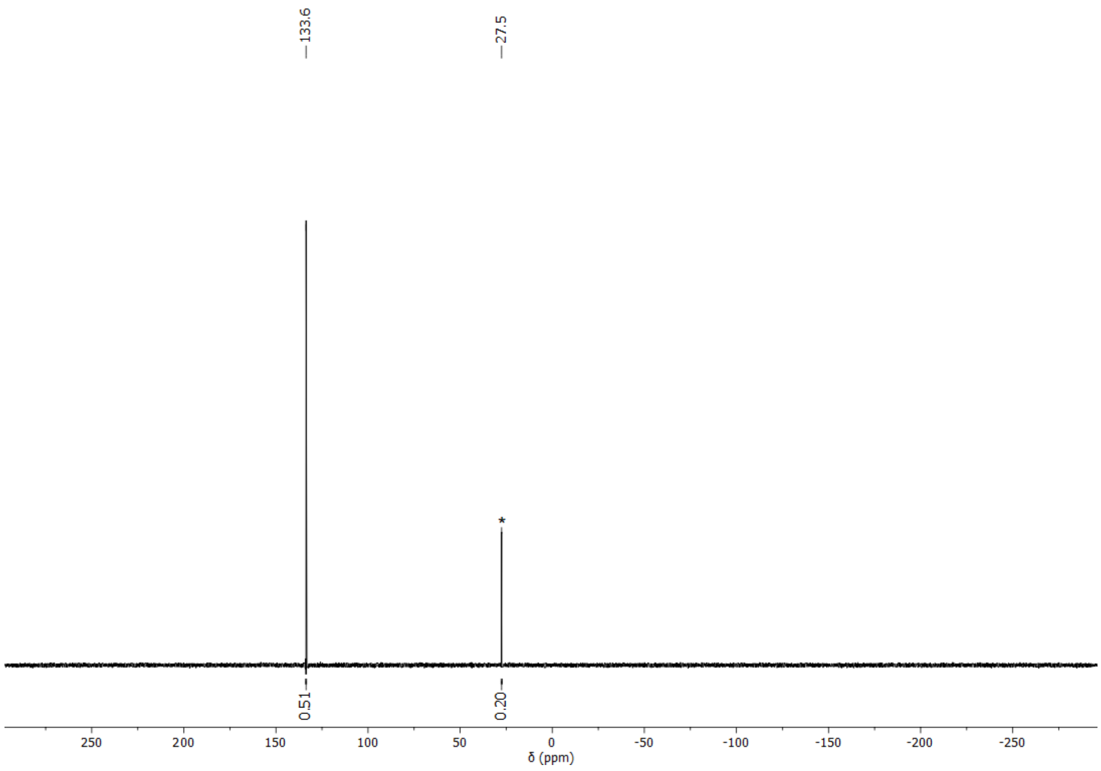

Figure S9. Quantitative single-scan $^{31}P\{^{1}H\}$ NMR spectrum of tris(4-chlorophenyl)thiophosphite generated *in situ* from of P_{red} in MeCN. * = Ph_3PO (internal standard, 0.02 mmol).

3.2. One pot synthesis of tertiary phosphines from P_4 *via* $(ArS)_3P$

3.2.1. Synthesis and isolation of Ph_3P

MeCN (40 mL) was added to a Schlenk tube charged with a solid mixture of P_4 (0.94 mmol, 116 mg), bis(4-chlorophenyl)disulfide (5.71 mmol, 1.64 g, 1.53 eq. per P atom), and $KN(SiMe_3)_2$ (0.09 mmol, 16 mg, 0.025 eq. per P atom). The resulting pale yellow solution was stirred overnight. Volatiles were removed *in vacuo*, and the resulting solid residues were redissolved in THF (10 mL). The solution was cooled to –78 °C

and phenylmagnesium bromide (13 mmol, 13 mL, 1.0 M in THF, 3.5 eq. per P atom) was added dropwise. The resulting suspension was thawed to room temperature, giving a yellow solution. After 2 h, volatiles were removed *in vacuo*. Ph_3P was obtained as a white powder from the resulting solid residue by sublimation (2.7 mmol, 0.70 g, 70%). Grinding of the residue to a fine powder facilitated more efficient sublimation, as the residual magnesium salts otherwise formed solid lumps from which the product was not readily extruded.

^{1}H NMR (400 MHz, C_6D_6) δ 7.43 – 7.35 (m, 6H), 7.09 – 7.01 ppm (m, 9H).

^{31}P{^{1}H} NMR (162 MHz, C_6D_6) δ -4.7 ppm (s).

^{13}C{^{1}H} NMR (101 MHz, C_6D_6) δ 138.1 (d, J_{CP} = 12.2 Hz), 134.2 (d, J_{CP} = 19.8 Hz), 128.9 (s), 128.8 ppm (s).

These NMR data are commensurate with previous literature reports.[51]

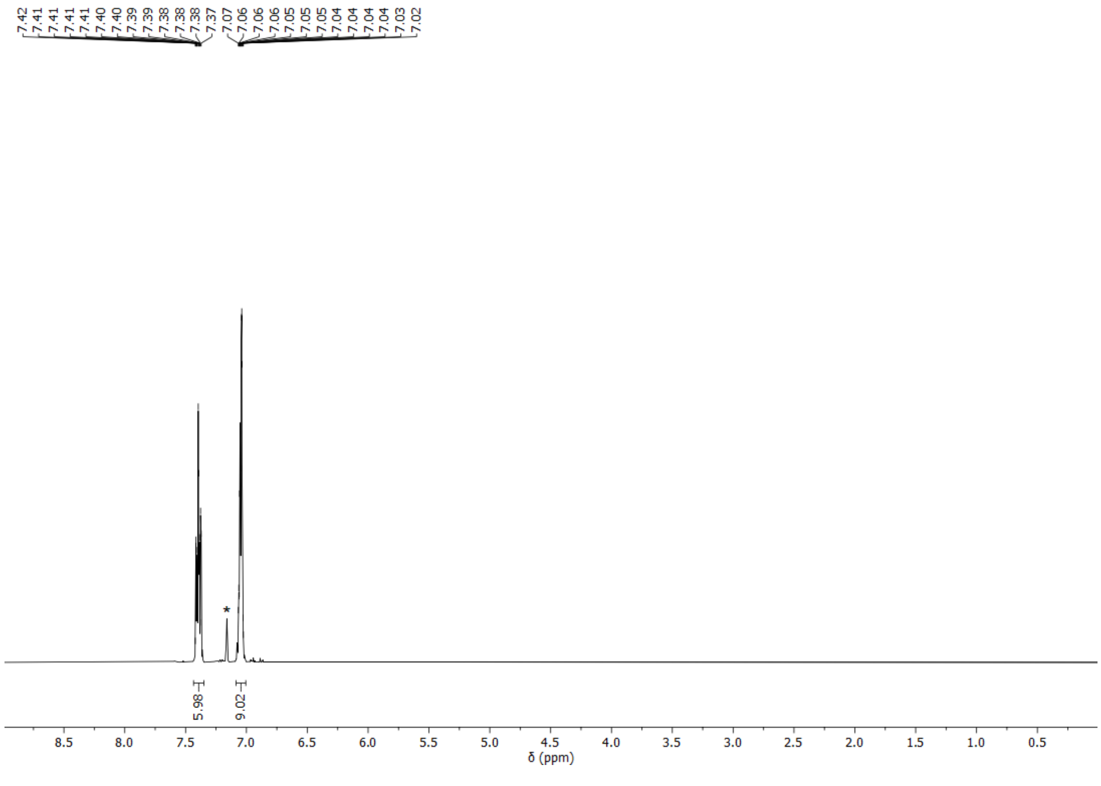

Figure S10. ^{1}H NMR spectrum of triphenylphosphine in C_6D_6 (*).

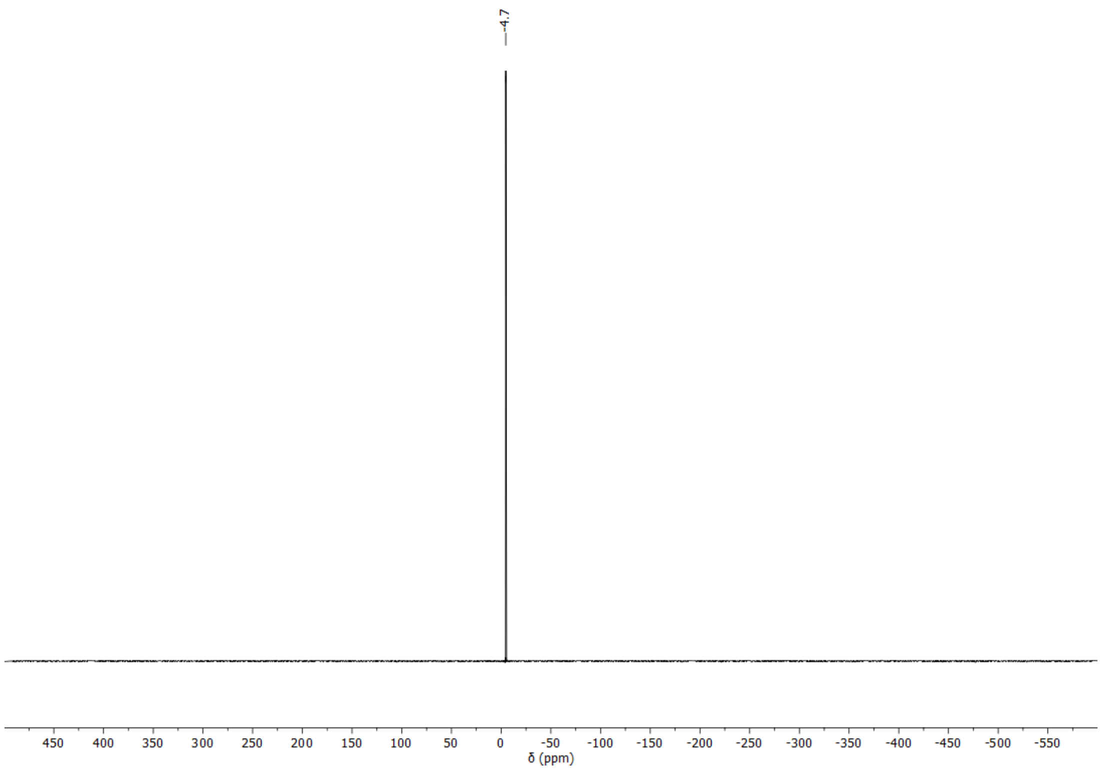

Figure S11. $^{31}P\{^{1}H\}$ NMR spectrum of triphenylphosphine in C_6D_6.

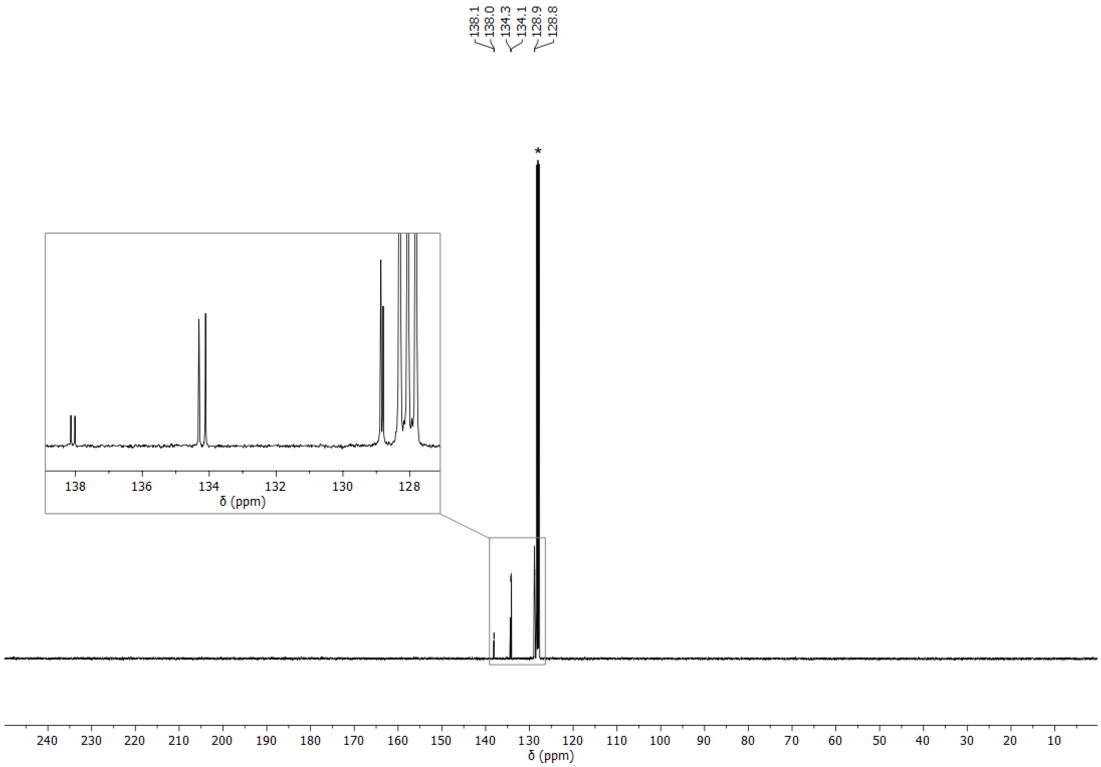

Figure S12. $^{13}C\{^{1}H\}$ NMR spectrum of triphenylphosphine in C_6D_6 (*).

3.2.2. Discussion of differences in reaction outcome using different Ar_2S_2

The use of other disulfides, such as diphenyl disulfide, was also examined for the disulfide-mediated functionalisation of P_4 under a stoichiometric regime. However, the substitution of the chloride on the aryl moiety with more electron-donating groups delivered less productive reactivity. While the trithiophosphites were still readily generated from P_4 and the corresponding diaryl disulfides, subsequent treatment of the trithiophosphites with phenylmagnesium bromide gave triphenylphosphine (**A** in Figure S13, below) as the major product only from tris(4-chlorophenyl)thiophosphite. For the other trithiophosphites, an unidentified species (**B** in Figure S13) or tetraphenyldiphosphine (**C** in Figure S13)[71] were generated as the major products, with little or no formation of the desired product. As such, the use of bis(4-chlorophenyl)disulfide was maintained for all subsequent stoichiometric reactions.

We speculate that for more electron-rich disulfides, the poorer leaving group ability of the thiolate moieties leads to reduced regioselectivity for Grignard attack, with (desired) attack at P competing with (undesired) attack at S. The latter would result in extrusion of a nucleophilic R_2P^- fragment (e.g. Ph_2PMgBr, $(ArS)_2PMgBr$) that could form a new P–P bond *via* attack at P of a second P–S bond, ultimately leading to the observed P_2Ph_4.

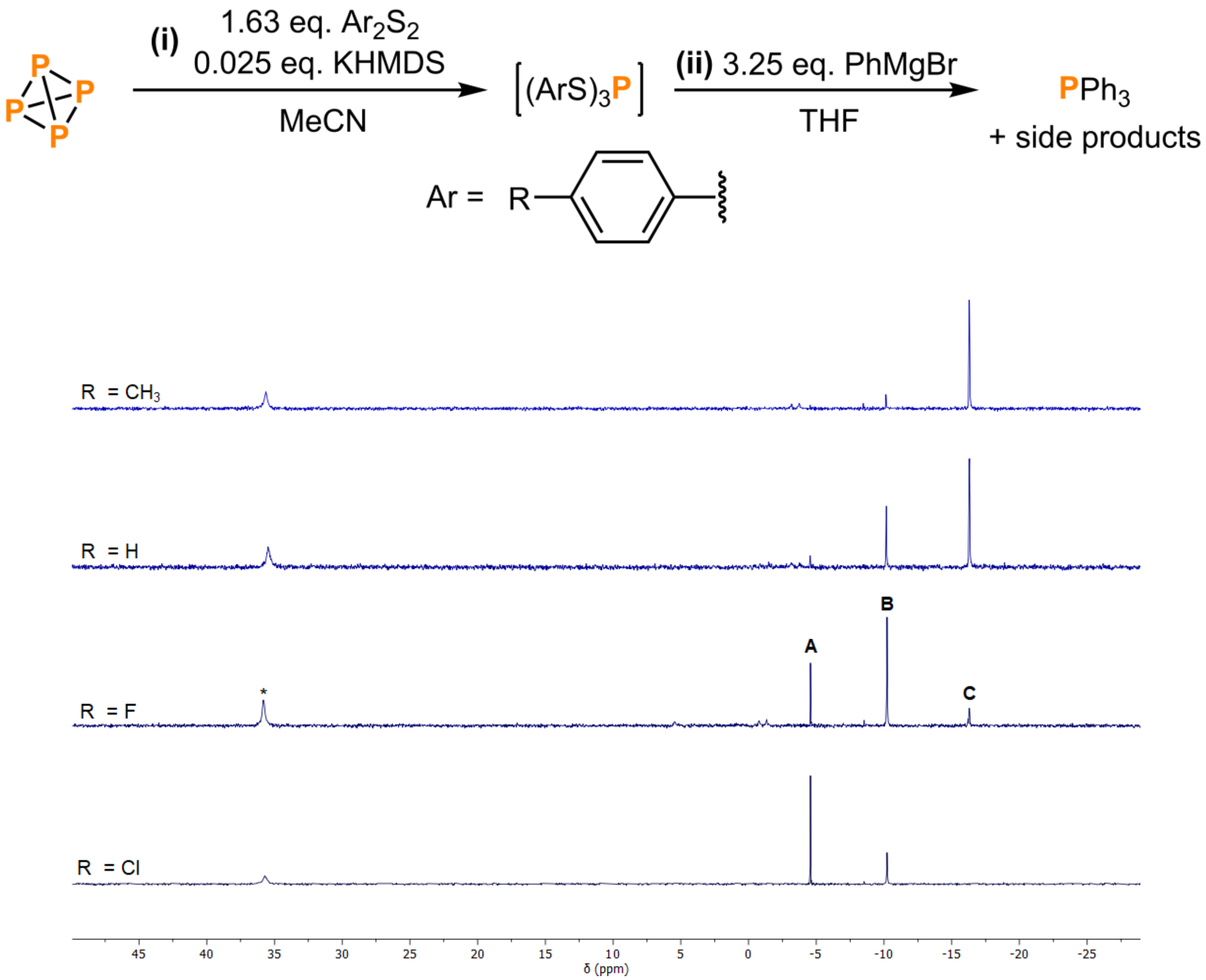

Figure S13. $^{31}P\{^{1}H\}$ NMR spectra of the reactions between different trithiophosphites, generated from diaryl sulfides and P_4, with PhMgBr. * = Ph_3PO (internal standard, 0.02 mmol).

3.2.3. Generation of R_3P and *in situ* measurement of conversion

To a solution of bis(4-chlorophenyl)disulfide (0.065 mmol, 18.7 mg, 1.63 eq. per P atom) and $KN(SiMe_3)_2$ (0.001 mmol, 0.2 mg, 0.025 eq. per P atom) in THF (0.5 mL) was added P_4 (0.01 mmol, 100 μL, 0.1 M in C_6H_6), to generate the trithiophosphite intermediate, tris(4-chlorophenyl)thiophosphate. Following 1 h of stirring, a solution of the corresponding Grignard reagent (3.5 eq. per P atom) was added as a THF solution. Following 15 min of further stirring, an internal standard of Ph_3PO (0.02 mmol, 250 μL, 0.08 M in THF) was added. The reaction was transferred to an NMR tube equipped with a C_6D_6 capillary, and submitted for $^{31}P\{^{1}H\}$ NMR spectroscopy. Conversions are summarized in Table S2.

Table S2. Conversion to R_3P measured for disulfide-mediated reactions between P_4 and Grignard reagents RMgBr.

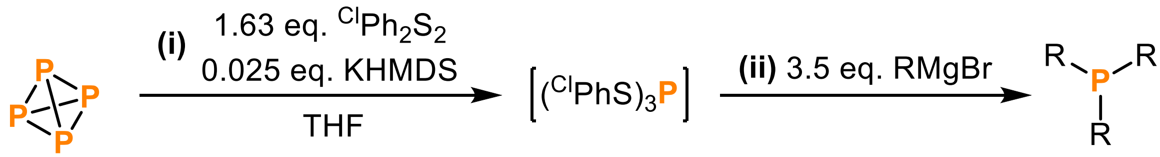

Entry	R	Conv. to R_3P / %
1	R = phenyl	93
2	R = 4-methylphenyl	81
3	R = 4-methoxyphenyl	93
4	R = 4-fluorophenyl	92
5	R = ethyl	97
6	R = phenylethynyl	72[a]
7	R = 2-thienyl	64[b]
8	R = 2-pyridyl	43[c]

[a] Conversion reached after 36 hours at room temperature.
[b] Reaction conducted on a 0.03 mmol P_4 scale.
[c] The Mg complex of the product is generated, per previous literature description.[51]

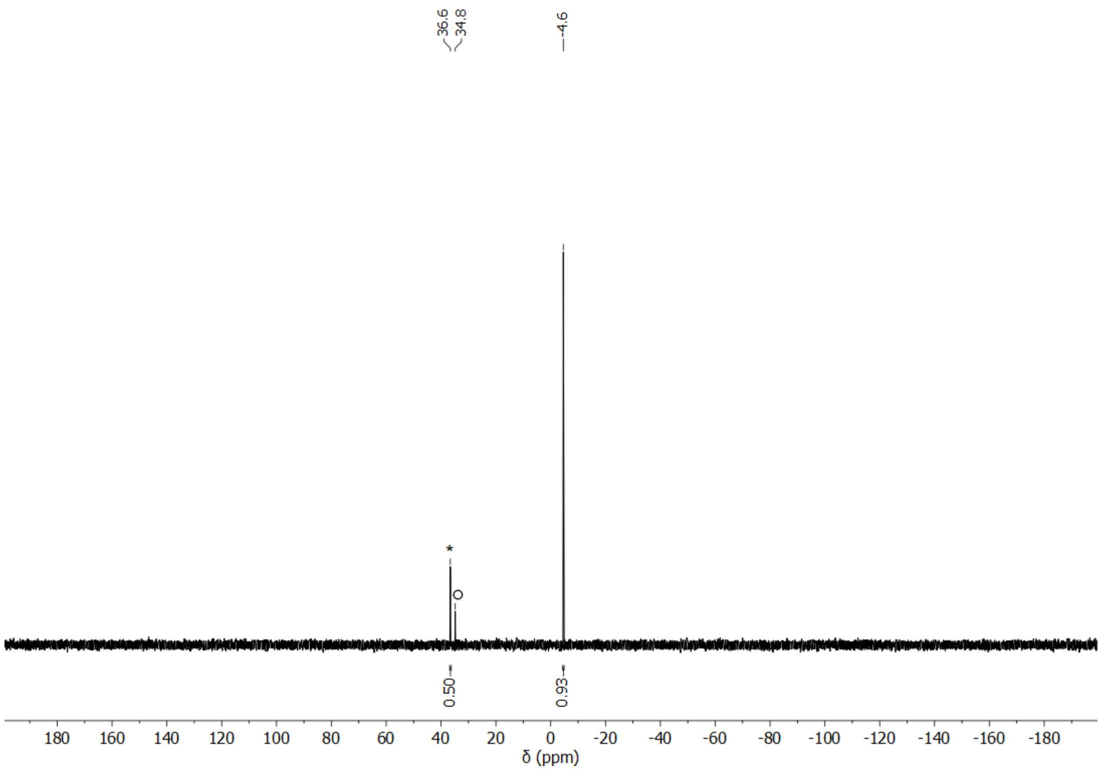

Figure S14. Quantitative single-scan $^{31}P\{^{1}H\}$ NMR spectrum of triphenylphosphine generated by the disulfide-mediated functionalisation of P_4 in THF followed by treatment with phenylmagnesium bromide. * = Ph_3PO (internal standard, 0.02 mmol) ○ = unidentified side product, possibly (^{Cl}ArS)PPh_2.

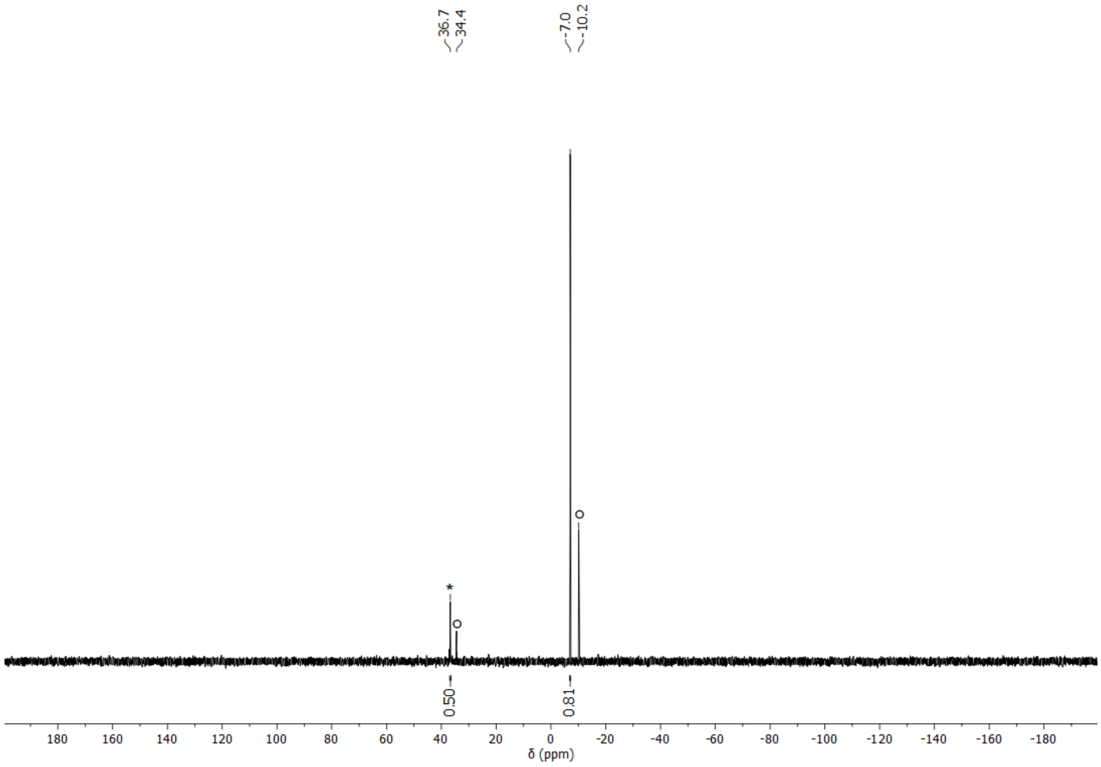

Figure S15. Quantitative single-scan $^{31}P\{^{1}H\}$ NMR spectrum of tris(4-methylphenyl)phosphine generated by the disulfide-mediated functionalisation of P_4 in THF followed by treatment with 4-methylphenylmagnesium bromide. * = Ph_3PO (internal standard, 0.02 mmol) ○ = unidentified side products.

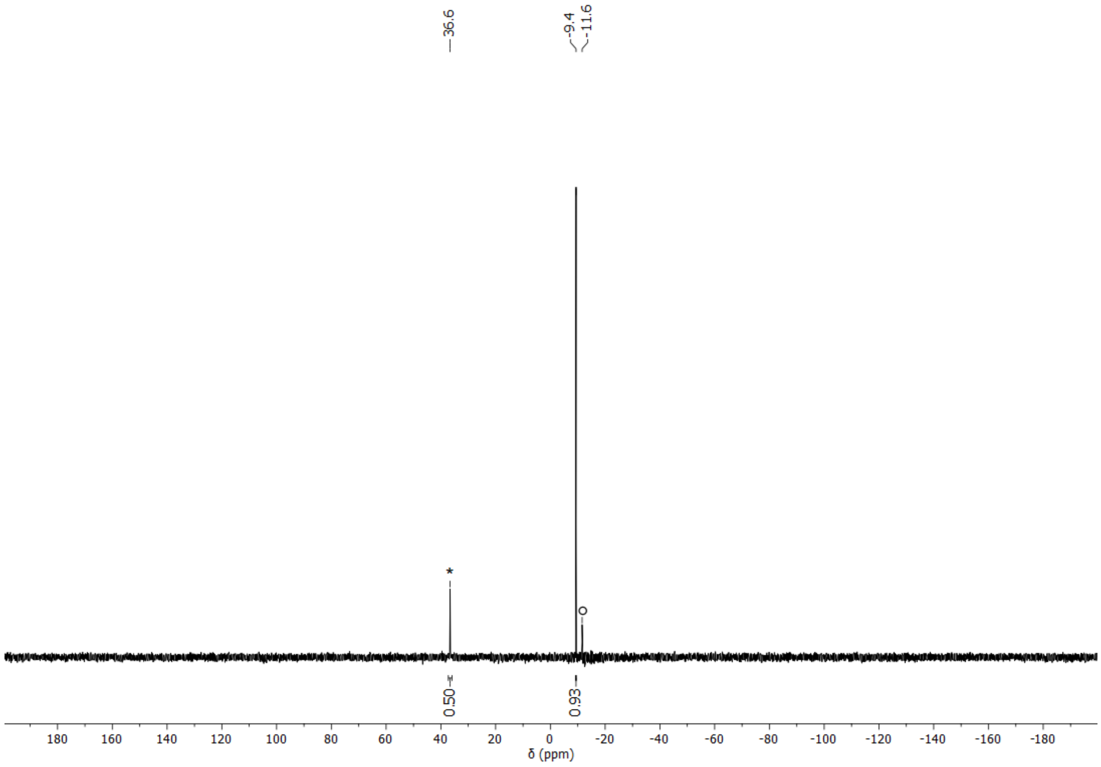

Figure S16. Quantitative single-scan $^{31}P\{^{1}H\}$ NMR spectrum of tris(4-methoxyphenyl)phosphine generated by the disulfide-mediated functionalisation of P_4 in THF followed by treatment with 4-methoxyphenylmagnesium bromide. * = Ph_3PO (internal standard, 0.02 mmol) ○ = unidentified side product.

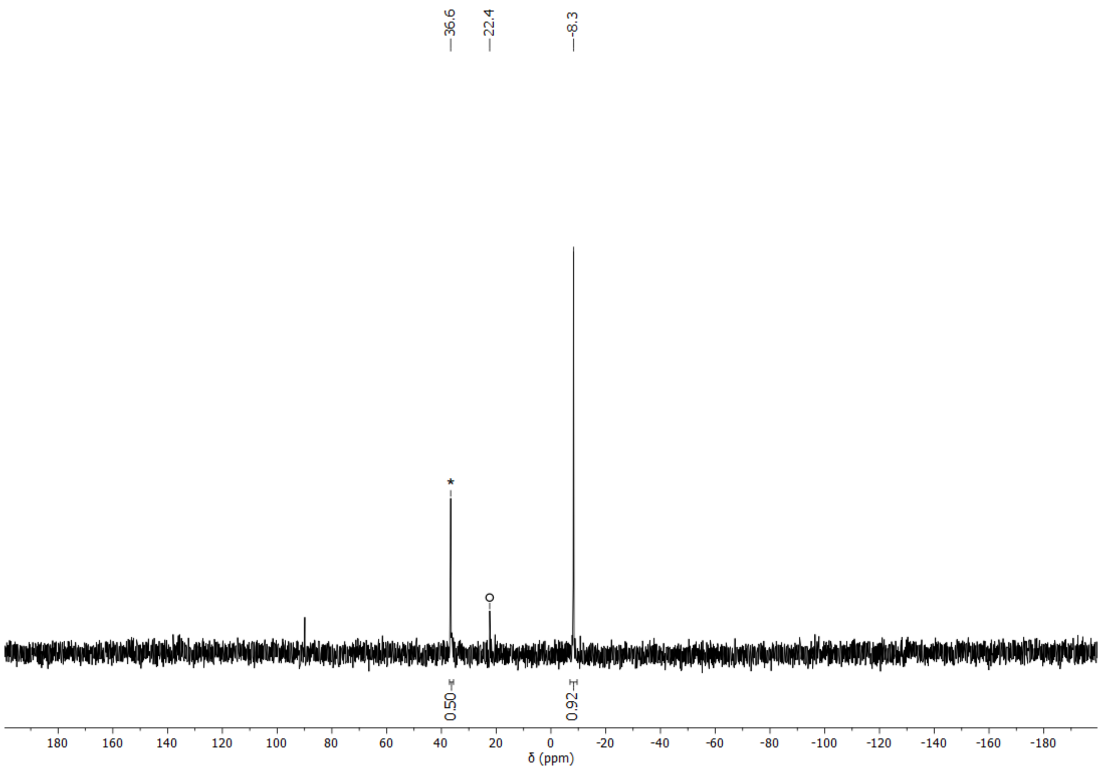

Figure S17. Quantitative single-scan $^{31}P\{^1H\}$ NMR spectrum of tris(4-fluorophenyl)phosphine generated by the disulfide-mediated functionalisation of P_4 in THF followed by treatment with 4-fluorophenylmagnesium bromide. * = Ph_3PO (internal standard, 0.02 mmol) ○ = unidentified side product.

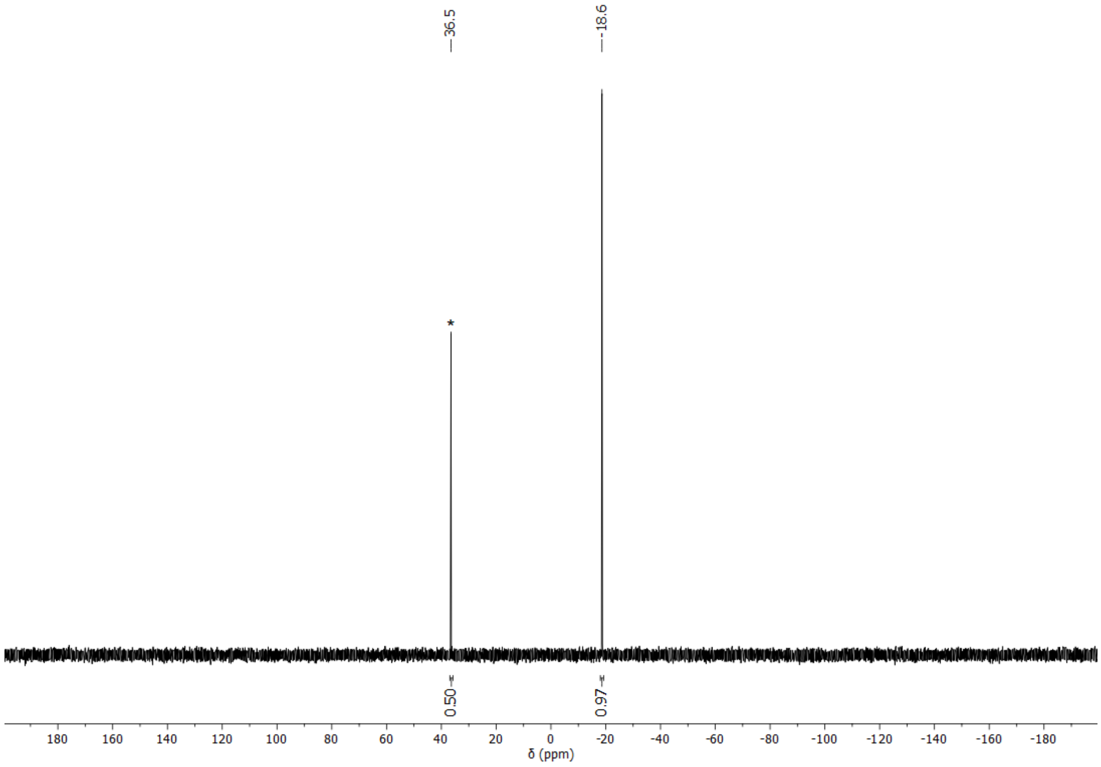

Figure S18. Quantitative single-scan $^{31}P\{^{1}H\}$ NMR spectrum of triethylphosphine generated by the disulfide-mediated functionalisation of P_4 in THF followed by treatment with ethylmagnesium bromide. * = Ph_3PO (internal standard, 0.02 mmol).

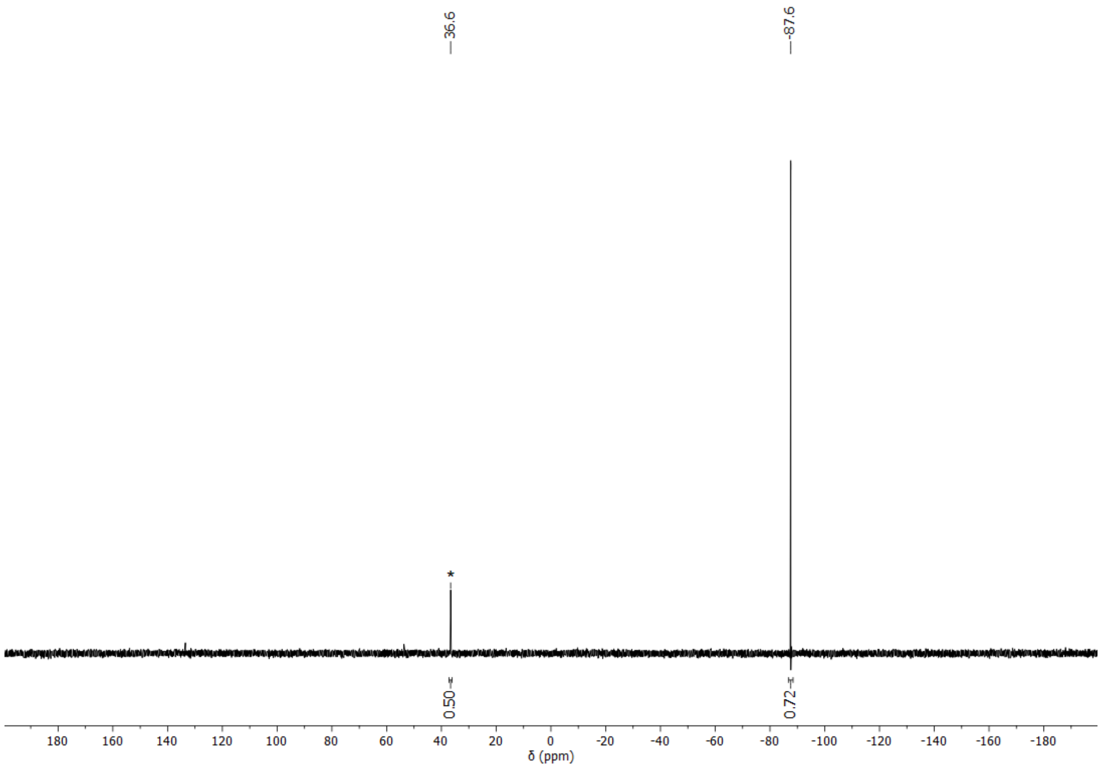

Figure S19. Quantitative single-scan $^{31}P\{^{1}H\}$ NMR spectrum of tris(phenylethynyl)phosphine generated by the disulfide-mediated functionalisation of P_4 in THF followed by treatment with phenylethynylmagnesium bromide. * = Ph_3PO (internal standard, 0.02 mmol).

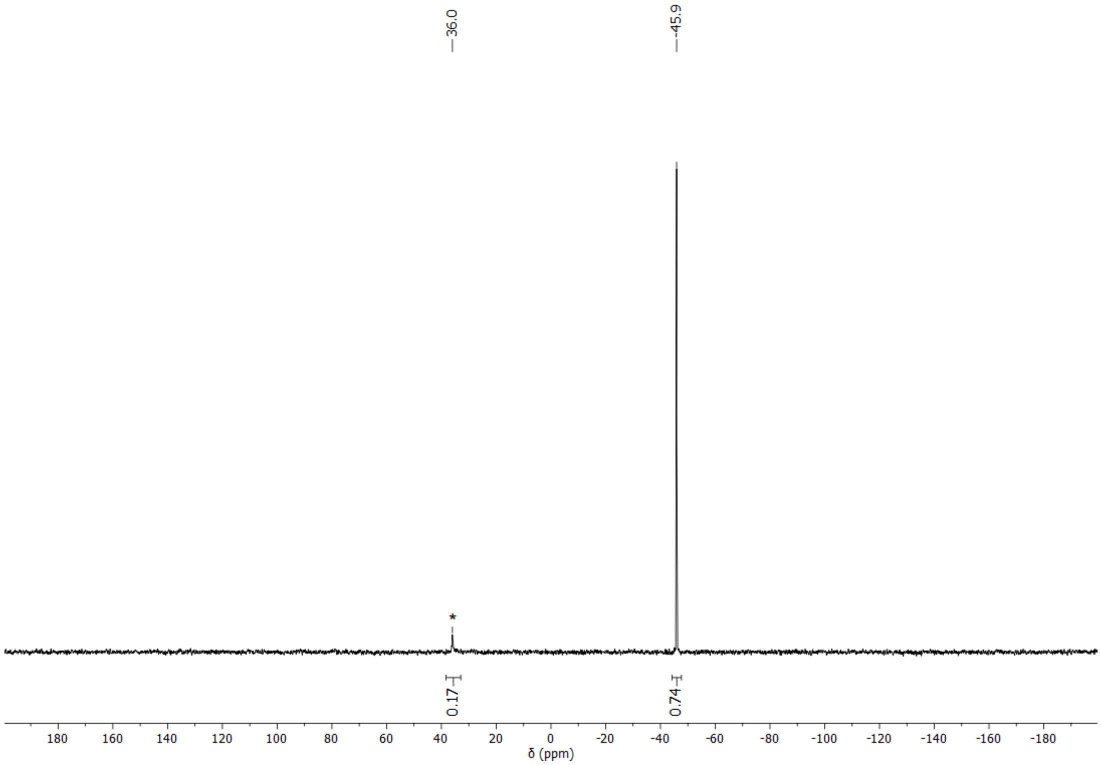

Figure S20. Quantitative single-scan $^{31}P\{^1H\}$ NMR spectrum of tris(2-thienyl)phosphine generated by the disulfide-mediated functionalisation of P_4 in THF followed by treatment with 2-thienylmagnesium bromide. * = Ph_3PO (internal standard, 0.02 mmol).

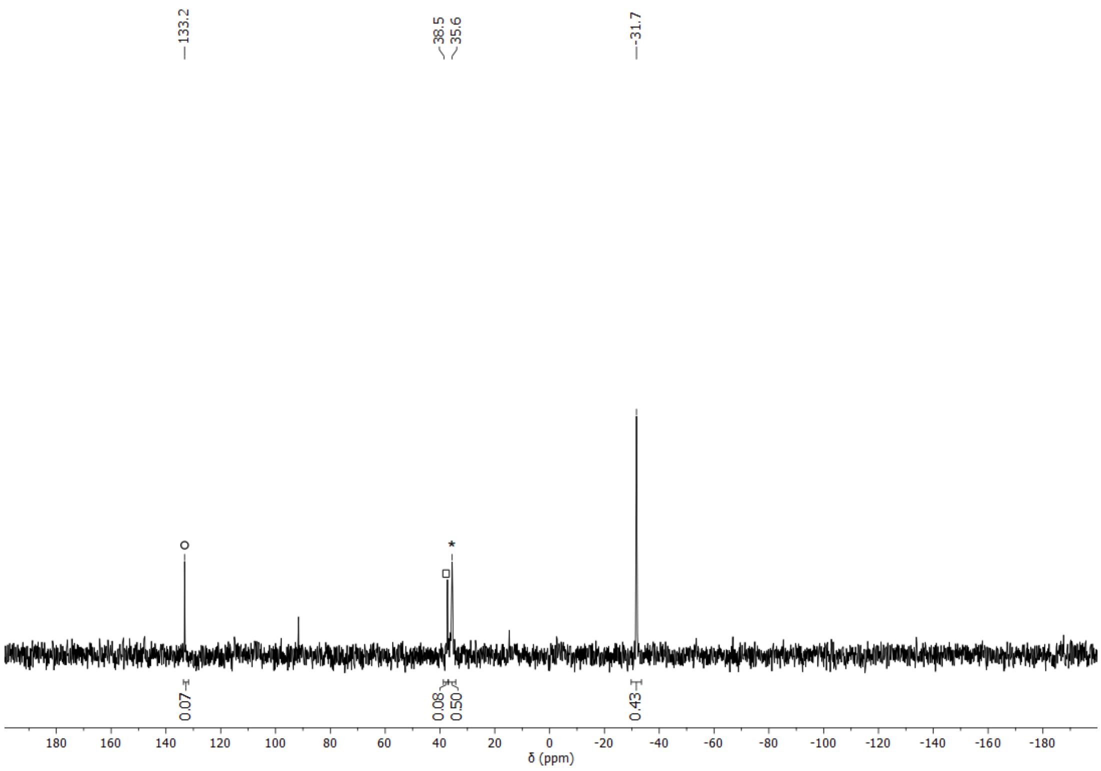

Figure S21. Quantitative single-scan $^{31}P\{^1H\}$ NMR spectrum of tris(2-pyridyl)phosphine generated by the disulfide-mediated functionalisation of P_4 in THF followed by treatment with 2-pyridylmagnesium bromide. * = Ph_3PO (internal standard, 0.02 mmol); ○ = tris(4-chlorophenyl)thiophosphite; □ = unidentified side product.

3.3. One pot synthesis of P–N bonded P_1 products from P_4 *via* $(ArS)_3P$

3.3.1. Synthesis and isolation of tris(*N*-carbazolyl)phosphine

To a Schlenk tube charged with carbazole (1.04 g, 6.2 mmol, 3.1 eq. per P atom) and $KN(SiMe_3)_2$ (1.06 g, 6.2 mmol, 3.1 eq. per P atom) was added THF (15 mL), giving a light brown solution which was stirred for 1 h. To a separate Schlenk tube charged with bis(4-chlorophenyl)disulfide (0.89 g, 3.1 mmol, 1.55 eq. per P atom) and $KN(SiMe_3)_2$ (9 mg, 0.05 mmol, 0.025 eq. per P atom) was added THF (10 mL) and the resulting pale yellow solution was added to a solution of P_4 (62 mg, 0.5 mmol) in toluene (3 mL), which was stirred for 1 h. To this was added the intial potassium carbazolide solution dropwise at 0 °C. The resulting suspension was stirred for 1 h. Volatiles were removed *in vacuo*, giving a solid, off-white residue. The product was extracted into toluene (25 mL) and filtered. Volatiles were removed *in vacuo* from the filtrate

to give a white solid. The crude product was purified by sublimation of the impurities, yielding tris(*N*-carbazolyl)phosphine as a white solid (0.51 g, 0.96 mmol, 48 %).

^{1}H NMR (400 MHz, C_6D_6) δ 7.84 – 7.78 (m, 6H), 7.39 – 7.33 (m, 6H), 7.04 – 6.97 (m, 6H), 6.87 – 6.81 ppm (m, 6H).

^{31}P{^{1}H} NMR (162 MHz, C_6D_6) δ 78.5 ppm (s).

^{13}C{^{1}H} NMR (101 MHz, C_6D_6) δ 142.4 (d, J_{CP} = 9.2 Hz), 127.4 (s), 127.1 (d, J_{CP} = 2.4 Hz), 122.5 (s), 120.7 (s), 113.4 ppm (d, J_{CP} = 12.9 Hz).

These NMR data are commensurate with previous literature reports.[72]

The solid that remained following the toluene extraction was suspended in Et_2O (30 mL), quenched with saturated aqueous ammonium chloride solution, and extracted into further Et_2O (2 × 40 mL). The combined organic layers were washed with brine, dried over $MgSO_4$, and filtered. Volatiles were removed *in vacuo* to give 4-chlorothiophenol (0.82 g, 5.7 mmol, 91 %).

^{1}H NMR (300 MHz, $CDCl_3$) δ 7.21 (s, 4H), 3.45 ppm (s, 1H).

^{13}C{^{1}H} NMR (75 MHz, $CDCl_3$) δ 131.9 (s), 130.9 (s), 129.3 (s), 118.3 ppm (s).

These NMR data are commensurate with previous literature reports.[73]

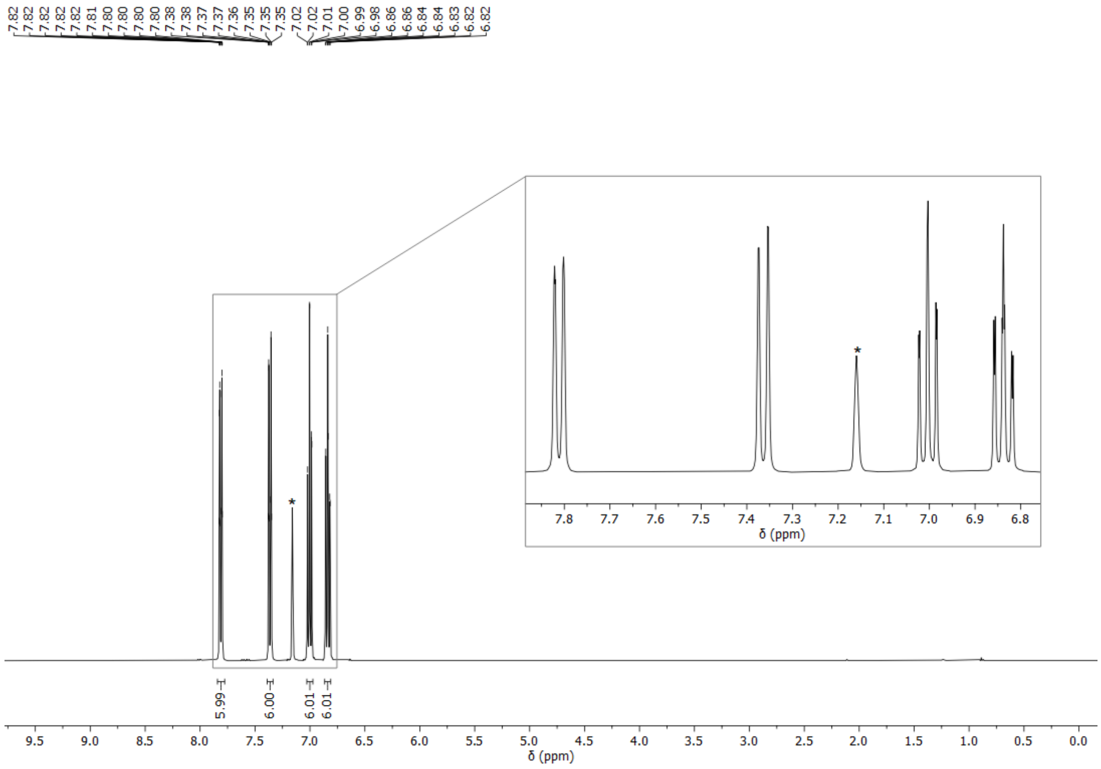

Figure S22. ^{1}H NMR spectrum of tris(*N*-carbazolyl)phosphine in C_6D_6 (*).

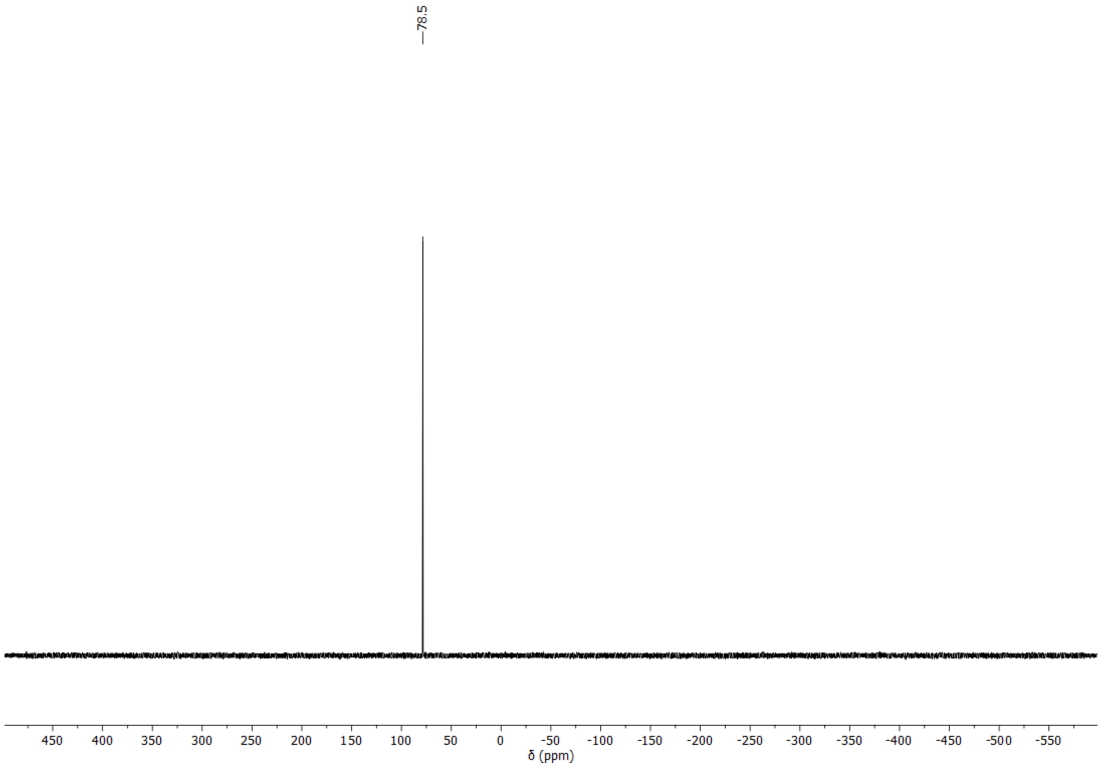

Figure S23. $^{31}P\{^{1}H\}$ NMR spectrum of tris(*N*-carbazolyl)phosphine in C_6D_6.

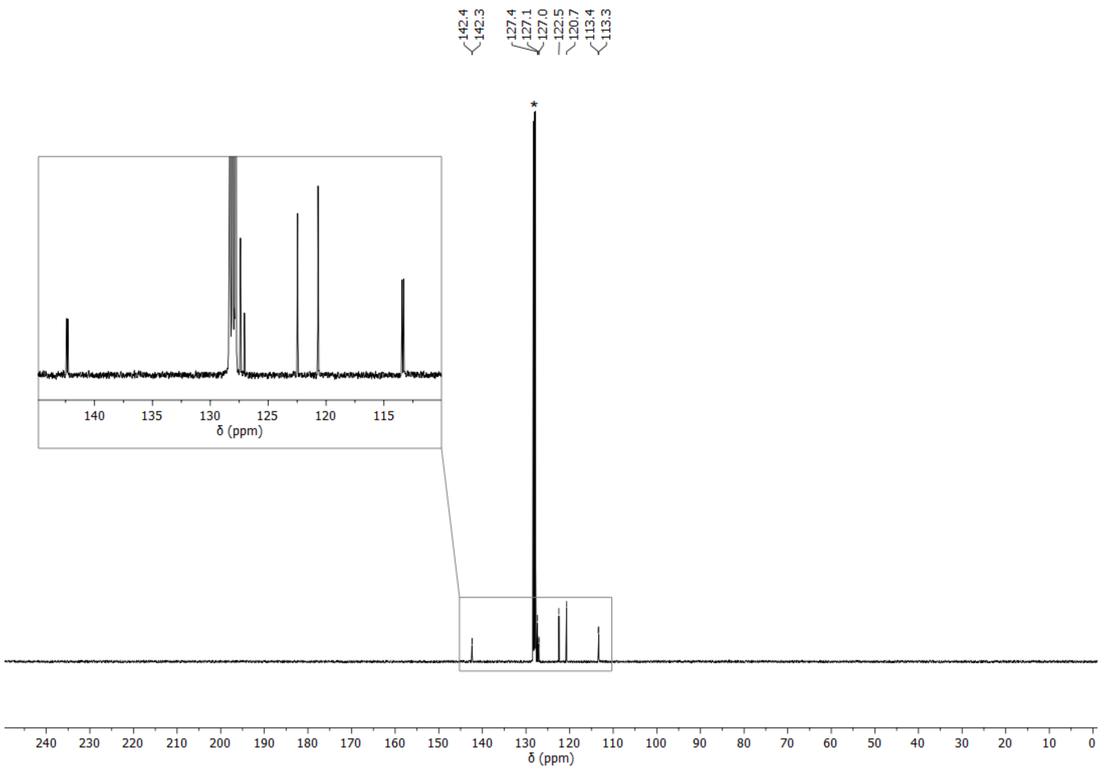

Figure S24. $^{13}C\{^{1}H\}$ NMR spectrum of tris(*N*-carbazolyl)phosphine in C_6D_6 (*).

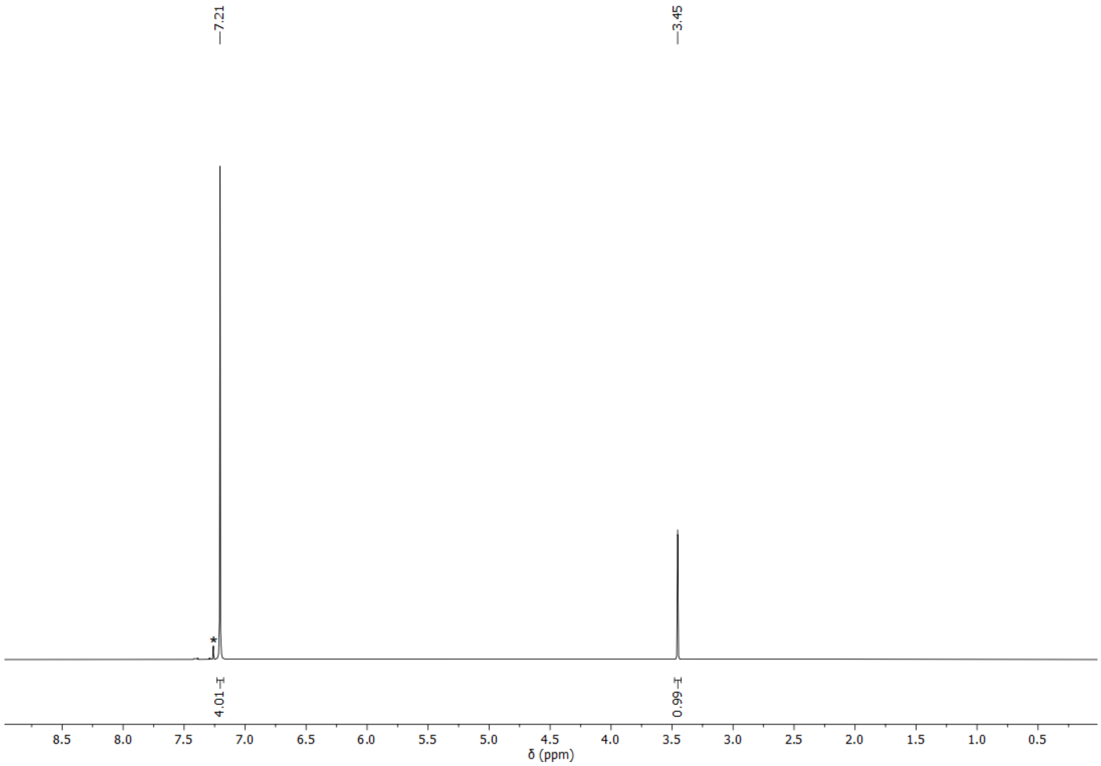

Figure S25. ^{1}H NMR spectrum of 4-chlorothiophenol in $CDCl_3$ (*).

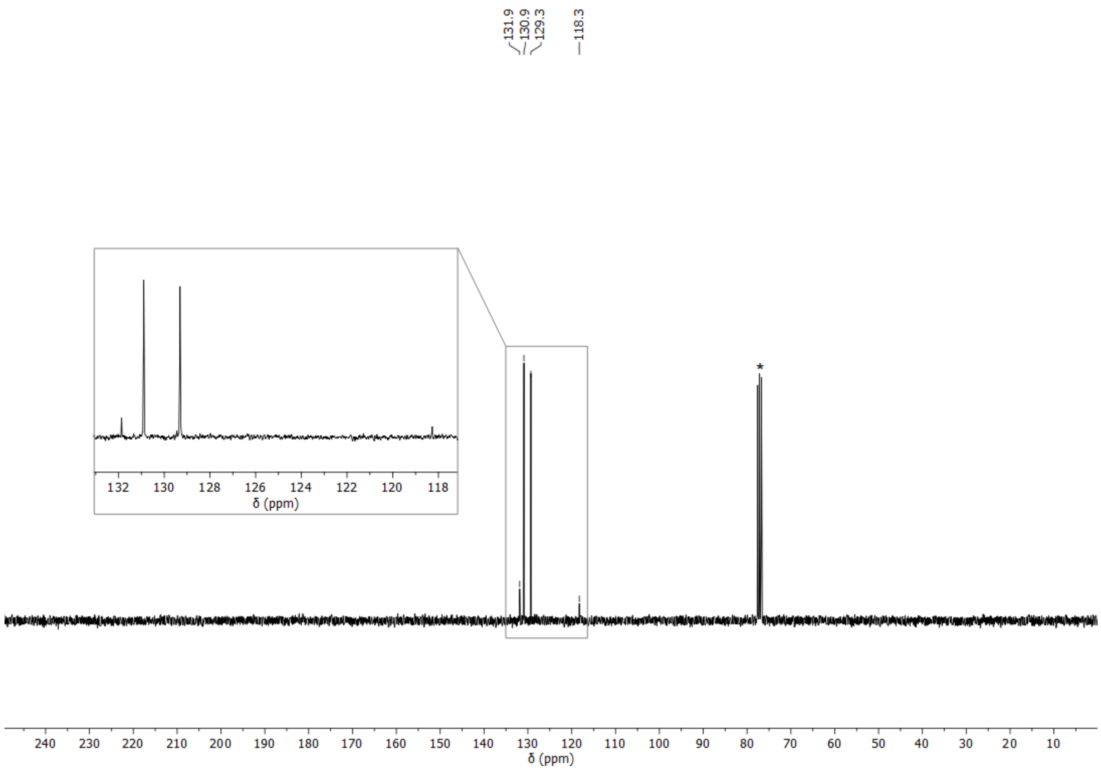

Figure S26. $^{13}C\{^{1}H\}$ NMR spectrum of 4-chlorothiophenol in $CDCl_3$ (*).

3.3.2. Generation of P–N bonded P_1 products and *in situ* measurement of conversion

Representative procedure for the generation of $(R_2N)_3P$:

To a solution of bis(4-chlorophenyl)disulfide (0.065 mmol, 18.7 mg, 1.63 eq. per P atom) and $KN(SiMe_3)_2$ (0.001 mmol, 0.2 mg, 0.025 eq. per P atom) in THF (0.4 mL) was added P_4 (0.01 mmol, 100 µL, 0.1 M in C_6H_6), to generate the trithiophosphite intermediate, tris(4-chlorophenyl)thiophosphite. Following 30 min of stirring, this was added to a solution of potassium amide (0.13 mmol, 3.25 eq. per P atom) in 2:1 THF:MeCN (0.6 mL), generated from the corresponding amine and $KN(SiMe_3)_2$. Following 15 min of further stirring, an internal standard of Ph_3PO (0.02 mmol, 250 µL, 0.08 M in THF) was added. The reaction was transferred to an NMR tube equipped with a C_6D_6 capillary, and submitted for $^{31}P\{^{1}H\}$ NMR spectroscopy. Conversions are summarized in Table S3.

Table S3. Conversion to $(R_2N)_3P$ measured for disulfide-mediated reactions between P_4 and amides R_2NK.

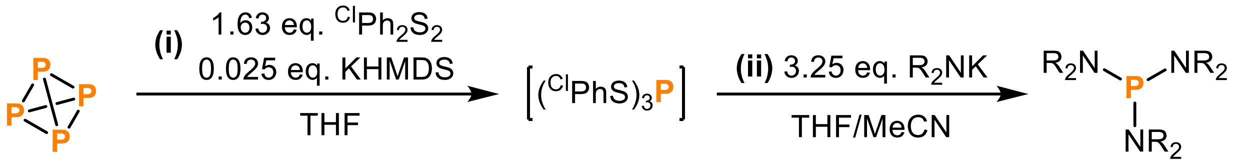

Entry	R	Conv. to $(R_2N)_3P$ / %
1	R_2N = carbazolyl	75
2	R_2N = 3,5-dimethylpyrazolyl	50
3	R = phenyl	60[a]
4	R = phenyl	55[b]

[a] The disubstituted product, $(^{Cl}PhS)P(NPh_2)_2$, generated instead.
[b] Conversion reached after 16 h at 60 °C. 26% of the disubstituted product, $(^{Cl}PhS)P(NPh_2)_2$, remains.

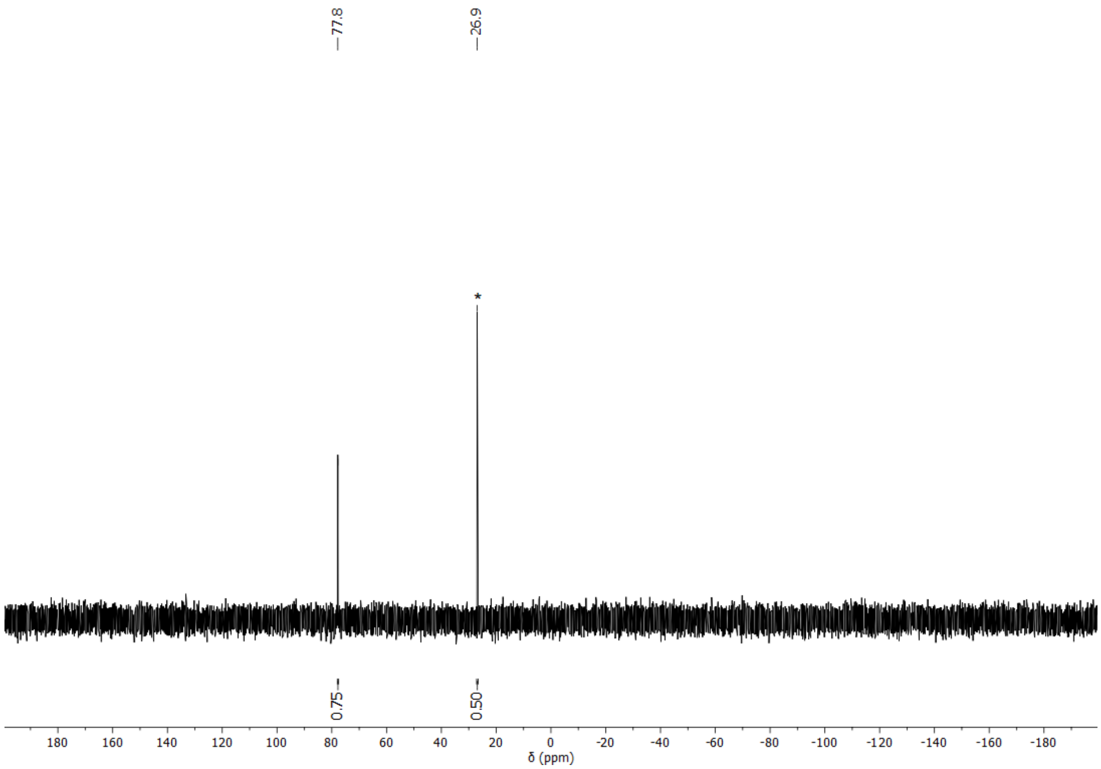

Figure S27. Quantitative single-scan $^{31}P\{^1H\}$ NMR spectrum of tris(*N*-carbazolyl)phosphine generated by the disulfide-mediated functionalisation of P_4 in THF followed by treatment with potassium carbazolide. * = Ph_3PO (internal standard, 0.02 mmol).

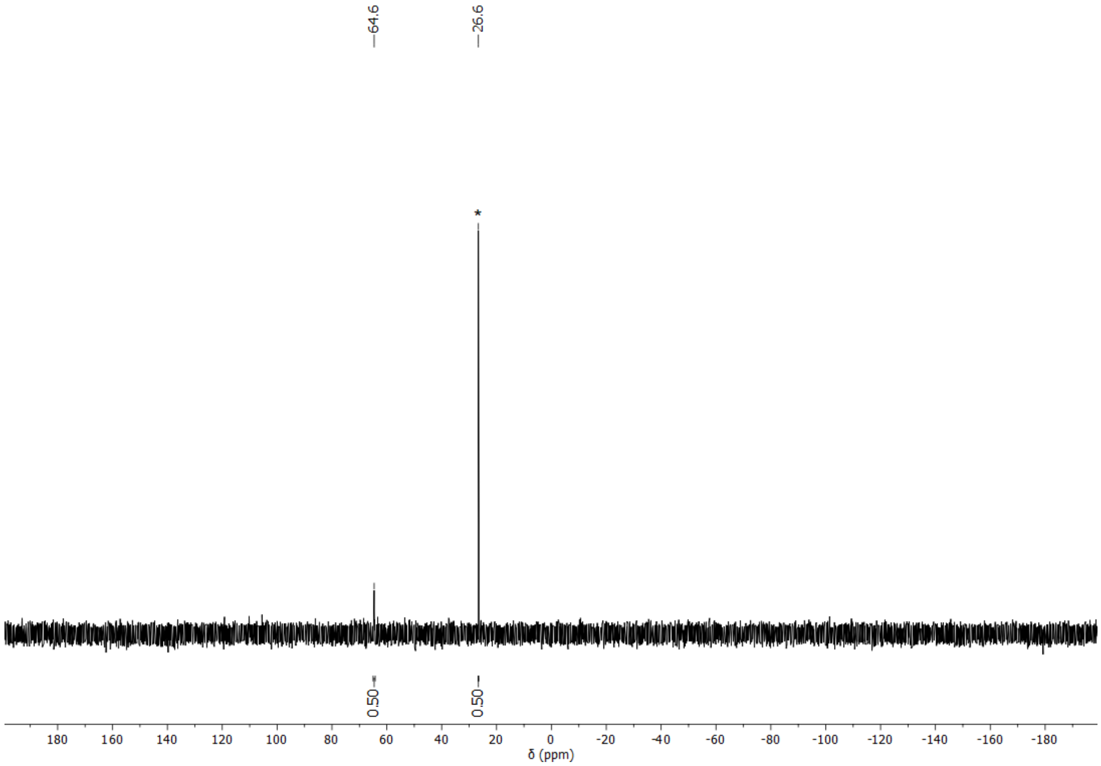

Figure S28. Quantitative single-scan $^{31}P\{^1H\}$ NMR spectrum of tris(3,5-dimethylpyrazolyl)phosphine[50] generated by the disulfide-mediated functionalisation of P_4 in THF followed by treatment with potassium 3,5-dimethylpyrazolide. * = Ph_3PO (internal standard, 0.02 mmol).

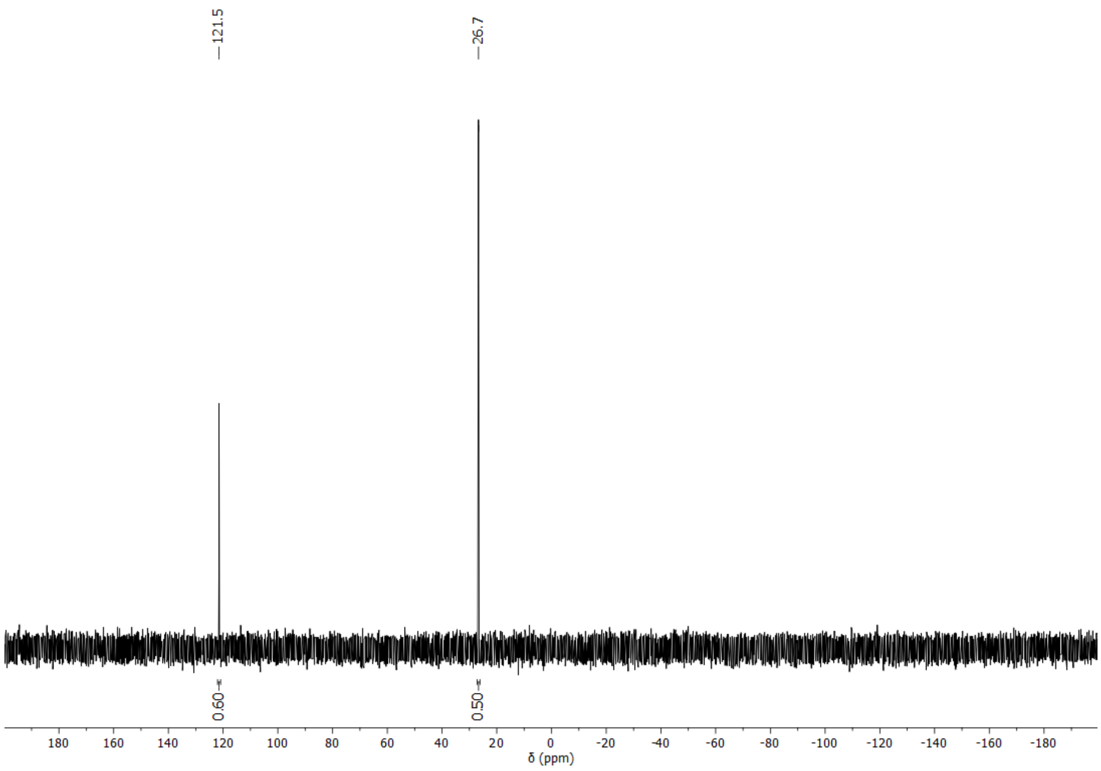

Figure S29. Quantitative single-scan $^{31}P\{^{1}H\}$ NMR spectrum of (4-ClC_6H_4)SP(NPh_2)$_2$[60] generated by the disulfide-mediated functionalisation of P_4 in THF followed by treatment with potassium diphenylamide. * = Ph_3PO (internal standard, 0.02 mmol).

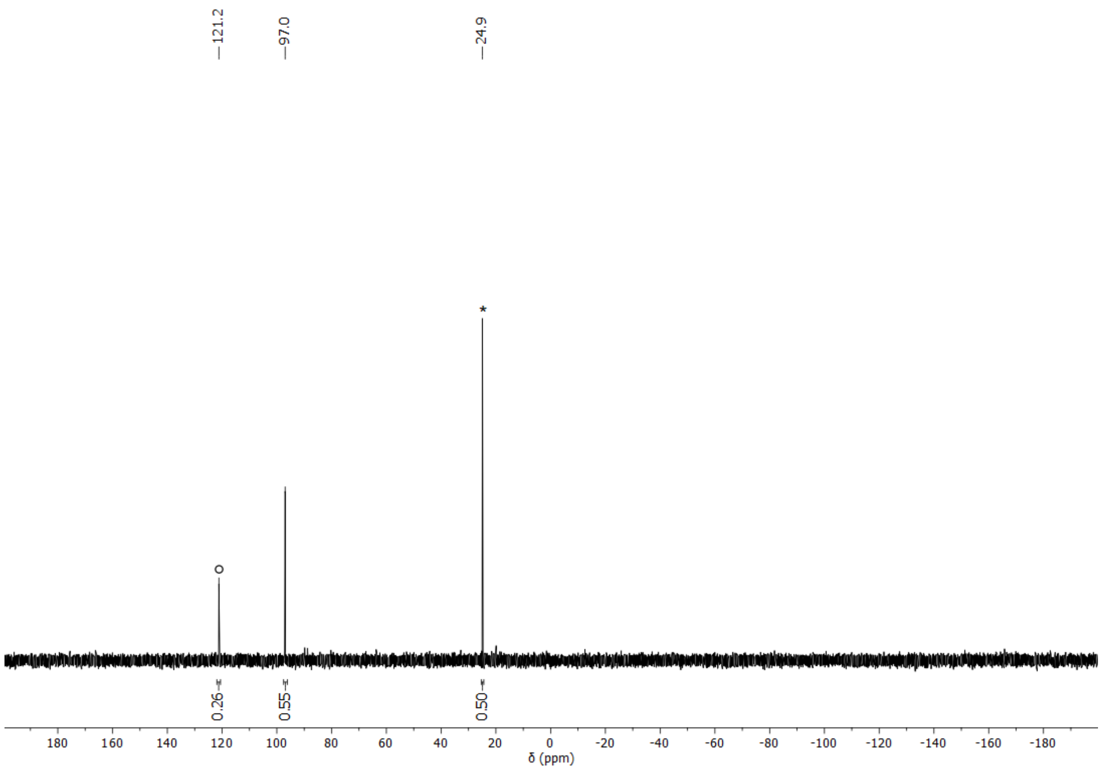

Figure S30. Quantitative single-scan $^{31}P\{^{1}H\}$ NMR spectrum of tris(diphenylamido)phosphine[74] generated by the disulfide-mediated functionalisation of P_4 in THF followed by treatment with potassium diphenylamide. * = Ph_3PO (internal standard, 0.02 mmol); ○ = (4-ClC_6H_4)SP(NPh_2)$_2$.

3.3.3. Synthesis and isolation of $(Ph_2N)_2PS^{Cl}Ar$

To a vial charged with diphenylamine (68 mg, 0.4 mmol, 2 eq. per P atom), and $KN(SiMe_3)_2$ (80 mg, 0.4 mmol, 2 eq. per P atom) was added THF (1 mL), giving a yellow solution. To a separate vial charged with bis(4-chlorophenyl)disulfide (90 mg, 0.31 mmol, 1.57 eq. per P atom) and $KN(SiMe_3)_2$ (1 mg, 0.005 mmol, 0.025 eq. per P atom) was added THF (1 mL), followed by P_4 (0.05 mmol, 500 µL, 0.1 M in C_6H_6). After stirring for 15 min, the potassium diphenylamide solution was added to the trithiophosphite solution, causing rapid precipitation of white solid. After stirring for a further 30 min, volatiles were removed *in vacuo*. The solid residues were extracted with *n*-hexane (1 mL) and filtered. The filtrate was stored at −30 °C overnight, giving colourless crystals of sufficient quality for analysis by X-ray diffraction. The solvent was decanted and the crystals were dried *in vacuo*, giving the target product (32 mg, 0.63 mmol, 31%).

^{1}H NMR (400 MHz, C_6D_6) δ 7.24 – 7.19 (m, 2H), 7.05 – 6.96 (m, 16H), 6.90 – 6.84 ppm (m, 6H).

$^{31}P\{^{1}H\}$ NMR (162 MHz, C_6D_6) δ 120.8 ppm (s).

$^{13}C\{^1H\}$ NMR (101 MHz, C_6D_6) δ 146.6 (d, J_{CP} = 9.0 Hz), 135.8 (d, J = 7.2 Hz), 134.1 (s), 131.4 (d, J_{CP} = 20.4 Hz), 129.5 (s), 129.4 (s), 125.7 (d, J_{CP} = 7.9 Hz), 124.5 ppm (s).

For XRD data, see section 6.

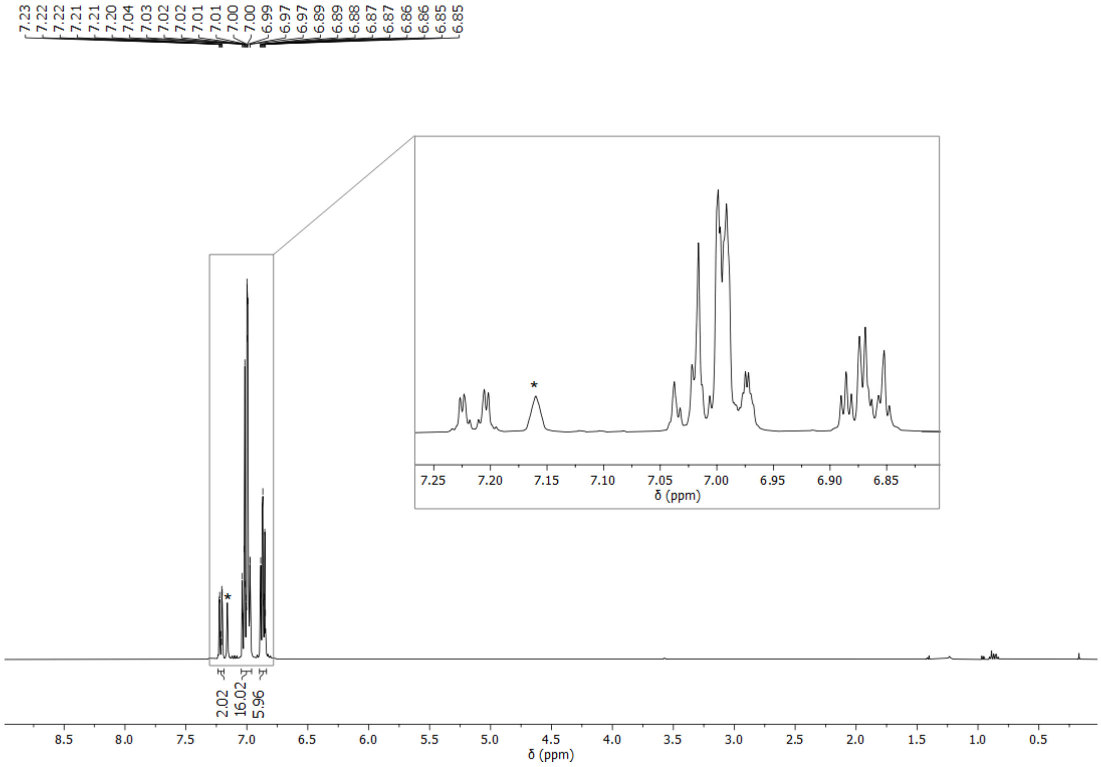

Figure S31. ^1H NMR spectrum of (4-ClC_6H_4)SP(NPh_2)$_2$ in C_6D_6 (*).

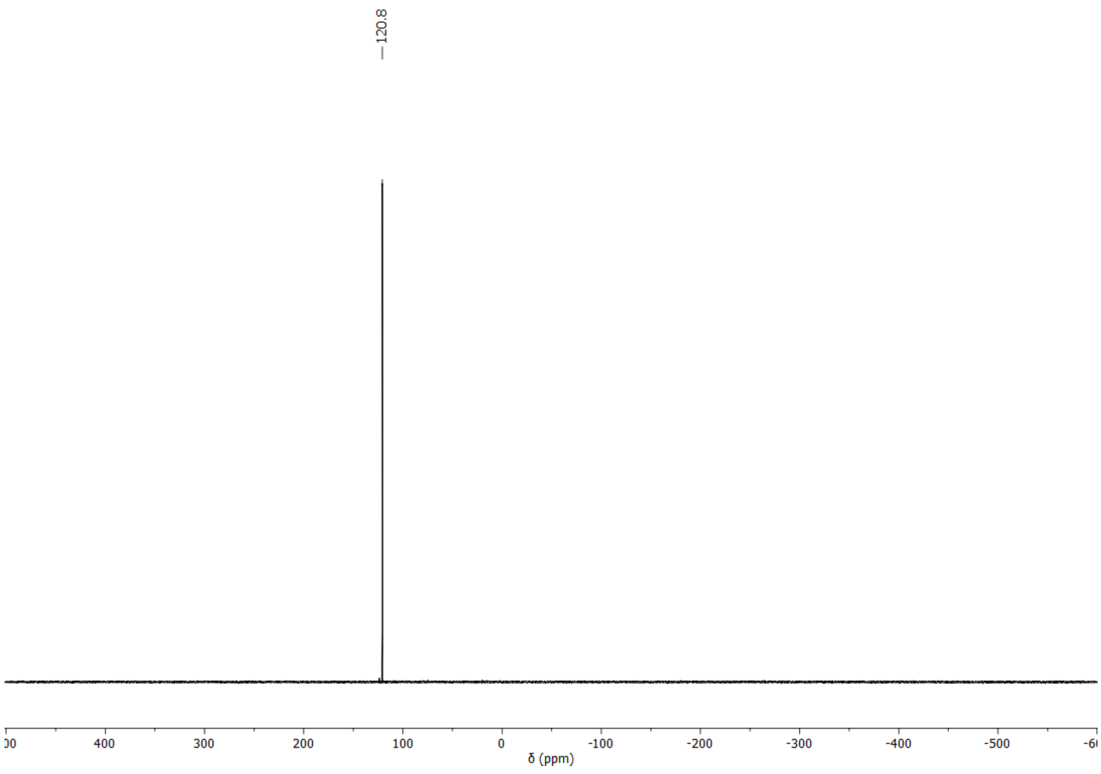

Figure S32. $^{31}P\{^1H\}$ NMR spectrum of (4-ClC_6H_4)SP(NPh_2)$_2$ in C_6D_6.

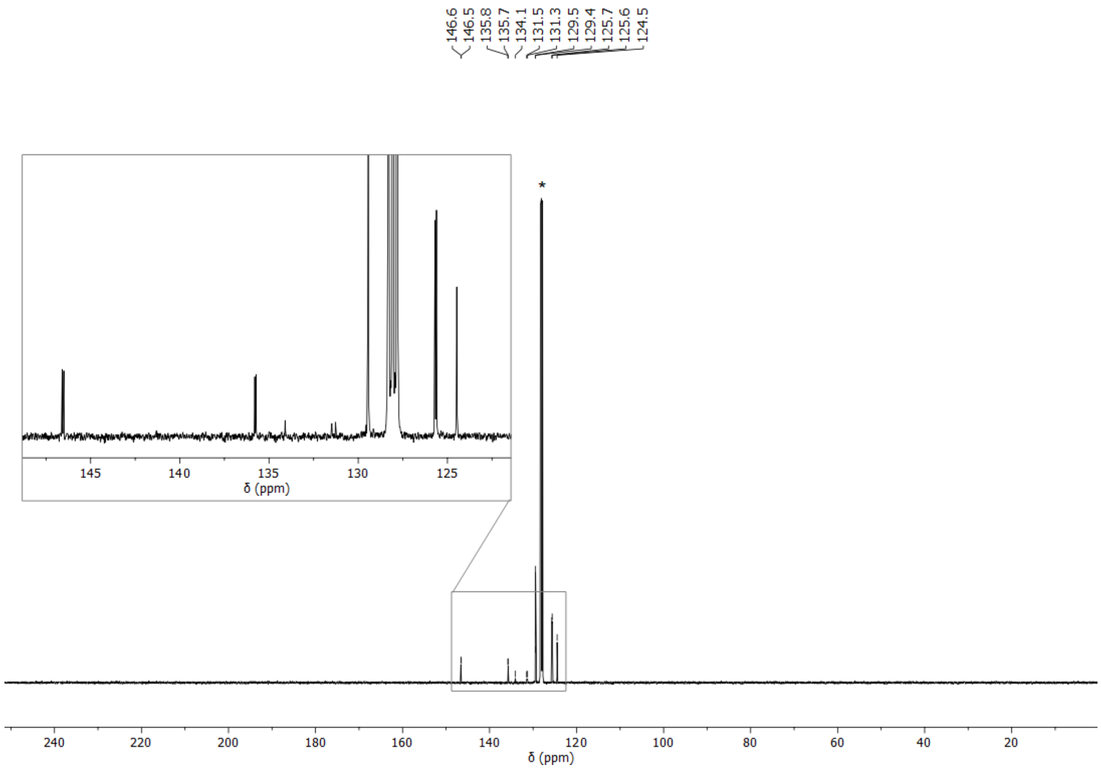

Figure S33. $^{13}C\{^1H\}$ NMR spectrum of (4-ClC_6H_4)SP(NPh_2)$_2$ in C_6D_6 (*).

3.4. One pot synthesis of phosphite esters from P_4 *via* $(ArS)_3P$

3.4.1. Synthesis and isolation of triphenylphosphite

To a suspension of bis(4-chlorophenyl)disulfide (0.75 g, 2.6 mmol, 1.63 eq. per P atom) and $KN(SiMe_3)_2$ (7 mg, 0.04 mmol, 0.025 eq. per P atom) in MeCN (25 mL) was added a solution of P_4 (50 mg, 0.4 mmol) in toluene (2 mL). After stirring for 1 h, a solution of phenol (0.90 g, 9.6 mmol, 6 eq. per P atom) in MeCN (10 mL) was added. The resulting colourless solution was stirred overnight. Volatiles were removed *in vacuo*, giving a colourless liquid. The thiol byproduct and excess phenol were removed by sublimation *in vacuo*, and triphenyl phosphite (291 mg, 0.94 mmol, 59%) was isolated as a viscous, colourless oil by distillation at 130 °C *in vacuo*.

^{1}H NMR (400 MHz, C_6D_6) δ 7.19 – 7.12 (m, 6H, overlapping solvent resonance), 7.03 – 6.96 (m, 6H), 6.87 – 6.80 ppm (m, 3H).

^{31}P{^{1}H} NMR (162 MHz, C_6D_6) δ 128.6 ppm (s).

^{13}C{^{1}H} NMR (101 MHz, C_6D_6) δ 152.3 (d, J_{CP} = 3.1 Hz), 130.0 (s), 124.4 (s), 121.2 ppm (d, J_{CP} = 6.9 Hz).

These NMR data are commensurate with previous literature reports.[51]

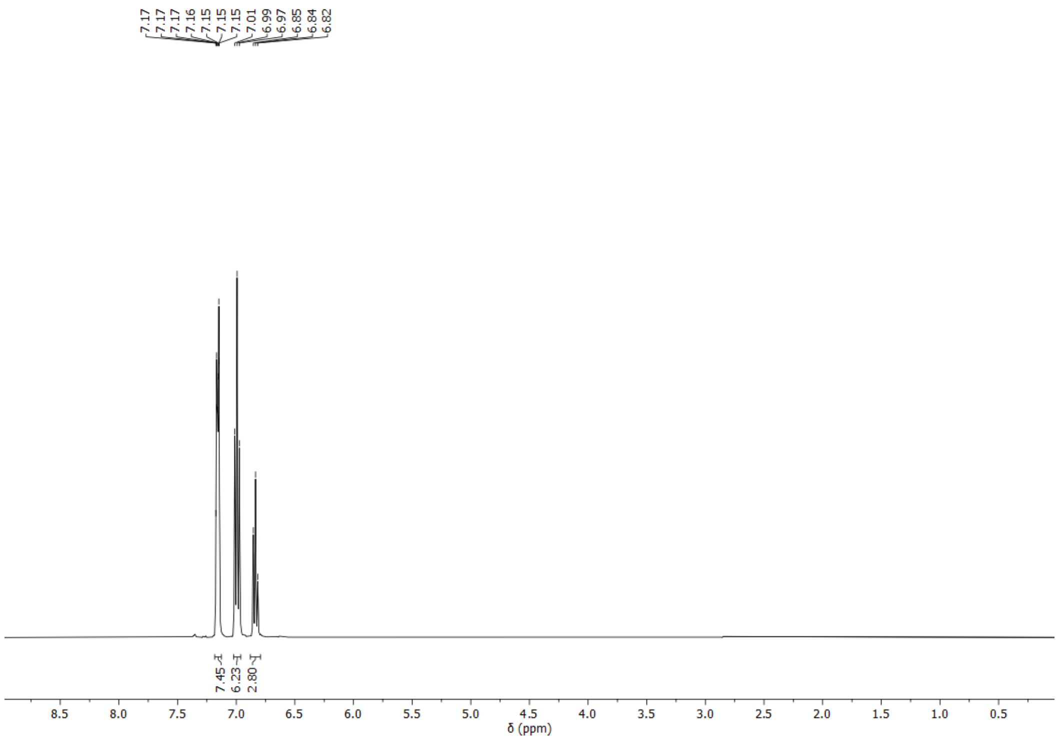

Figure S34. ^{1}H NMR spectrum of triphenylphosphite in C_6D_6.

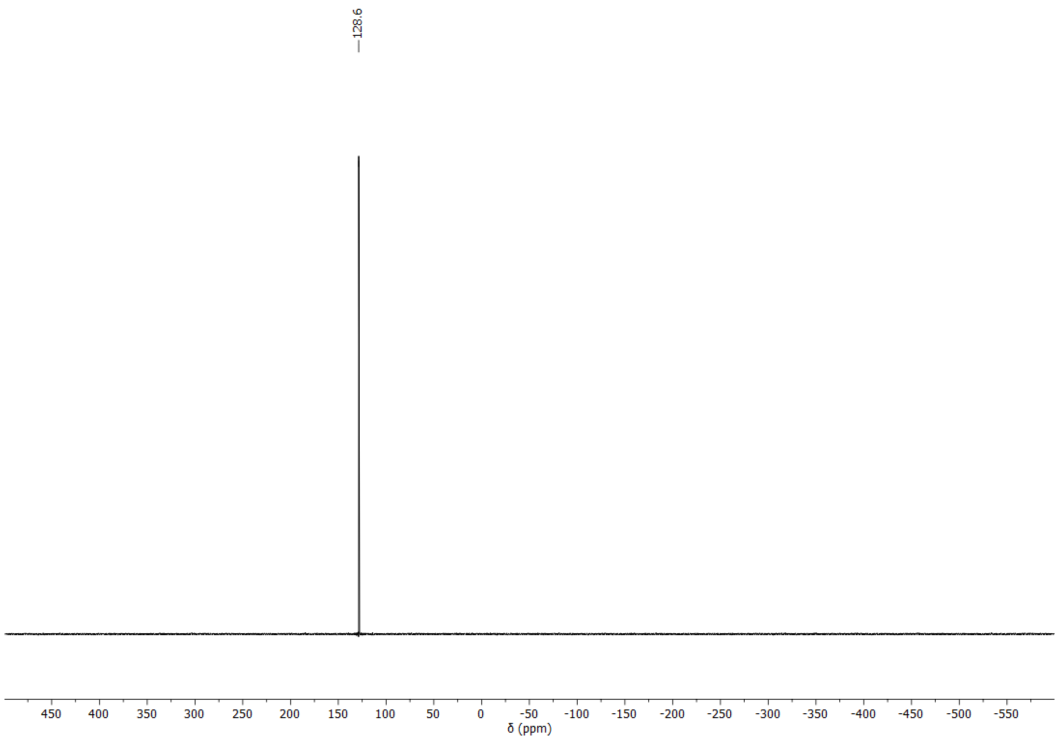

Figure S35. $^{31}P\{^{1}H\}$ NMR spectrum of triphenylphosphite in C_6D_6.

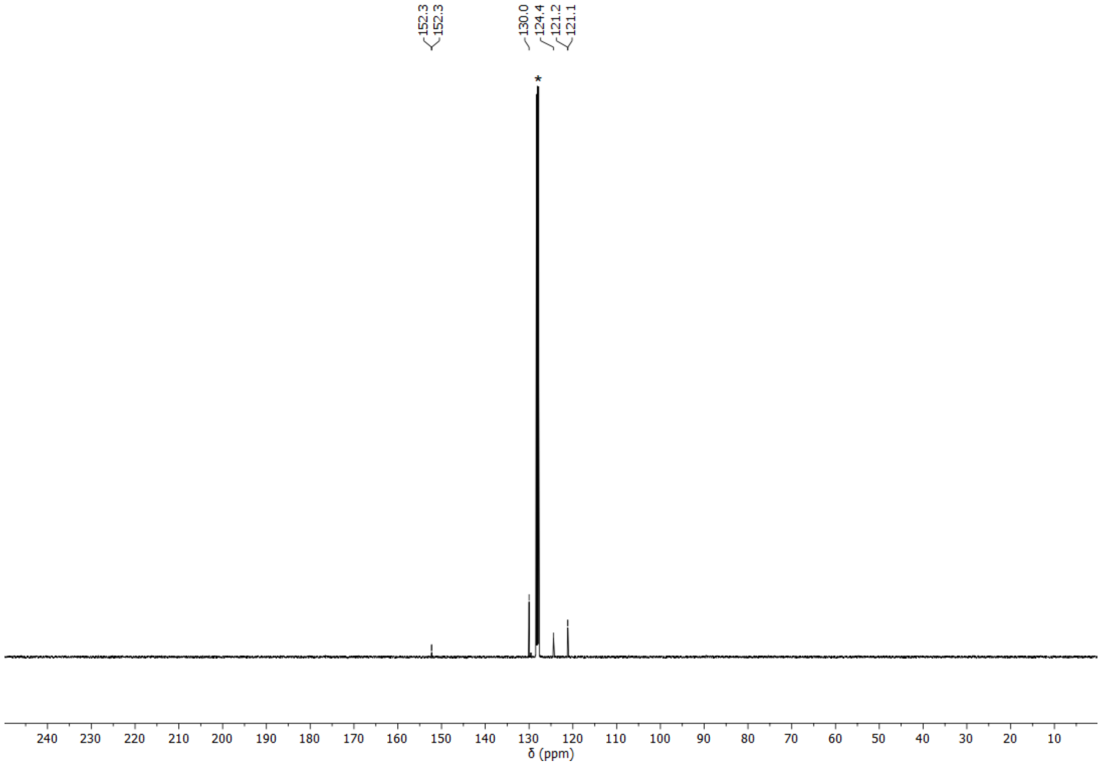

Figure S36. $^{13}C\{^{1}H\}$ NMR spectrum of triphenylphosphite in C_6D_6 (*).

3.4.2. Generation of phosphite esters from P_4 and *in situ* measurement of conversion

To a solution of bis(4-chlorophenyl)disulfide (0.065 mmol, 18.7 mg, 1.63 eq. per P atom) and $KN(SiMe_3)_2$ (0.001 mmol, 0.2 mg, 0.025 eq. per P atom) in MeCN (0.5 mL) was added P_4 (0.01 mmol, 100 μL, 0.1 M in C_6H_6), to generate the thiophosphite intermediate tris(4-chlorophenyl)thiophosphite. Following *ca*. 1 h of stirring, the corresponding phenol (6 eq. per P atom) was added. The reaction was left stirring overnight, then an internal standard of Ph_3PO (0.02 mmol, 250 μL, 0.08 M in MeCN) was added. The reaction was transferred to an NMR tube equipped with a C_6D_6 capillary, and submitted for $^{31}P\{^1H\}$ NMR spectroscopy. Conversions are summarized in Table S4.

Table S4. Conversion to $(ArO)_3P$ measured for disulfide-mediated reactions between P_4 and phenols ArOH.

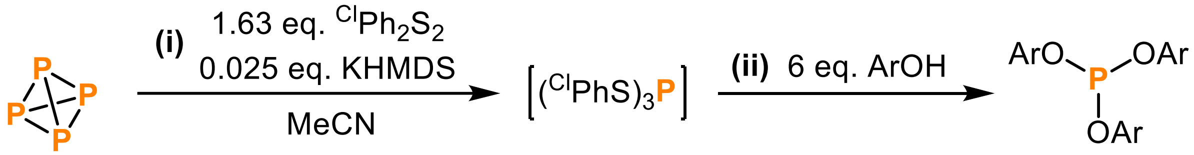

Entry	Ar	Conv. to $(ArO)_3P$ / %
1	Ar = phenyl	52*
2	Ar = 4-methylphenyl	76
3	Ar = 3-methylphenyl	48
4	Ar = 2-methylphenyl	58
5	Ar = 3-chlorophenyl	24
6	Ar = 4-chlorophenyl	33
7	Ar = 4-fluorophenyl	51
8	Ar = 4-(methylmercapto)phenyl	51
9	Ar = 4-methoxyphenyl	85
10	Ar = 3,5-dimethoxyphenyl	42

* 3 day reaction time after addition of PhOH

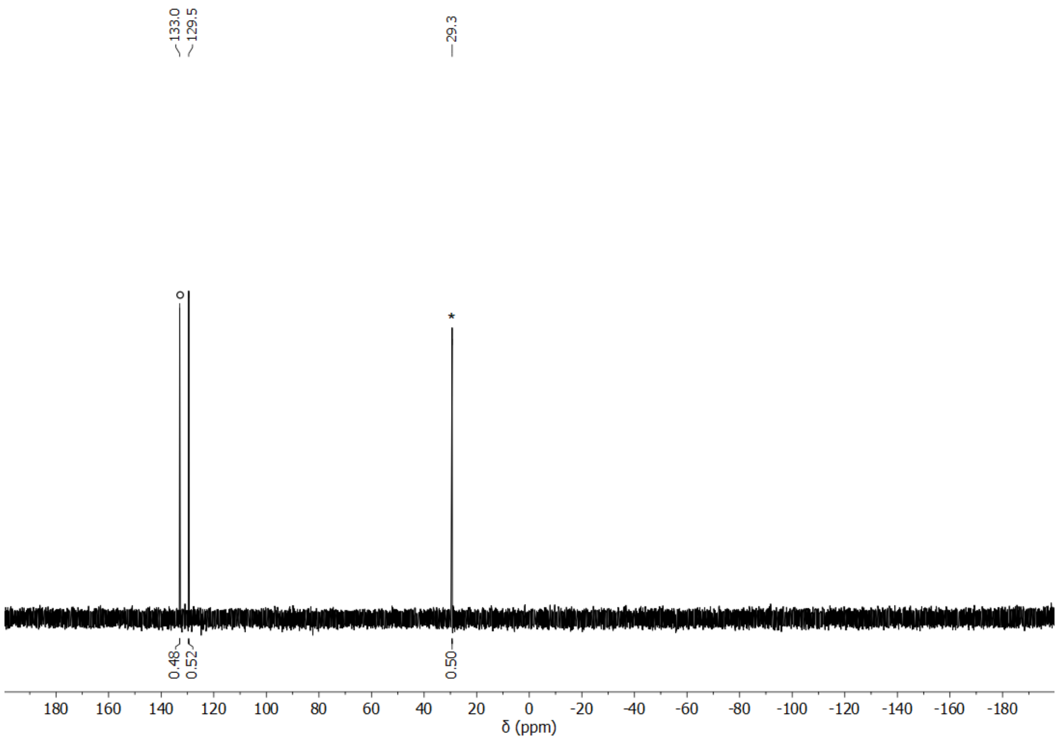

Figure S37. Quantitative ${}^{31}P\{{}^{1}H\}$ NMR spectrum of triphenylphosphite generated by the disulfide-mediated functionalisation of P_4 in MeCN followed by treatment with phenol. * = Ph_3PO (internal standard, 0.02 mmol), ○ = tris(4-chlorophenyl)thiophosphite.

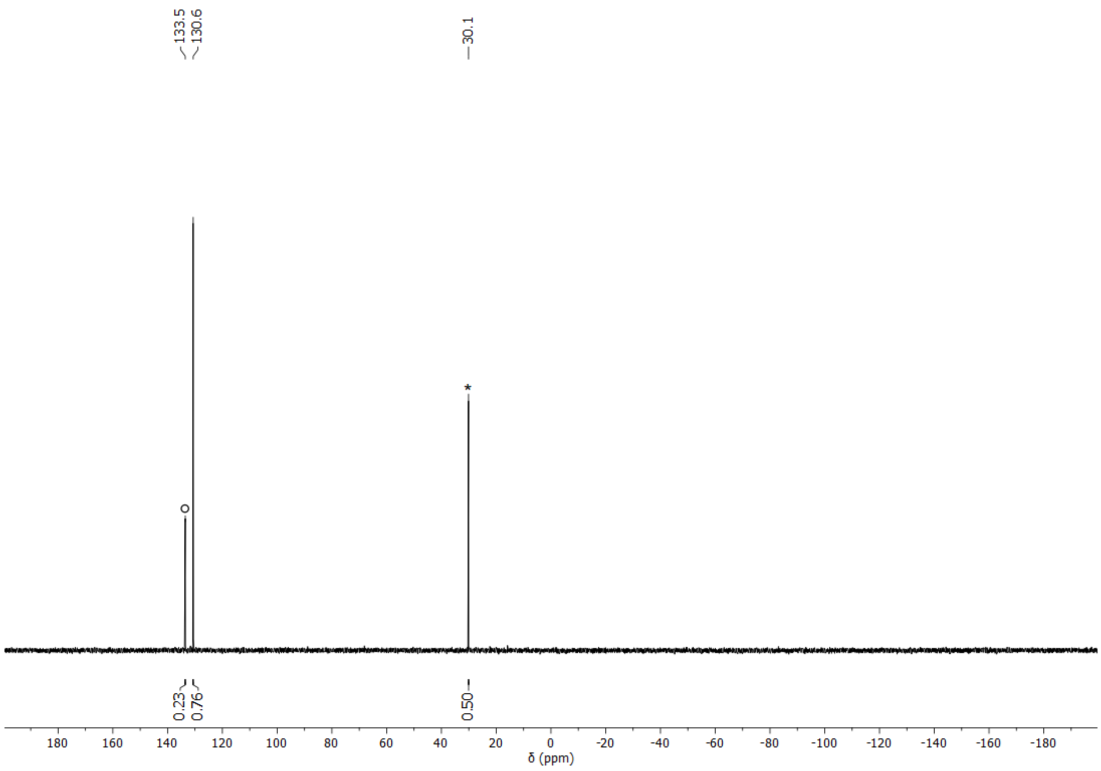

Figure S38. Quantitative single-scan $^{31}P\{^1H\}$ NMR spectrum of tris(4-methylphenyl)phosphite generated by the disulfide-mediated functionalisation of P_4 in MeCN followed by treatment with *p*-cresol. * = Ph_3PO (internal standard, 0.02 mmol), ○ = tris(4-chlorophenyl)thiophosphite.

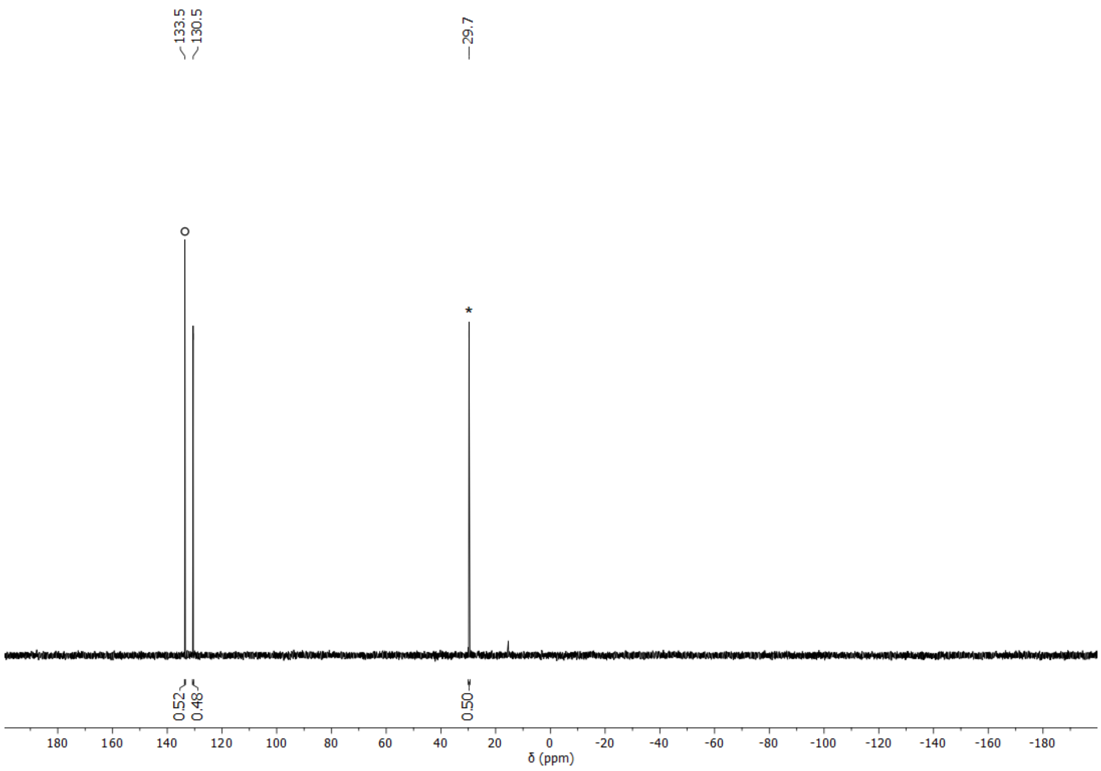

Figure S39. Quantitative single-scan $^{31}P\{^{1}H\}$ NMR spectrum of tris(3-methylphenyl)phosphite generated by the disulfide-mediated functionalisation of P_4 in MeCN followed by treatment with *m*-cresol. * = Ph_3PO (internal standard, 0.02 mmol), ○ = tris(4-chlorophenyl)thiophosphite.

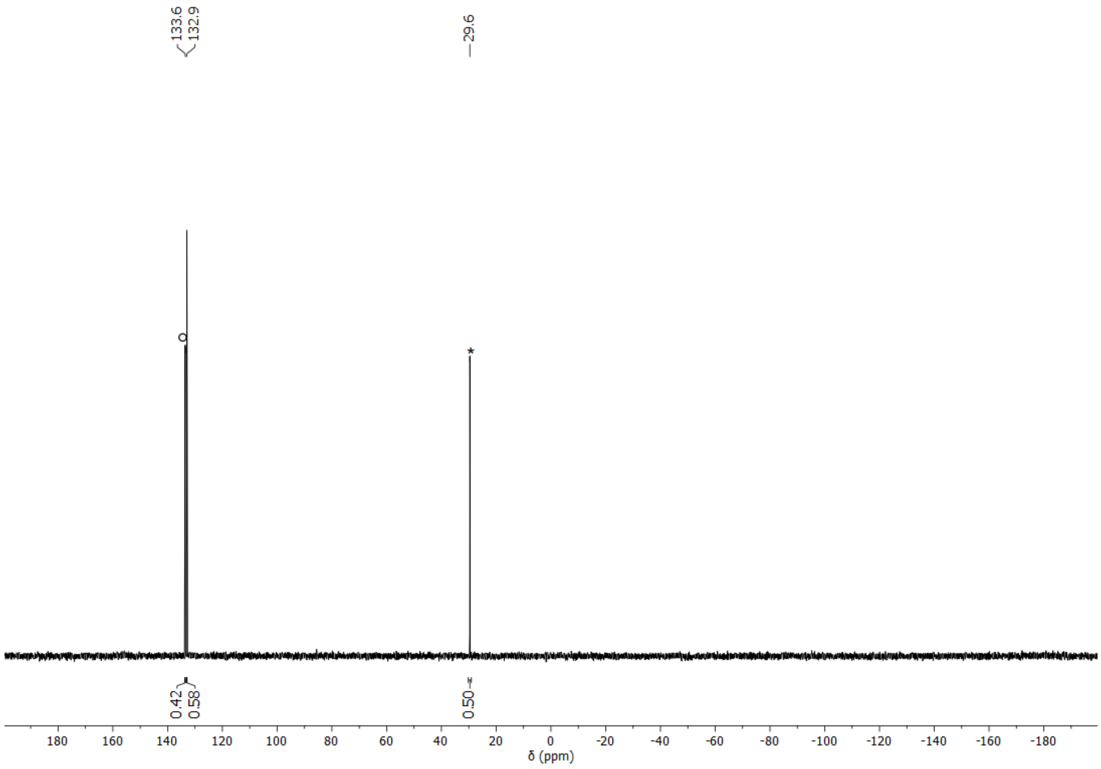

Figure S40. Quantitative single-scan $^{31}P\{^1H\}$ NMR spectrum of tris(2-methylphenyl)phosphite generated by the disulfide-mediated functionalisation of P_4 in MeCN followed by treatment with *o*-cresol. * = Ph_3PO (internal standard, 0.02 mmol), ○ = tris(4-chlorophenyl)thiophosphite.

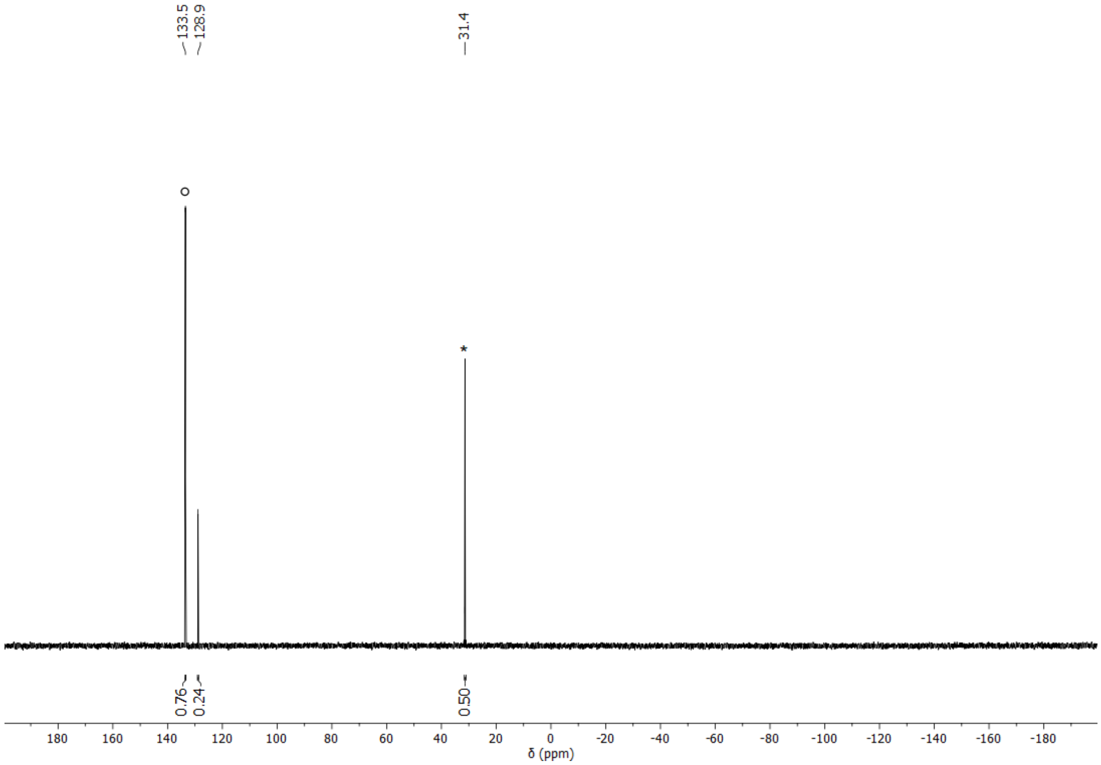

Figure S41. Quantitative single-scan $^{31}P\{^{1}H\}$ NMR spectrum of tris(3-chlorophenyl)phosphite generated by the disulfide-mediated functionalisation of P_4 in MeCN followed by treatment with 3-chlorophenol. * = Ph_3PO (internal standard, 0.02 mmol), ○ = tris(4-chlorophenyl)thiophosphite.

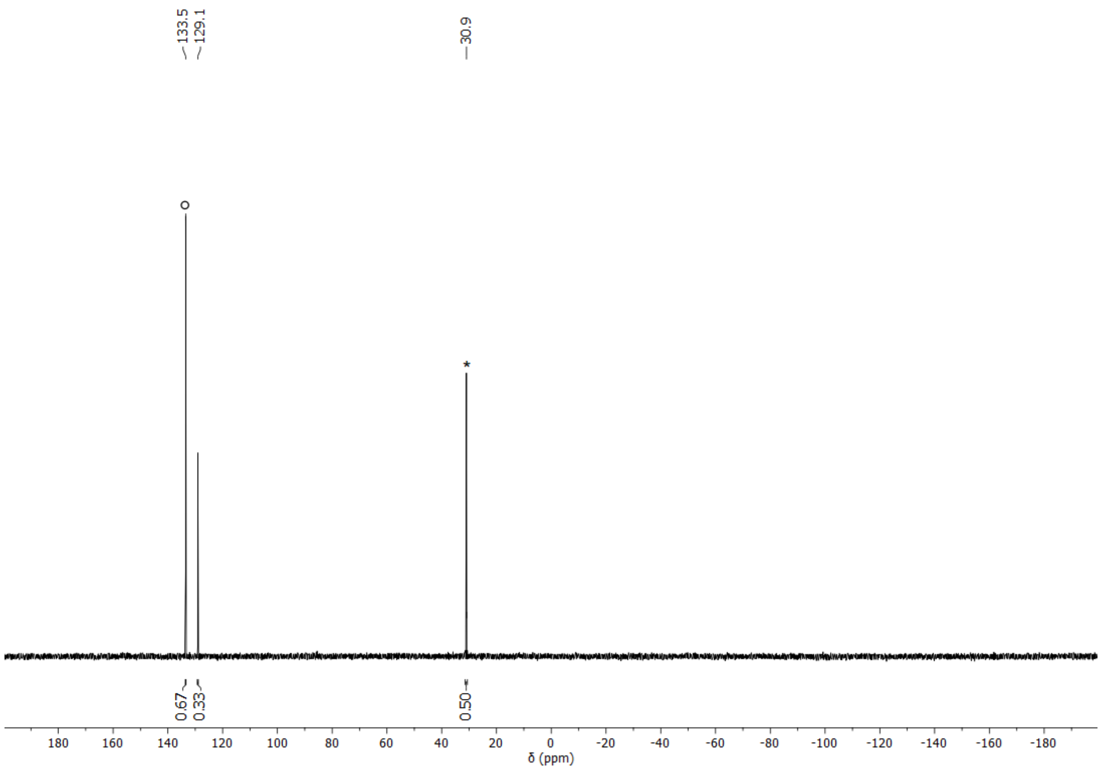

Figure S42. Quantitative single-scan $^{31}P\{^{1}H\}$ NMR spectrum of tris(4-chlorophenyl)phosphite generated by the disulfide-mediated functionalisation of P_4 in MeCN followed by treatment with 4-chlorophenol. * = Ph_3PO (internal standard, 0.02 mmol), ○ = tris(4-chlorophenyl)thiophosphite.

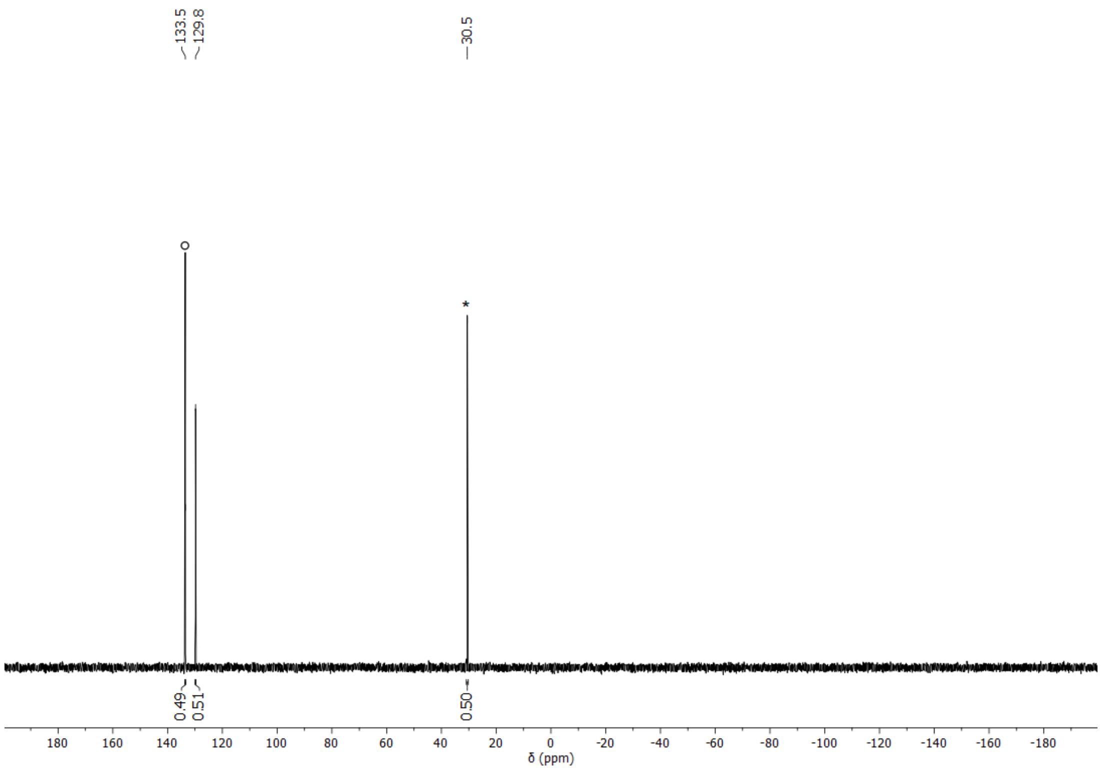

Figure S43. Quantitative single-scan $^{31}P\{^{1}H\}$ NMR spectrum of tris(4-fluorophenyl)phosphite generated by the disulfide-mediated functionalisation of P_4 in MeCN followed by treatment with 4-fluorophenol. * = Ph_3PO (internal standard, 0.02 mmol), ○ = tris(4-chlorophenyl)thiophosphite.

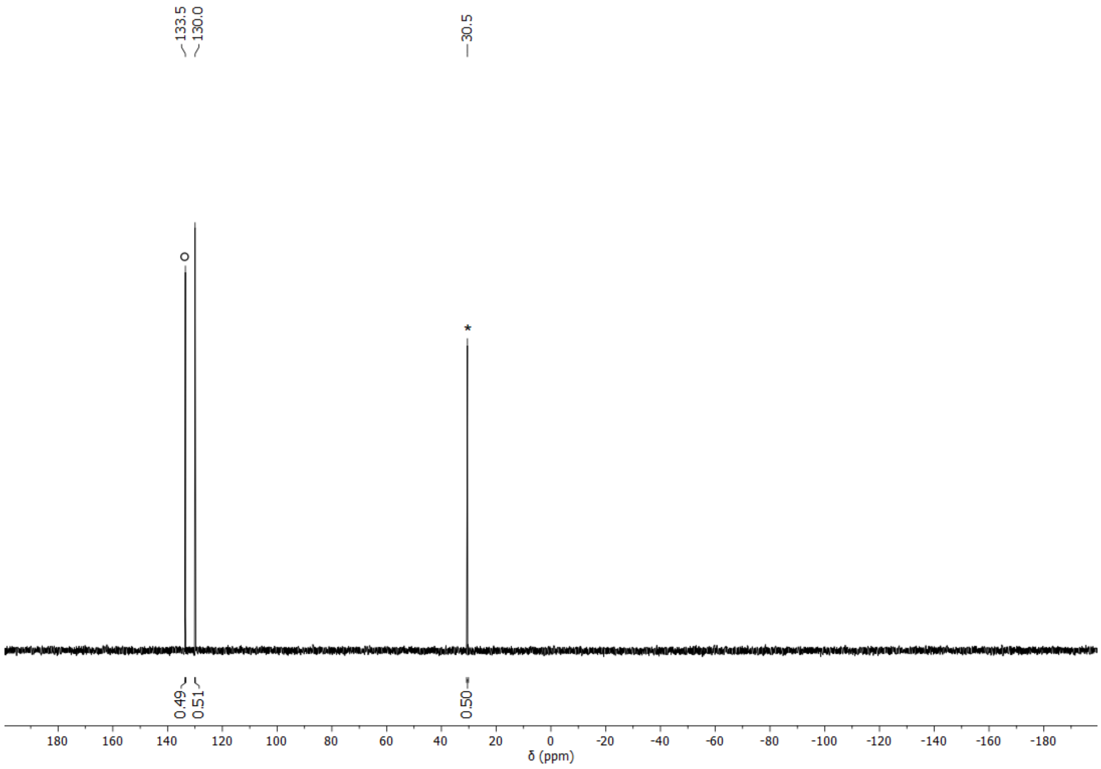

Figure S44. Quantitative single-scan $^{31}P\{^{1}H\}$ NMR spectrum of tris(4-methylmercaptophenyl)phosphite generated by the disulfide-mediated functionalisation of P_4 in MeCN followed by treatment with 4-(methylmercapto)phenol. * = Ph_3PO (internal standard, 0.02 mmol), ○ = tris(4-chlorophenyl)thiophosphite.

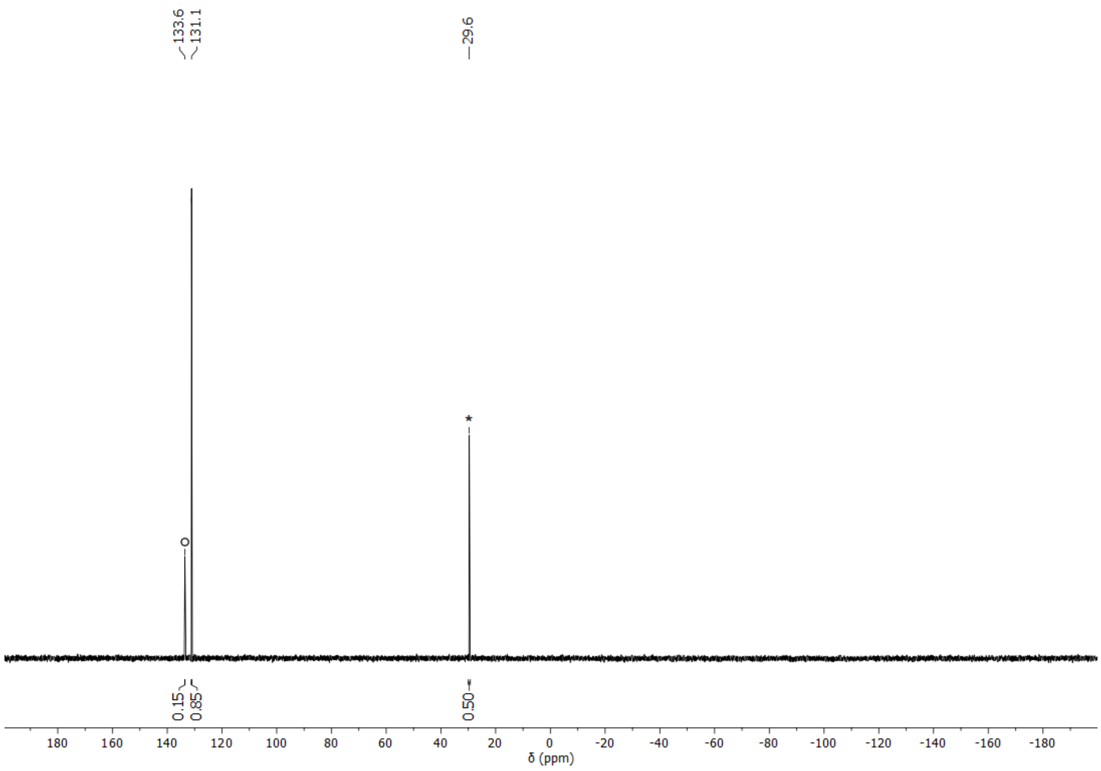

Figure S45. Quantitative single-scan $^{31}P\{^{1}H\}$ NMR spectrum of tris(4-methoxyphenyl)phosphite generated by the disulfide-mediated functionalisation of P_4 in MeCN followed by treatment with 4-methoxyphenol. * = Ph_3PO (internal standard, 0.02 mmol), ○ = tris(4-chlorophenyl)thiophosphite.

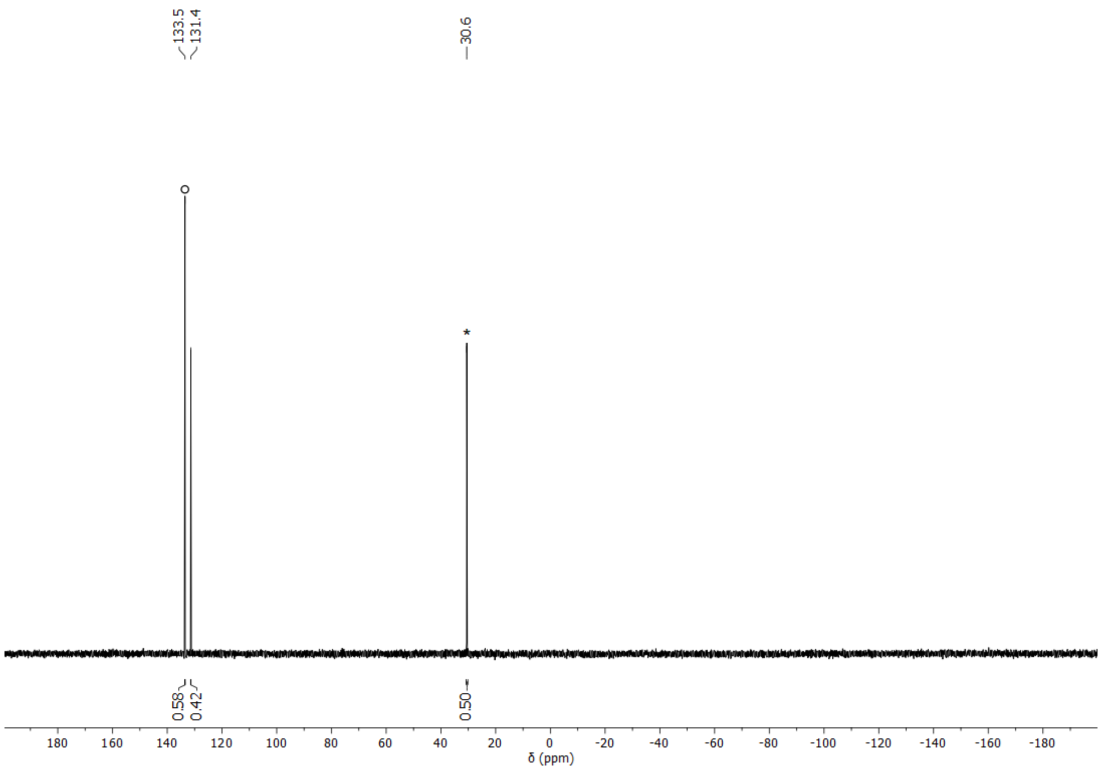

Figure S46. Quantitative single-scan $^{31}P\{^1H\}$ NMR spectrum of tris(3,5-dimethoxyphenyl)phosphite generated by the disulfide-mediated functionalisation of P_4 in MeCN followed by treatment with 3,5-dimethoxyphenol. * = Ph_3PO (internal standard, 0.02 mmol), ○ = tris(4-chlorophenyl)thiophosphite.

3.4.3. Generation of phosphite ester from P_{red} and *in situ* measurement of conversion

In a glovebox, a photoreactor tube was charged with a stirrer bar, bis(4-chlorophenyl)disulfide (46.0 mg, 0.16 mmol, 1.6 eq.), $KN(SiMe_3)_2$ (2.0 mg, 0.01 mmol, 0.1 eq.) and red phosphorus (3.1 mg, 0.1 mmol). MeCN (0.6 mL) was added, and the resulting suspension was removed from the glovebox and heated to 80 °C. After stirring for 7 days at this temperature, the reaction was brought into the glove box. The reaction mixture was filtered and transferred to an NMR tube equipped with a C_6D_6 capillary. *p*-Cresol (63 µL, 0.6 mmol, 6 eq.) and an internal standard of Ph_3PO (0.02 mmol, 250 µL, 0.08 M in MeCN) were added, and the solution was submitted for quantitative $^{31}P\{^1H\}$ NMR spectroscopy. The resulting spectrum is shown in Figure S47, below.

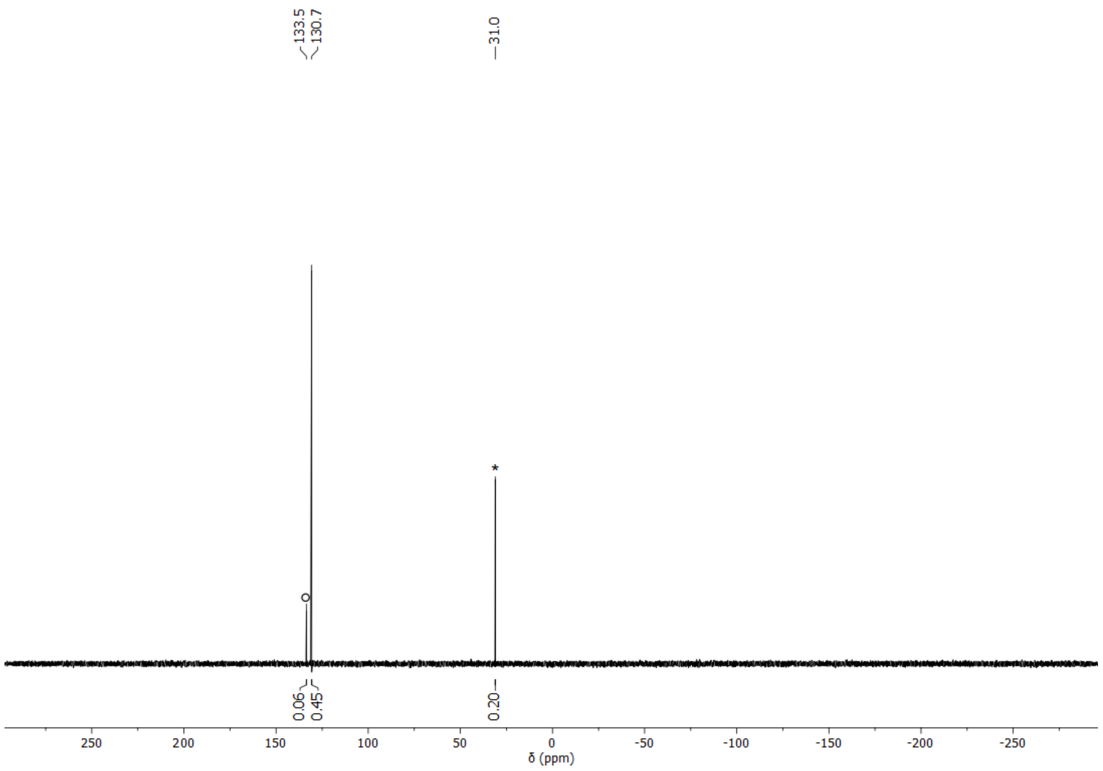

Figure S47. Quantitative single-scan $^{31}P\{^{1}H\}$ NMR spectrum of tris(4-methylphenyl)phosphite generated by the disulfide-mediated functionalisation of P_{red} in MeCN followed by treatment with *p*-cresol. * = Ph_3PO (internal standard, 0.02 mmol), ○ = tris(4-chlorophenyl)thiophosphite.

4. Catalytic reaction development

4.1. Initial reaction development

4.1.1. Generation of $(TolS)_3P$ from P_4 and TolSH

To begin the development of a catalytic procedure, the generation of $(ArS)_3P$ from the corresponding thiol (ArSH) and P_4 in the presence of an oxidant was targeted as a necessary first step. For this purpose, Ar = Tol (4-tolyl) was chosen as a simple model system and Et_3N was chosen as a simple base for its deprotonation. $Na_2S_2O_8$ was identified as a plausible oxidant since it is inexpensive and readily available, does not react directly with P_4 (as indicated by independent control experiments), and the peroxydisulfate anion is known to be capable of oxidizing thiols to disulfides.[67]

Thus:

To a suspension of TolSH (0.13 mmol, 16.1 mg, 3.25 eq. per P atom) and $Na_2S_2O_8$ (0.06 mmol, 14.3 mg, 1.5 eq. per P atom) in MeCN (0.5 mL) was added Et_3N (0.13 mmol, 18.1 µL, 3.25 eq. per P atom), followed by P_4 (0.01 mmol, 97.6 µL, 0.1025 M in C_6H_6), resulting in a colourless suspension which quickly became yellow and cloudy.

The resulting reaction mixture was analysed by $^{31}P\{^{1}H\}$ NMR spectroscopy, and the formation of $(TolS)_3P$ was indicated by the expected resonance at 134.5 ppm (Figure S48), confirming the viability of the desired transformation.[55]

The reaction was then repeated with addition of Ph_3PO (23.0 mg, 0.083 mmol) as an internal standard at the end of the reaction. Quantification of $(TolS)_3P$ indicated 82% conversion to this product under these conditions (Figure S49).

The reaction was also repeated in the absence of the $Na_2S_2O_8$ oxidant, which as expected showed only trace formation of $(TolS)_3P$ (Figure S50).

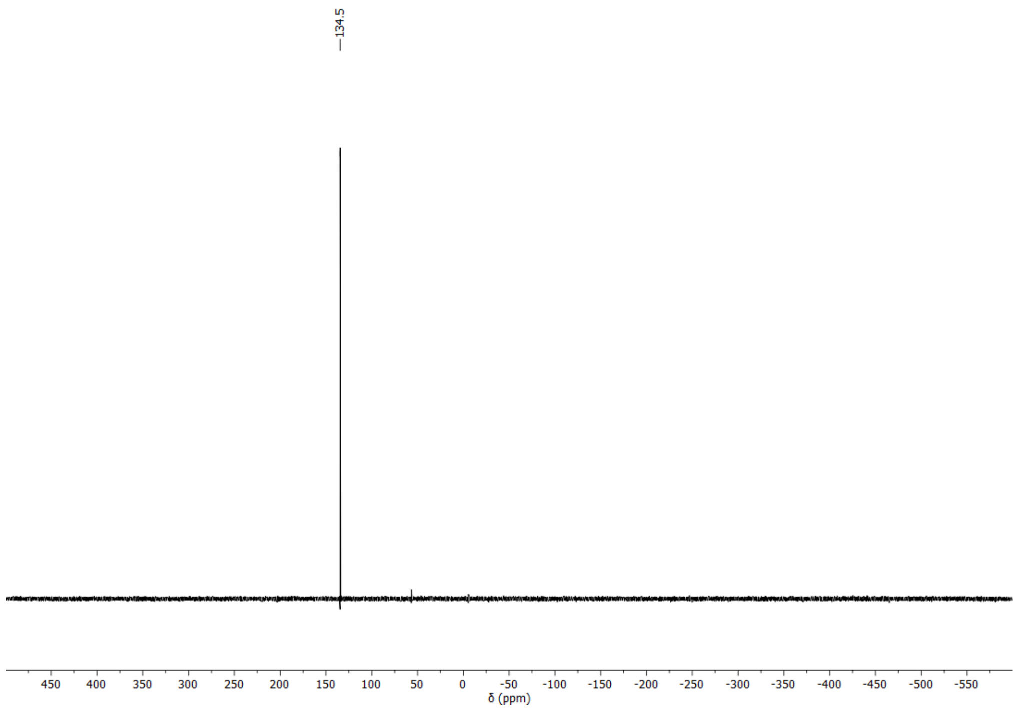

Figure S48. $^{31}P\{^{1}H\}$ NMR spectrum of the reaction between P_4, TolSH (3.25 eq.), Et_3N (3.25 eq.) and $Na_2S_2O_8$ (1.5 eq.) in MeCN. All eq. defined per P atom.

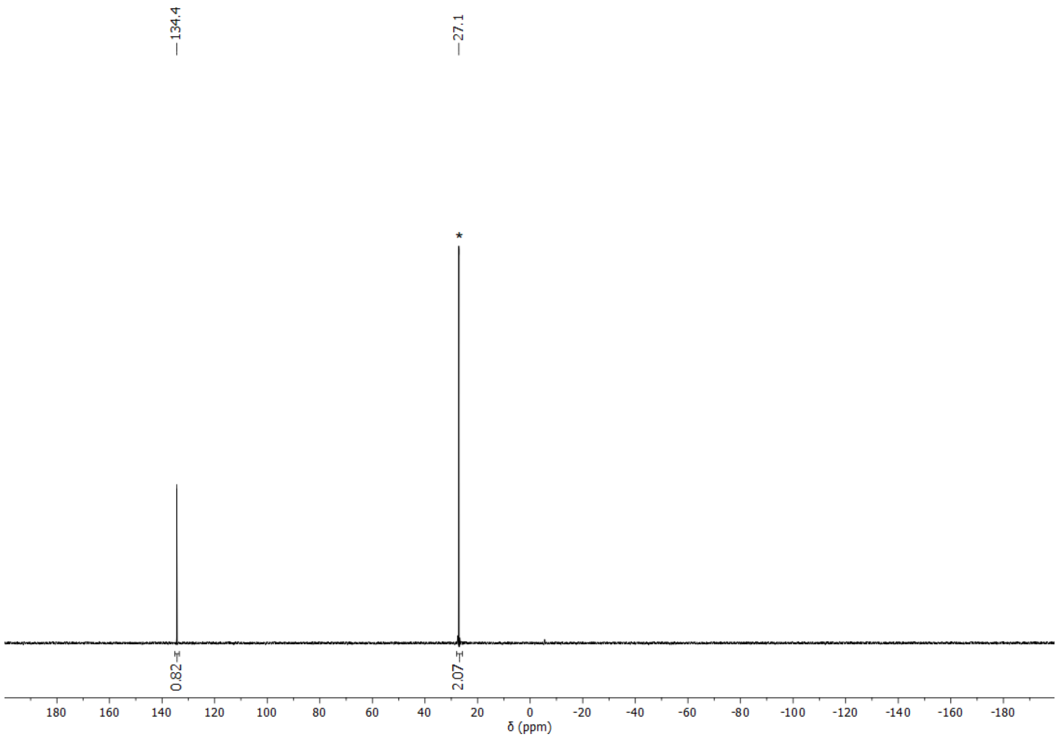

Figure S49. Quantitative single-scan $^{31}P\{^{1}H\}$ NMR spectrum of the reaction between P_4, TolSH (3.25 eq.), Et_3N (3.25 eq.) and $Na_2S_2O_8$ (1.63 eq.) in MeCN, following addition of 0.083 mmol Ph_3PO (*) as an internal standard. All eq. defined per P atom.

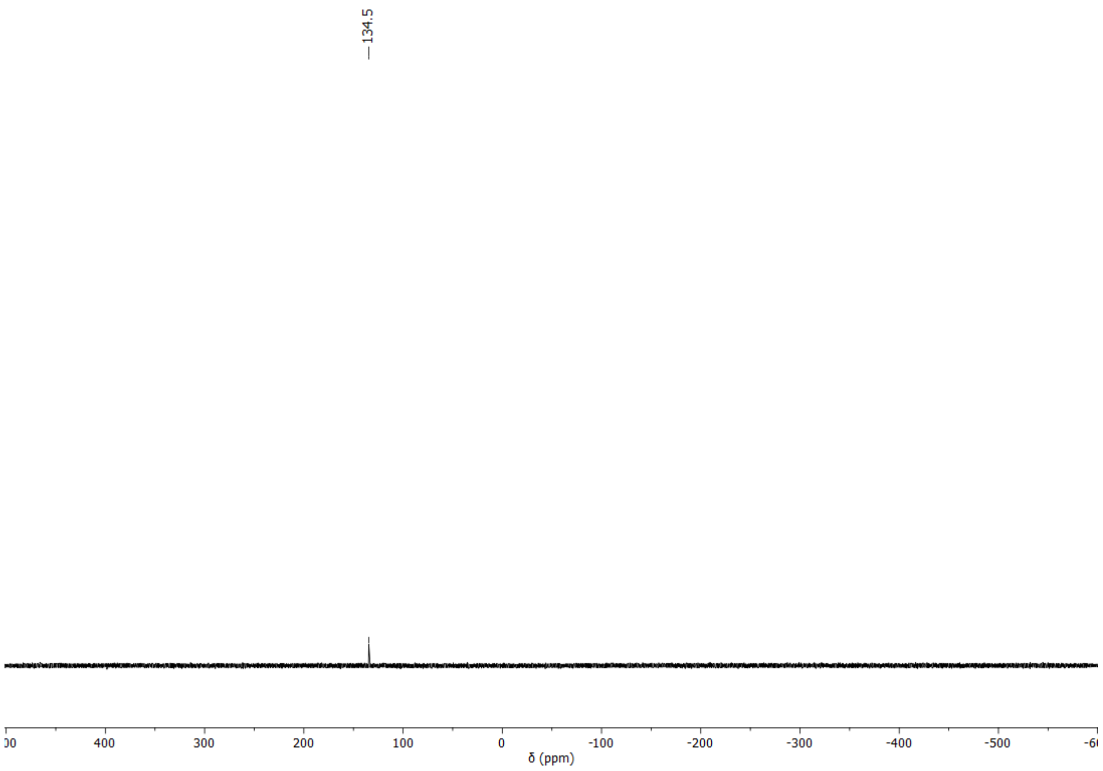

Figure S50. $^{31}P\{^{1}H\}$ NMR spectrum of the reaction between P_4, TolSH (3.25 eq.) and Et_3N (3.25 eq.) and MeCN in the absence of $Na_2S_2O_8$. All eq. defined per P atom.

4.1.2. Generation of (TolS)$_3$P from P_4 and TolSH in the presence of PhOH

Following successful demonstration of (TolS)$_3$P generation from TolSH, P_4 and $Na_2S_2O_8$, the reaction was repeated in the presence of PhOH as a model nucleophile (additional Et_3N was also employed to facilitate PhOH deprotonation).

Thus:

To a suspension of TolSH (0.13 mmol, 16.1 mg, 3.25 eq. per P atom), $Na_2S_2O_8$ (0.065 mmol, 15.5 mg, 1.63 eq. per P atom) and PhOH (0.13 mmol, 12.2 mg, 3.25 eq. per P atom) in MeCN (0.5 mL) was added Et_3N (0.13 mmol, 36.2 µL, 6.5 eq. per P atom), followed by P_4 (0.01 mmol, 100 µL, 0.1 M in C_6H_6), resulting in a colourless suspension which quickly became yellow and cloudy.

The resulting reaction mixture was analysed by $^{31}P\{^{1}H\}$ NMR spectroscopy, which showed formation of (TolS)$_3$P very similarly to in the absence of PhOH (Figure S51; *cf.* section 4.1.1). However, this was accompanied by formation of a minor peak at 130.2 ppm which can be assigned to (PhO)$_3$P (confirmed by

spiking the NMR sample with authentic $(PhO)_3P$, see Figure S51b). The results clearly indicate that the oxidative transformation of TolSH and P_4 into $(TolS)_3P$ is compatible with the presence of PhOH, and also indicate that substitution of the TolS groups is feasible. The relatively small amount of $(PhO)_3P$ formed in comparison to the optimised catalytic reaction is tentatively attributed to the much larger amount of "TolS" present as well as the relatively electron-rich TolS moiety which create an equilibrium between $(PhO)_3P$ and $(TolS)_3P$ that favours the latter. Interestingly, no spectroscopic evidence of 'mixed' $(TolS)_2POPh$ or $TolSP(OPh)_2$ was observed (the reasons for which have not been explored).

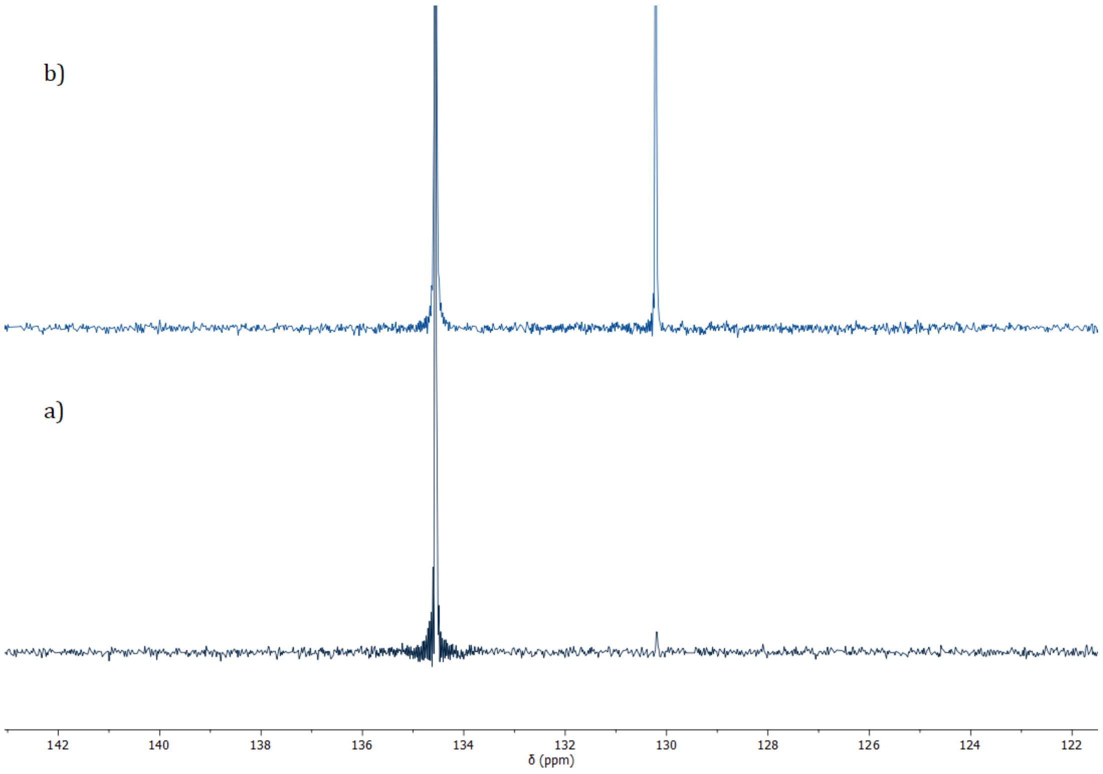

Figure S51. $^{31}P\{^{1}H\}$ NMR spectra of a) the reaction between P_4, TolSH (3.25 eq.), Et_3N (6.5 eq.) and $Na_2S_2O_8$ (1.63 eq). in MeCN in the presence of PhOH (3.25 eq.) and b) the same reaction following the addition of authentic $(PhO)_3P$. All eq. defined per P atom.

4.1.3. Optimisation of the catalytic synthesis of $(PhO)_3P$ from P_4 and PhOH

Optimisation of catalyst structure

For optimisation of the catalytic procedure, PhOH was again chosen as a simple model substrate. To begin, analogous reactions to that described in the previous section were repeated using catalytic quantities of various disulfides (6.3 mol% per P atom) or equivalent loadings of the corresponding thiols (12.5 mol% per P atom).

Thus:

To a suspension of $Na_2S_2O_8$ (0.13 mmol, 31.0 mg, 3.25 eq. per P atom) and PhOH (0.13 mmol, 12.2 mg, 3.25 eq. per P atom) in MeCN (0.5 mL) was added Et_3N (0.13 mmol, 18.1 µL, 3.25 eq. per P atom), a stock solution of either diaryl disulfide (0.0025 mmol) or thiophenol (0.005 mmol) in MeCN, and P_4 (0.01 mmol, 93 µL, 0.108 M in C_6H_6), resulting in a colourless suspension which quickly became yellow and cloudy.

The resulting reaction mixtures were analysed by $^{31}P\{^1H\}$ NMR spectroscopy following addition of Ph_3PO as an internal standard (as a stock solution in MeCN) and in essentially all cases clear formation of the target product $(PhO)_3P$ could be observed, with conversions indicating appreciable catalytic turnover (Figure S52).

Using Hammett parameters as an indicator of electron-withdrawing ability,[75] the observed conversions can be plotted in the order of the thiols' expected electron deficiency (Figure S53). By doing so, it is possible to identify a clear trend. As the thiols become more electron deficient a steady increase in conversion is observed, peaking at 3-chlorothiophenol. Beyond this point, further increases lead to diminished performace, ultimately leading to no product formation in the extreme case of C_6F_5SH. We tentatively attribute these observations to the fact that thiols that are too electron rich will be poor leaving groups and hence difficult for the phenoxide nucleophile to displace, while those that are too electron deficient are likely to be harder (and hence slower) to subsequently re-oxidise to the corresponding disulfides.

A similar (though slightly less clear-cut) trend can be observed for the disulfides tested (Figure S54), and their performances are broadly similar to those of their corresponding thiols, as expected from the proposed reaction mechanism.

Overall, optimal results were obtained for Ar = 4-chlorophenyl (starting from Ar_2S_2) and so this motif was used as the focus for further reaction optimisation.

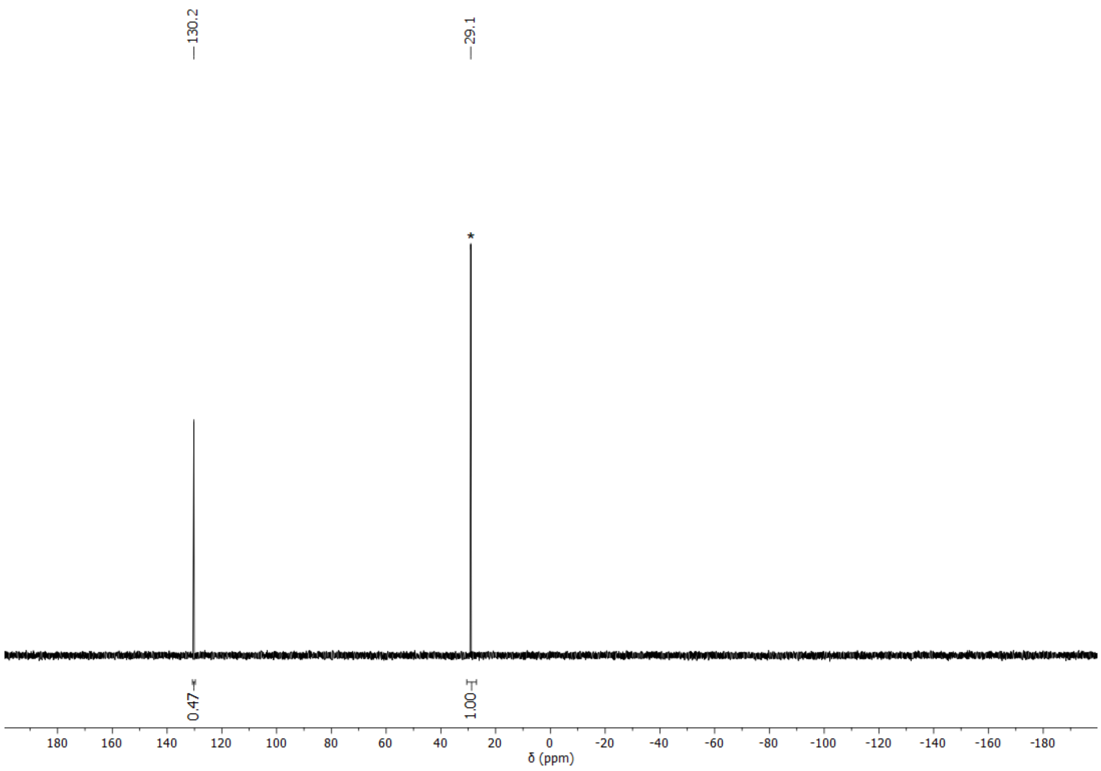

Figure S52. Representative quantitative single-scan $^{31}P\{^{1}H\}$ NMR spectrum for a reaction between P_4, PhOH (3.25 eq.), Et_3N (3.25 eq.) and $Na_2S_2O_8$ (3.25 eq.) in MeCN catalysed by ArSH (12.5 mol%). All eq. and mol% defined per P atom. * = Ph_3PO (internal standard, 0.04 mmol), In this case, Ar = 4-trifluoromethylphenyl.

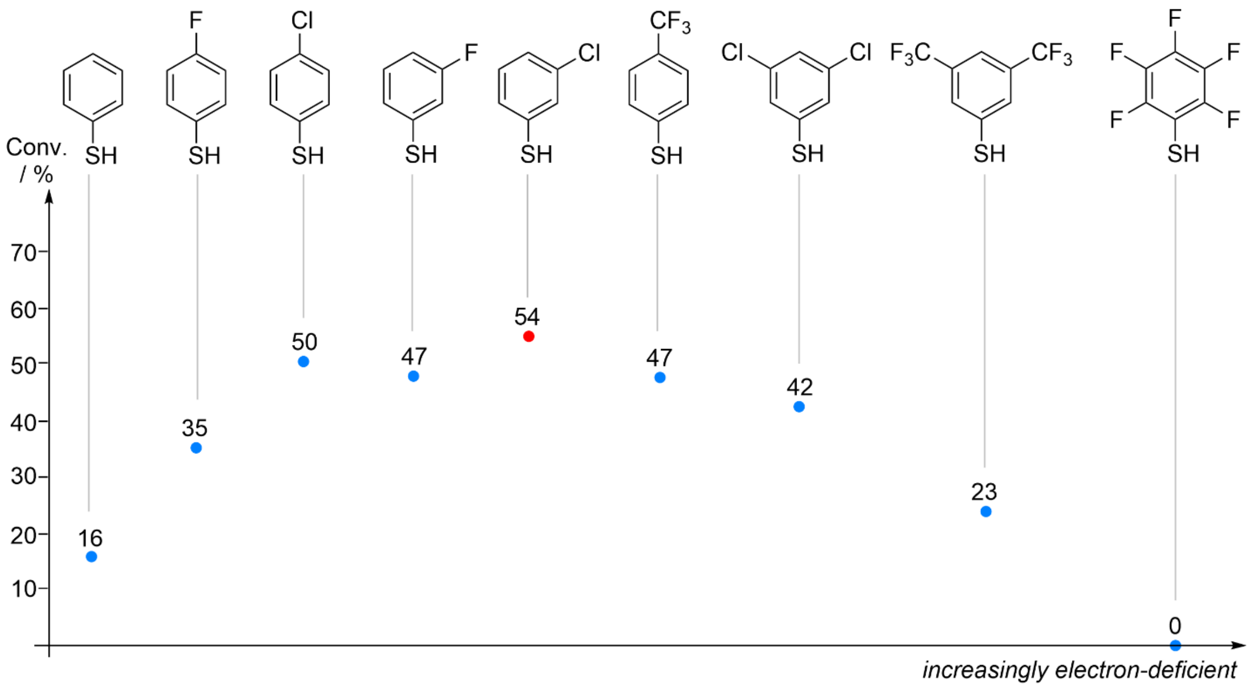

Figure S53. Conversion to $(PhO)_3P$ measured for reactions between P_4, PhOH (3.25 eq.), Et_3N (3.25 eq.) and $Na_2S_2O_8$ (3.25 eq.) in MeCN catalysed by ArSH (12.5 mol%). All eq. and mol% defined per P atom. Entries other than Ar = C_6F_5 are positioned in order of the relative, combined Hammett parameters of the substituents on Ar.

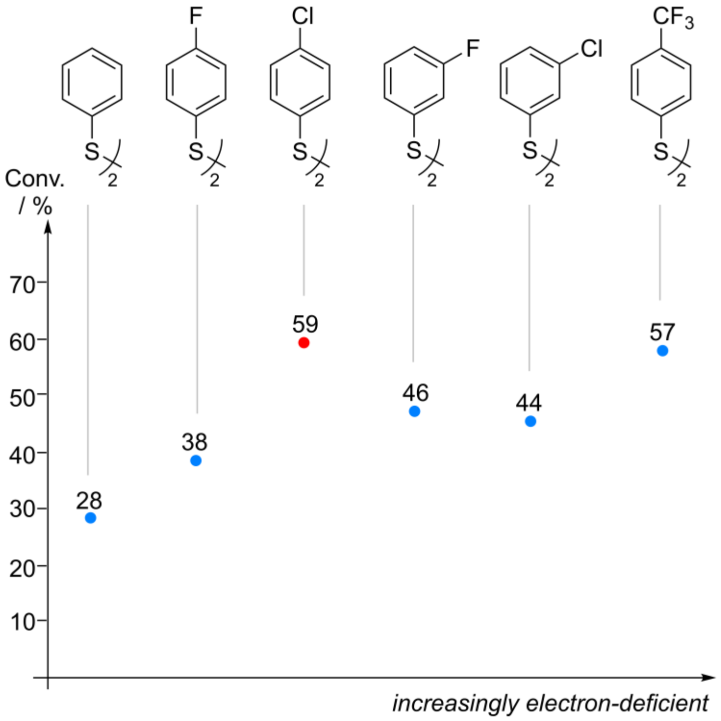

Figure S54. Conversion to $(PhO)_3P$ measured for reactions between P_4, PhOH (3.25 eq.), Et_3N (3.25 eq.) and $Na_2S_2O_8$ (3.25 eq.) in MeCN catalysed by Ar_2S_2 (6.3 mol%). All eq. and mol% defined per P atom. Entries are positioned in order of the relative, combined Hammett parameters of the substituents on Ar.

Optimisation of base

Following identification of appropriate catalysts, the effect of varying the base employed was also studied, using otherwise the same general procedure and 4-chlorothiophenol (^{Cl}ArSH) as catalyst.

Thus:

To a suspension of $Na_2S_2O_8$ (0.13 mmol, 31.0 mg, 3.25 eq. per P atom) and PhOH (0.13 mmol, 12.2 mg, 3.25 eq. per P atom) in MeCN (0.5 mL) was added the corresponding base (0.13 mmol, 3.25 eq. per P atom), ^{Cl}ArSH (0.005 mmol, 60.9 µL, 0.082 M in MeCN), and P_4 (0.01 mmol, 93 µL, 0.108 M in C_6H_6), resulting in a colourless suspension which quickly became yellow and cloudy. The resulting reaction mixtures were analysed by $^{31}P\{^1H\}$ NMR spectroscopy following addition of Ph_3PO as an internal standard (as a stock solution in MeCN).

From the results, a general dependence on pK_a could be observed (Figure S55), with good conversions observed for bases with pK_a values similar to that of Et_3N.[76] Weaker bases (DABCO, pyridine) gave

progressively poorer results, presumably due to an inability to efficiently deprotonate PhOH and/or ^{Cl}ArSH. Stronger bases similarly led to progressively poorer results, which we speculate may be due to unproductive, direct attack on $^{Cl}Ar_2S_2$.

Overall, optimal results were obtained using *i*Pr_2NEt and so this was used as the base for further reaction optimisation.

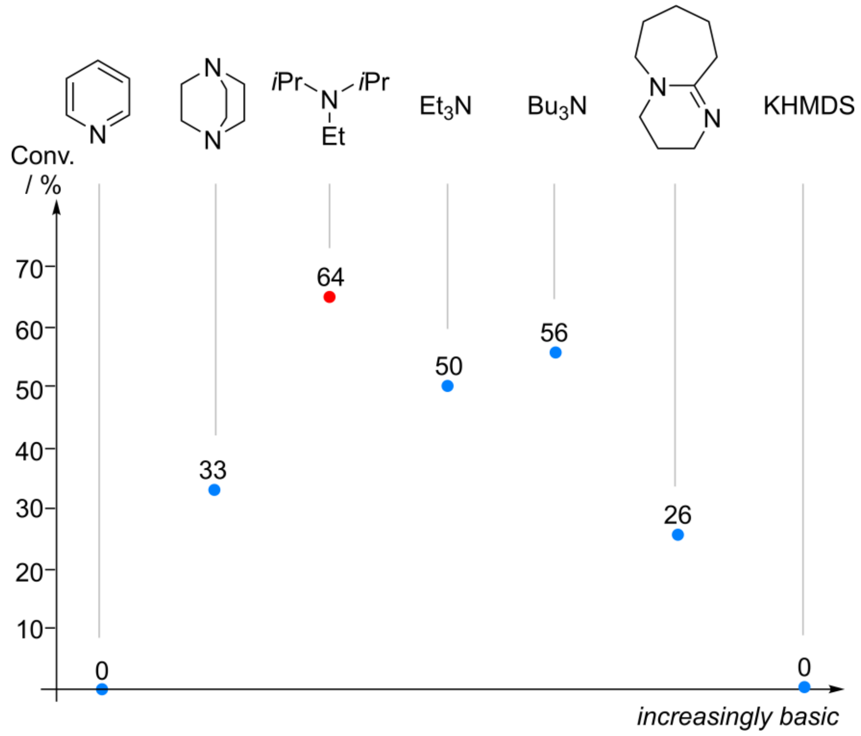

Figure S55. Conversion to $(PhO)_3P$ measured for reactions between P_4, PhOH (3.25 eq.), various bases (3.25 eq.) and $Na_2S_2O_8$ (3.25 eq.) in MeCN catalysed by ^{Cl}ArSH (12.5 mol%). All eq. and mol% defined per P atom. Entries are positioned in order of relative pK_a. ^{Cl}Ar = 4-chlorophenyl.

Optimisation of temperature and reaction time

Having identified an optimal catalyst and base, the effects of reduced reaction times and elevated temperatures were investigated (previous reactions all at room temperature for *ca*. 16 h).

Thus:

To a suspension of $Na_2S_2O_8$ (0.13 mmol, 31.0 mg, 3.25 eq. per P atom) and PhOH (0.13 mmol, 12.2 mg, 3.25 eq. per P atom) in MeCN (0.5 mL) was added $^{Cl}Ar_2S_2$ (0.0025 mmol, 26 μL, 0.095 M in MeCN) and *i*Pr_2NEt

(0.13 mmol, 22.7 μL, 3.25 eq. per P atom), followed by P_4 (0.01 mmol, 91 μL, 0.110 M in C_6H_6), resulting in a colourless suspension which quickly became yellow and cloudy. The reaction mixture was heated to the prescribed temperature in a sealed tube outside the glove box, then returned to the glove box after stirring for the defined amount of time. The resulting reaction mixtures were analysed by $^{31}P\{^1H\}$ NMR spectroscopy following addition of Ph_3PO as an internal standard (as a stock solution in MeCN).

From the results, the use of only gentle heating (40 °C) and modest reaction times (6 h) was found to be optimal (Table S5). Poorer outcomes observed using longer or higher temperature reactions are attributed to reduced selectivity and the formation of undesired side-products such as $(PhO)_3PO$.

Table S5. Conversion to $(PhO)_3P$ measured for reactions between P_4, PhOH (3.25 eq.), iPrNEt$_2$ (3.25 eq.) and $Na_2S_2O_8$ (3.25 eq.) in MeCN catalysed by $^{Cl}Ar_2S_2$ (6.3 mol%). All eq. And mol% defined per P atom. ^{Cl}Ar = 4-chlorophenyl.

Entry	*T* / °C	*t* / h	Conv. / %
1		3	50
**2**	**40**	**6**	**77**
3		8	71
4		2	56
5	50	4	56
6		6	55
7		0.6	21
8	80	4	20
9		6	21

Optimisation of peroxodisulfate salt and solvent

In the final stage of optimisation the peroxodisulfate salt and solvent were varied, as it was speculated that the low solubility of $Na_2S_2O_8$ in MeCN may limit reaction performance.

Thus:

To a suspension of peroxodisulfate (0.13 mmol, 3.25 eq. per P atom) and PhOH (0.13 mmol, 12.2 mg, 3.25 eq. per P atom) in the chosen solvent (0.5 mL) was added $^{Cl}Ar_2S_2$ (0.0025 mmol, 26 μL, 0.095 M in MeCN) and

iPr$_2$NEt (0.13 mmol, 22.7 μL, 3.25 eq.), followed by P_4 (0.01 mmol, 91 μL, 0.110 M in C_6H_6), resulting in a colourless suspension which quickly became yellow and cloudy. The reaction mixture was heated to 40 °C in a sealed tube outside the glove box, then returned to the glove box after stirring for 6 h. The resulting reaction mixtures were analysed by $^{31}P\{^1H\}$ NMR spectroscopy following addition of Ph_3PO as an internal standard (as a stock solution in MeCN).

In all cases examined the use of alternative salt/solvent combinations provided inferior results, and so $Na_2S_2O_8$/MeCN was confirmed as optimal (Table S6). It is worth noting that $Na_2S_2O_8$ is known to be capable of directly oxidizing tertiary amines,[77] and so we do not exclude the possibility that Et_3N could act as a redox shuttle/mediator for the insoluble terminal oxidant in this reaction, in addition to being an auxiliary base.

Table S6. Conversion to $(PhO)_3P$ measured for reactions between P_4, PhOH (3.25 eq.), *i*$PrNEt_2$ (3.25 eq.) and $M_2S_2O_8$ (3.25 eq.) in the indicated solvent catalysed by $^{Cl}Ar_2S_2$ (6.3 mol%) at 40 °C for 6 h. All eq. and mol% defined per P atom. ^{Cl}Ar = 4-chlorophenyl.

Entry	M	Solvent	Conv. / %
**1**		**MeCN**	**77**
2		DME	2
3	Na	DMSO	31
4		THF	2
5		1,4-Dioxane	3
6		MeCN	61
7		DME	2
8	K	DMSO	31
9		THF	3
10		1,4-Dioxane	3
11		MeCN	63
12		DME	2
13	NH_4	DMSO	40
14		THF	10
15		1,4-Dioxane	0
16	Bu_4N	MeCN	9

4.2. Catalytic synthesis of $(ArO)_3P$ from P_4 and ArOH

4.2.1. NMR scale procedure for *in situ* measurement of conversion

Representative procedure:

To a suspension of $Na_2S_2O_8$ (0.13 mmol, 31.0 mg, 3.25 eq. per P atom) and ArOH (0.13 mmol, 3.25 eq. per P atom) in MeCN (0.5 mL) was added $^{Cl}Ar_2S_2$ (0.0025 mmol, 26 µL, 0.095 M in MeCN) and *i*Pr_2NEt (0.13 mmol,

22.7 μL, 3.25 eq. per P atom), followed by P_4 (0.01 mmol, 91 μL, 0.110 M in C_6H_6), resulting in a colourless suspension which quickly became yellow and cloudy. The reaction mixture was heated to 40 °C in a sealed tube outside the glovebox, then returned to the glove box after stirring for 6 h. An internal standard of Ph_3PO (0.02 mmol, as a stock solution in MeCN) was added. The reaction was transferred to an NMR tube equipped with a C_6D_6 capillary and analysed by $^{31}P\{^1H\}$ NMR spectroscopy.

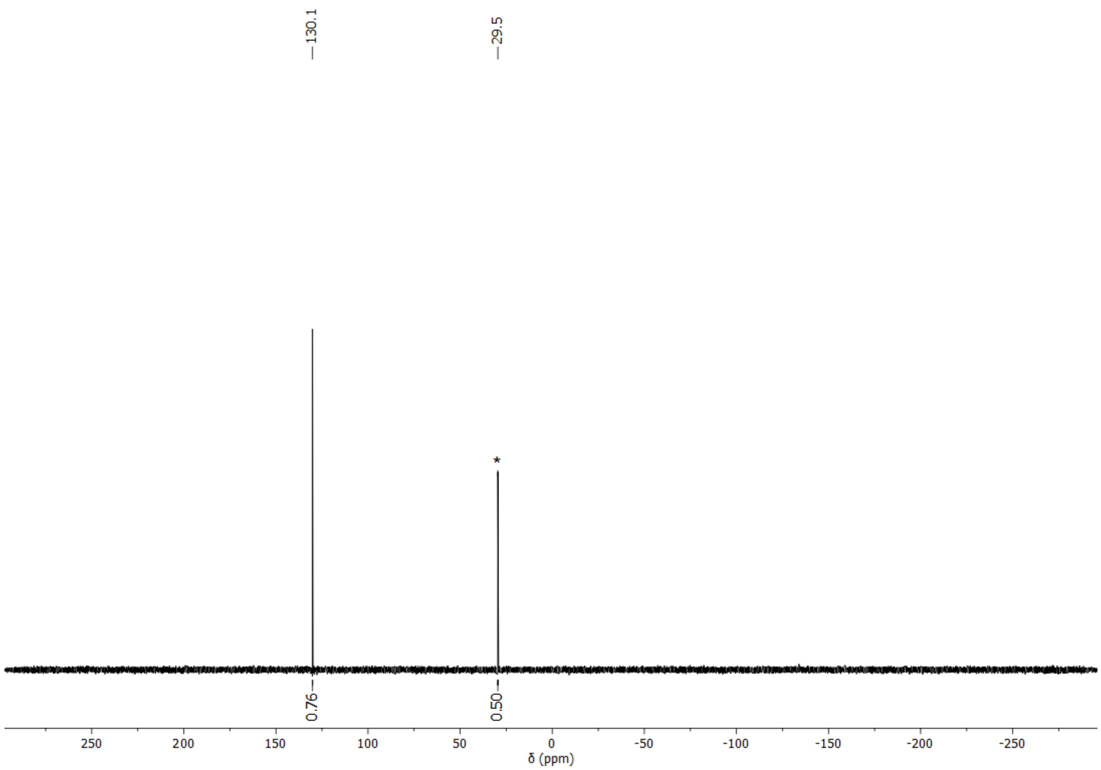

Figure S56. Quantitative single-scan $^{31}P\{^1H\}$ NMR spectrum of triphenylphosphite generated by the catalytic functionalisation of P_4 in MeCN. * = Ph_3PO (internal standard, 0.02 mmol)

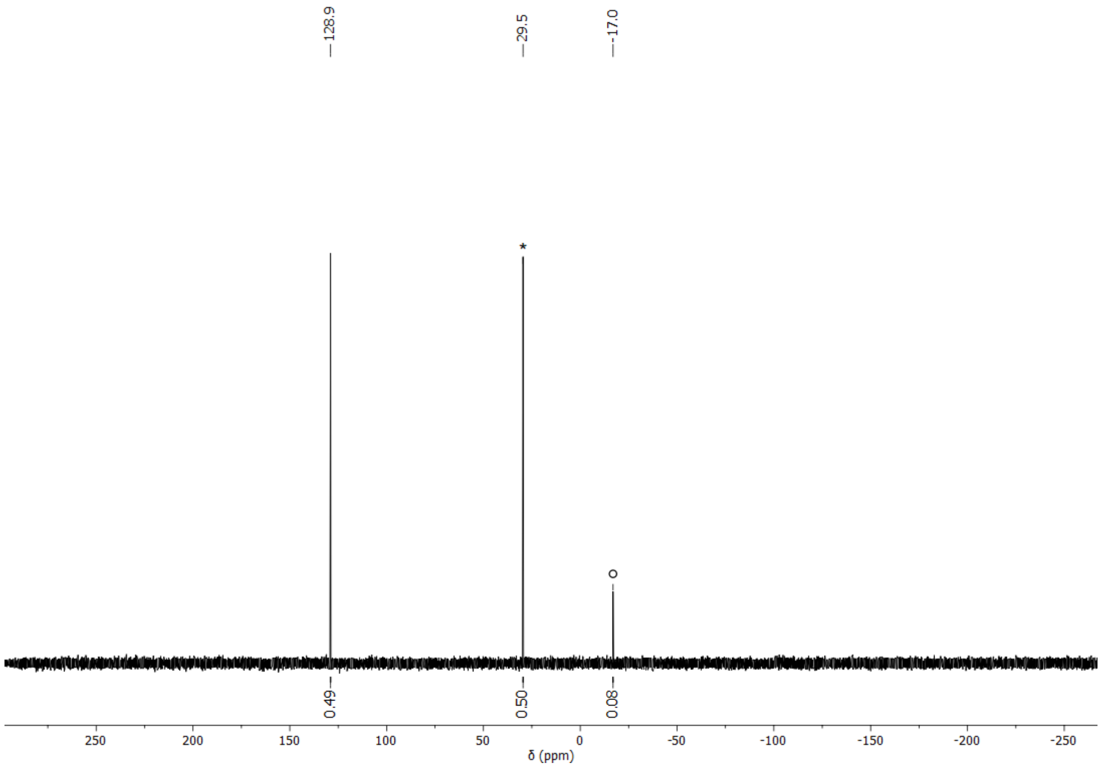

Figure S57. Quantitative single-scan $^{31}P\{^1H\}$ NMR spectrum of tris(3-fluorophenyl)phosphite generated by the catalytic functionalisation of P_4 in MeCN. * = Ph_3PO (internal standard, 0.02 mmol), ○ = tris(3-fluorophenyl)phosphate.

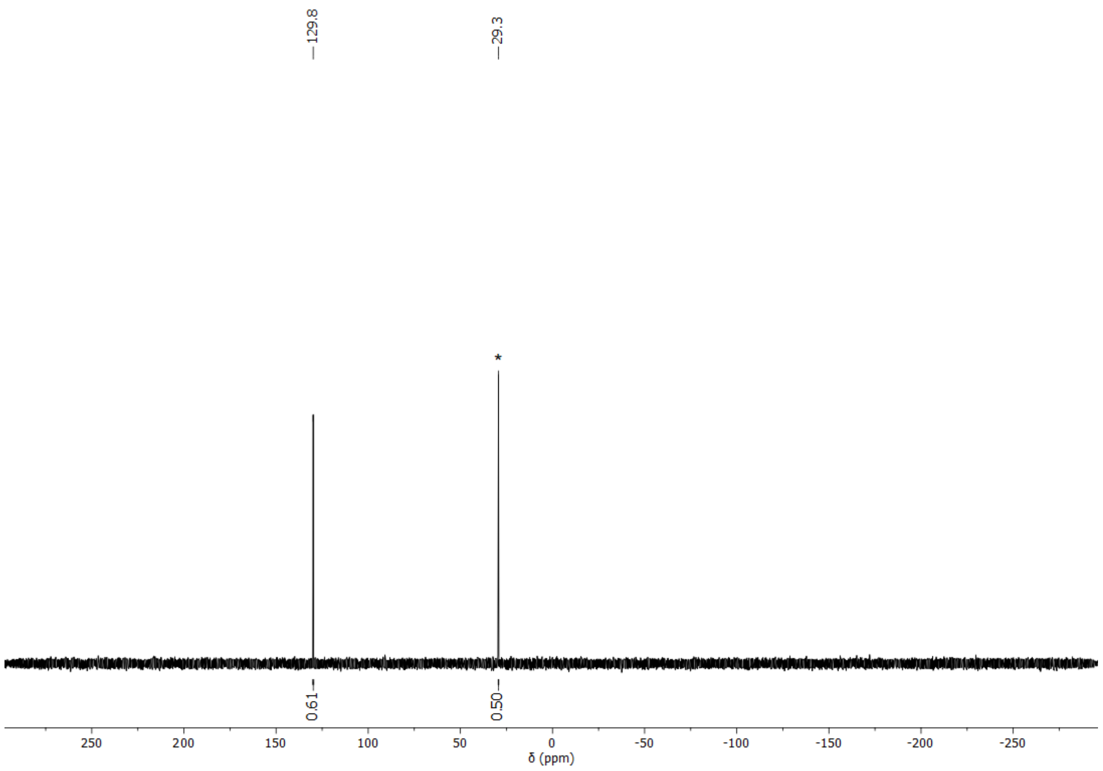

Figure S58. Quantitative single-scan $^{31}P\{^{1}H\}$ NMR spectrum of tris(4-fluorophenyl)phosphite generated by the catalytic functionalisation of P_4 in MeCN. * = Ph_3PO (internal standard, 0.02 mmol)

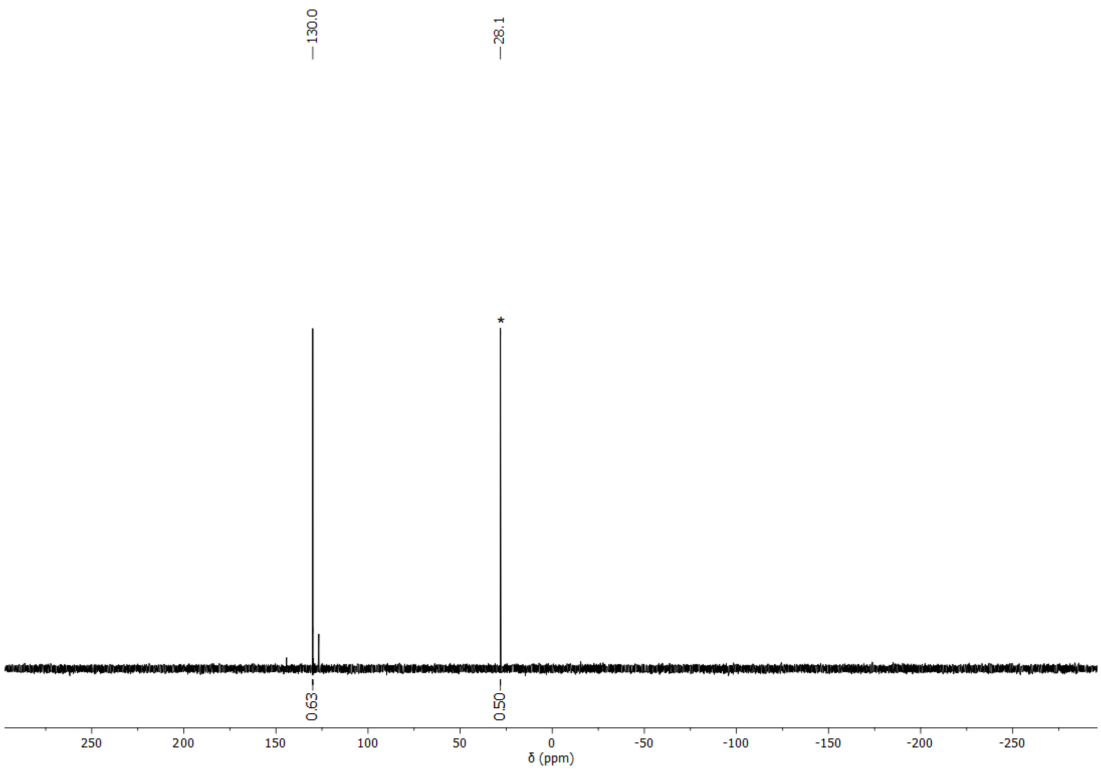

Figure S59. Quantitative single-scan $^{31}P\{^{1}H\}$ NMR spectrum of tris(4-methylmercaptophenyl)phosphite generated by the catalytic functionalisation of P_4 in MeCN. * = Ph_3PO (internal standard, 0.02 mmol)

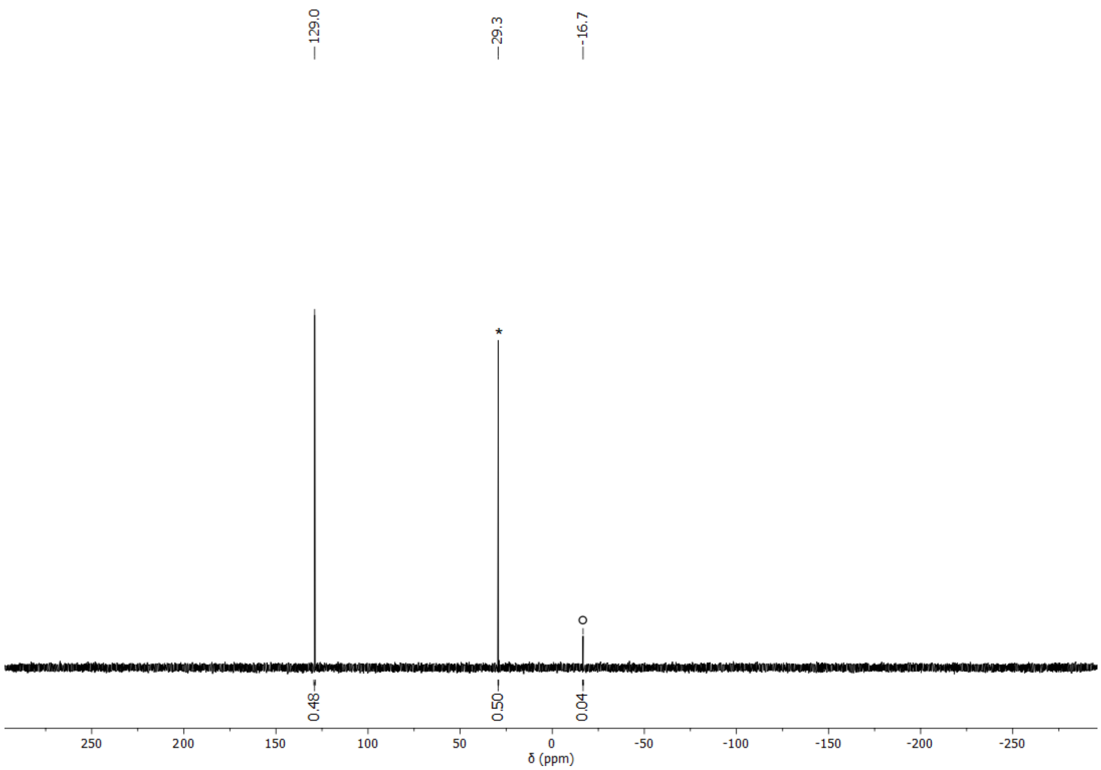

Figure S60. Quantitative single-scan $^{31}P\{^1H\}$ NMR spectrum of tris(3-chlorophenyl)phosphite generated by the catalytic functionalisation of P_4 in MeCN. * = Ph_3PO (internal standard, 0.02 mmol), ○ = tris(3-chlorophenyl)phosphate

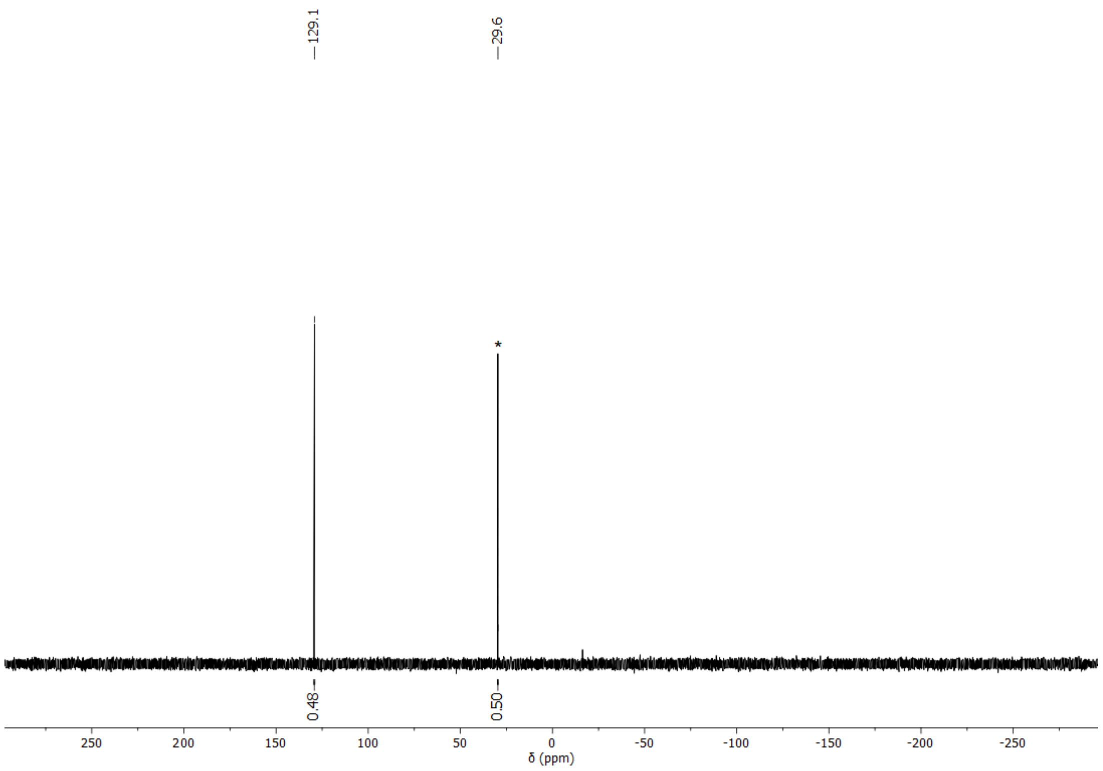

Figure S61. Quantitative single-scan $^{31}P\{^{1}H\}$ NMR spectrum of tris(4-chlorophenyl)phosphite generated by the catalytic functionalisation of P_4 in MeCN. * = Ph_3PO (internal standard, 0.02 mmol)

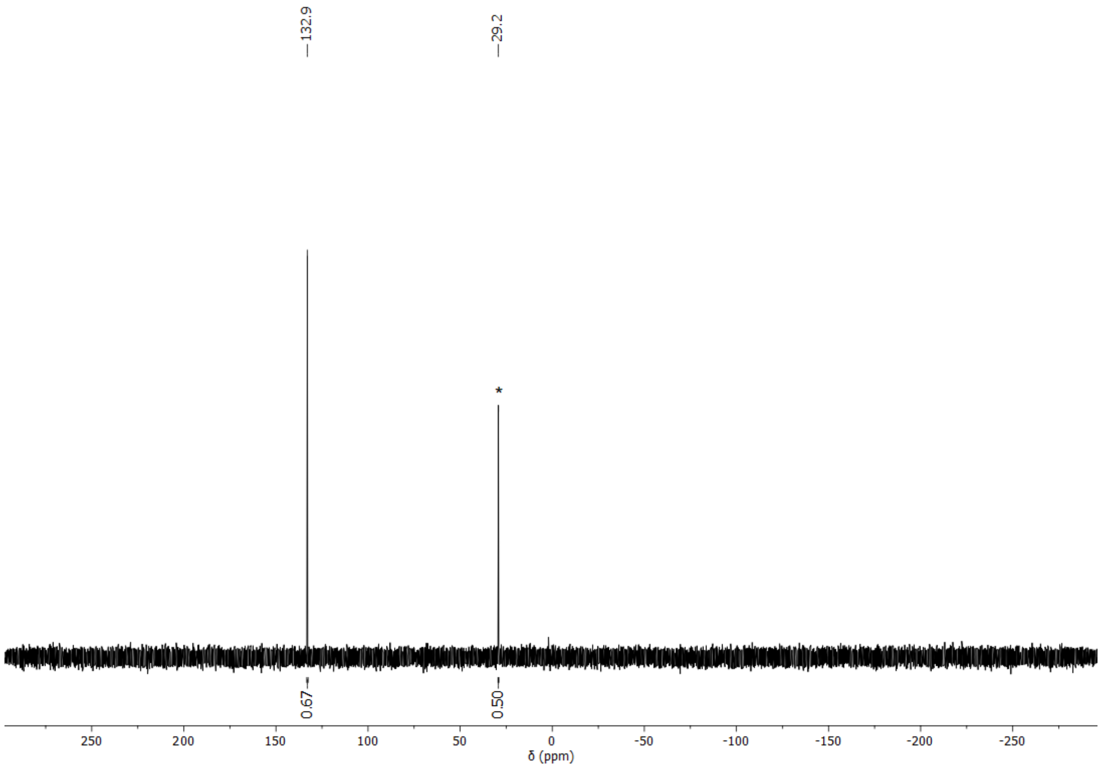

Figure S62. Quantitative single-scan $^{31}P\{^{1}H\}$ NMR spectrum of tris(2-methylphenyl)phosphite generated by the catalytic functionalisation of P_4 in MeCN. * = Ph_3PO (internal standard, 0.02 mmol)

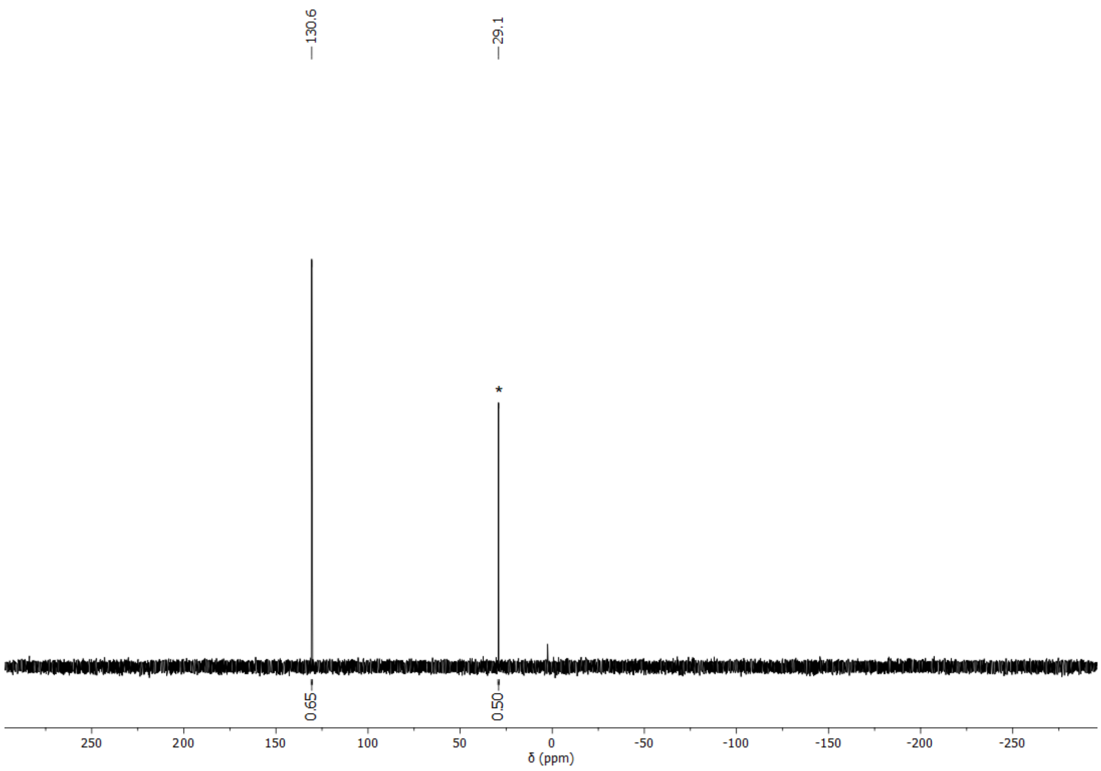

Figure S63. Quantitative single-scan $^{31}P\{^{1}H\}$ NMR spectrum of tris(3-methylphenyl)phosphite generated by the catalytic functionalisation of P_4 in MeCN. * = Ph_3PO (internal standard, 0.02 mmol)

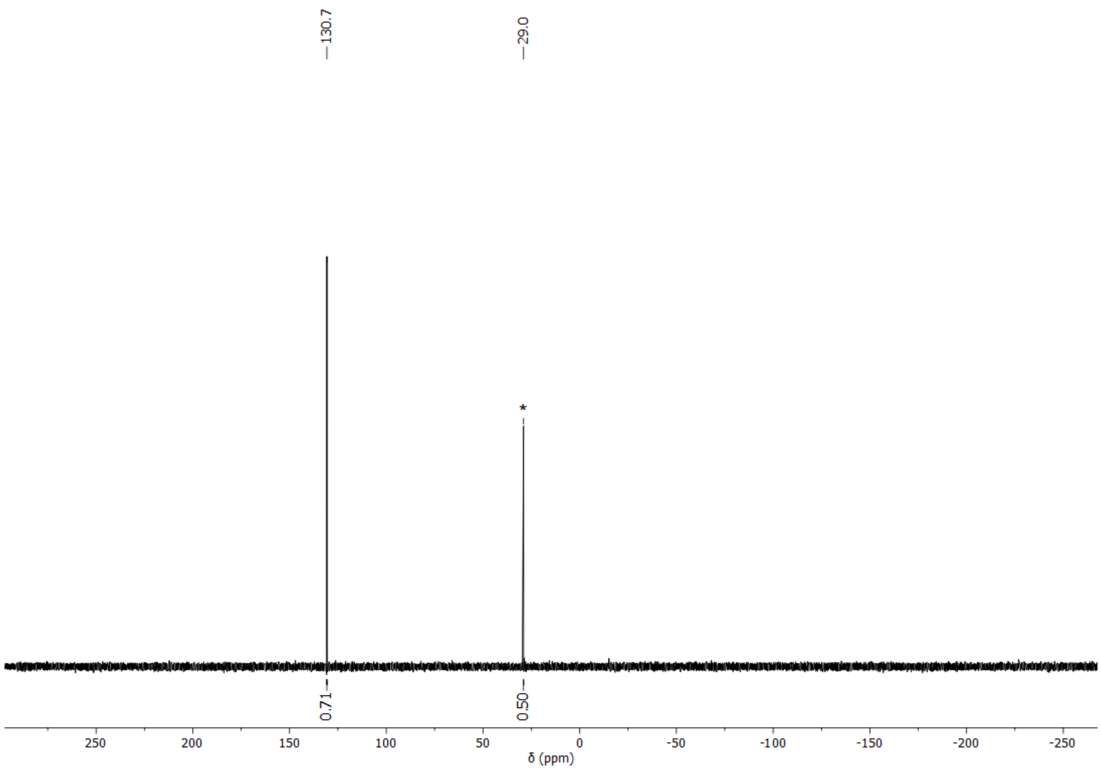

Figure S64. Quantitative single-scan $^{31}P\{^1H\}$ NMR spectrum of tris(4-methylphenyl)phosphite generated by the catalytic functionalisation of P_4 in MeCN. * = Ph_3PO (internal standard, 0.02 mmol)

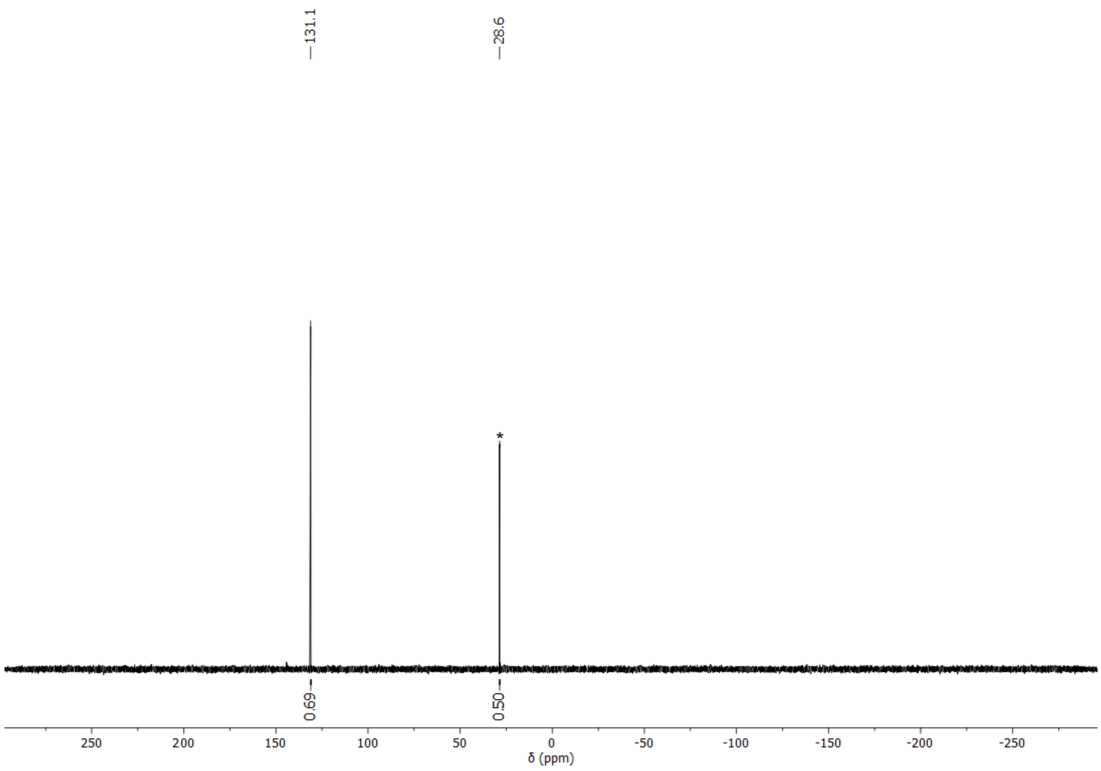

Figure S65. Quantitative single-scan $^{31}P\{^{1}H\}$ NMR spectrum of tris(4-methoxyphenyl)phosphite generated by the catalytic functionalisation of P_4 in MeCN. * = Ph_3PO (internal standard, 0.02 mmol)

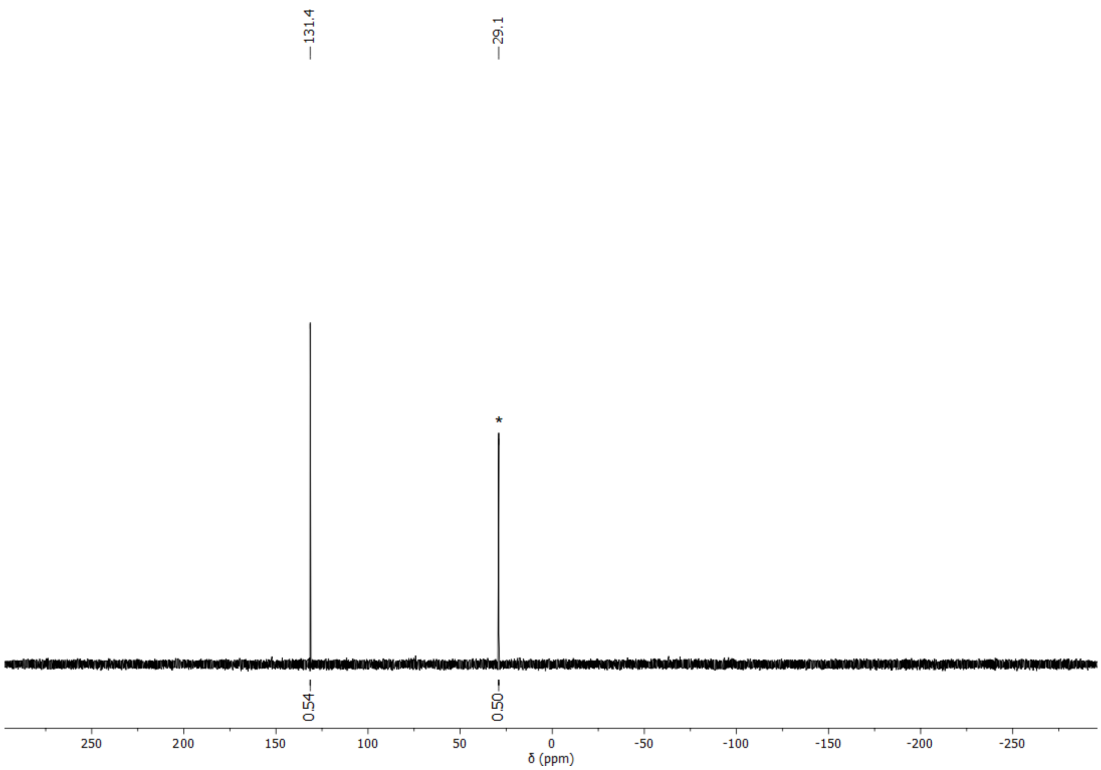

Figure S66. Quantitative single-scan $^{31}P\{^{1}H\}$ NMR spectrum of tris(3,5-dimethoxyphenyl)phosphite generated by the catalytic functionalisation of P_4 in MeCN. * = Ph_3PO (internal standard, 0.02 mmol)

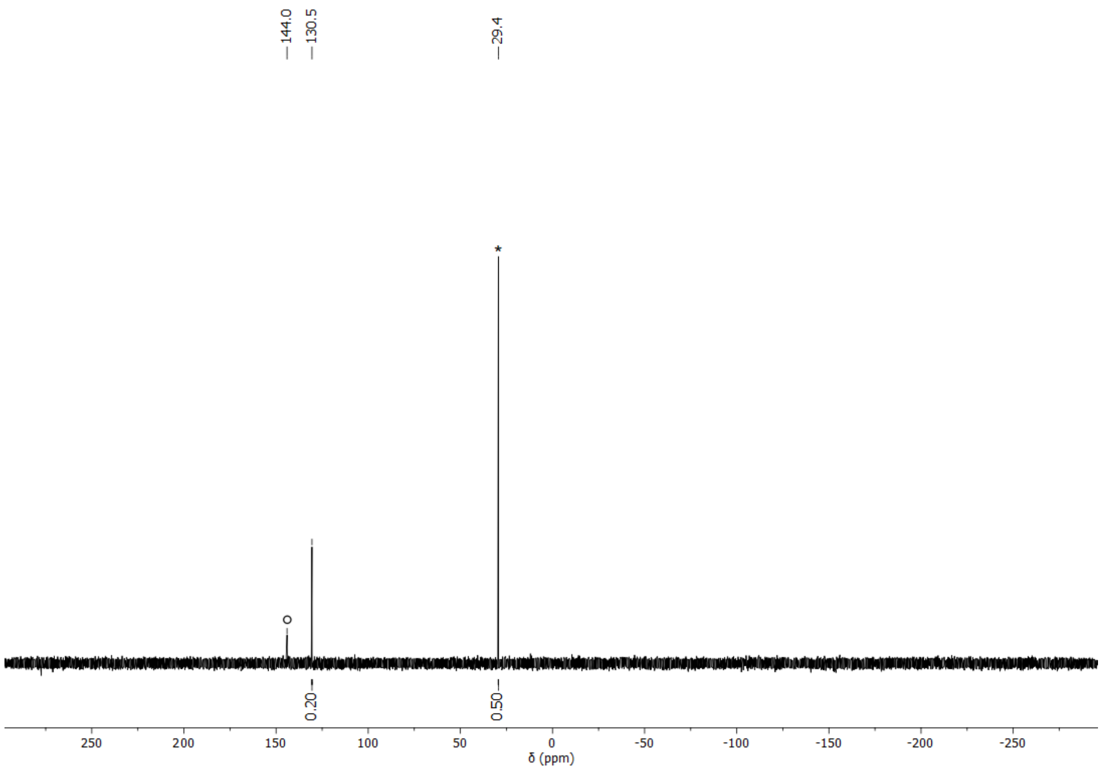

Figure S67. Quantitative single-scan $^{31}P\{^{1}H\}$ NMR spectrum of tris(4-acetamidophenyl)phosphite generated by the catalytic functionalisation of P_4 in MeCN. * = Ph_3PO (internal standard, 0.02 mmol), ○ = unidentified side product

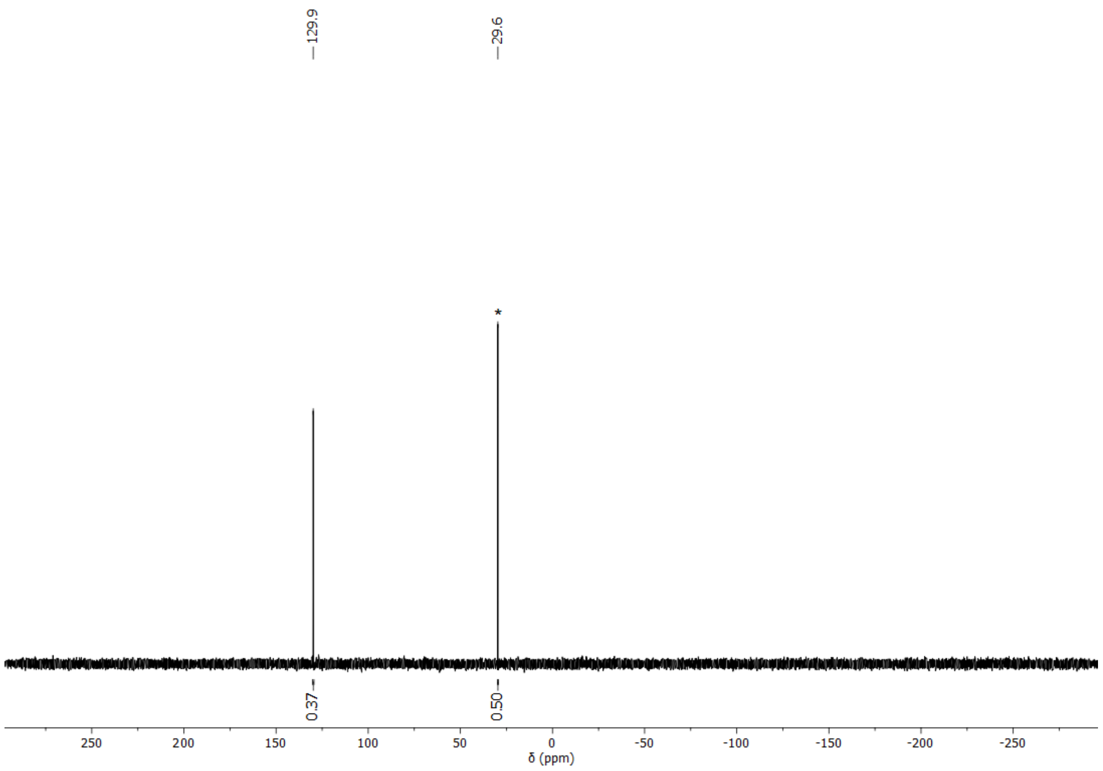

Figure S68. Quantitative single-scan $^{31}P\{^{1}H\}$ NMR spectrum of tris(2-naphthyl)phosphite generated by the catalytic functionalisation of P_4 in MeCN. * = Ph_3PO (internal standard, 0.02 mmol)

4.2.2. Larger scale catalytic synthesis of $(PhO)_3P$

To a suspension of $Na_2S_2O_8$ (5.2 mmol, 1.24 g, 3.25 eq. per P atom), PhOH (5.2 mmol, 0.49 g, 3.25 eq. per P atom) and $^{Cl}Ar_2S_2$ (0.1 mmol, 29 mg) in MeCN (17 mL) was added *i*Pr_2NEt (5.2 mmol, 906 µL, 3.25 eq. per P atom), followed by a solution of P_4 (0.4 mmol, 50 mg) in benzene (2.5 mL), resulting in a colourless suspension which quickly became yellow and cloudy. The reaction mixture was heated to 40 °C in a sealed ampoule outside the glovebox, then returned to the glovebox after stirring at this temperature for 6 h.

The resulting suspension was filtered to remove the yellow solids and the solution was concentrated *in vacuo* at room temperature until the formation of a precipitate was observed (to a total volume of approx. 1 mL). Hexane (approx. 20 mL) was added and the two phases were thoroughly stirred for *ca*. 30 min. The resulting mixture was concentrated *in vacuo* to half of its volume, forming a brown oil under a yellow hexane solution. The mixture was left to stand overnight and the hexane phase was collected *via* cannula. The volatiles were removed *in vacuo* at room temperature and then dried at 120 °C. Triphenyl phosphite (393 mg, 1.27 mmol, 79%) was isolated as a viscous, pale-yellow oil by distillation at 160 °C *in vacuo* (*ca.* 5 x 10^{-2} mbar). NMR data are identical to those provided in section 3.4.1.

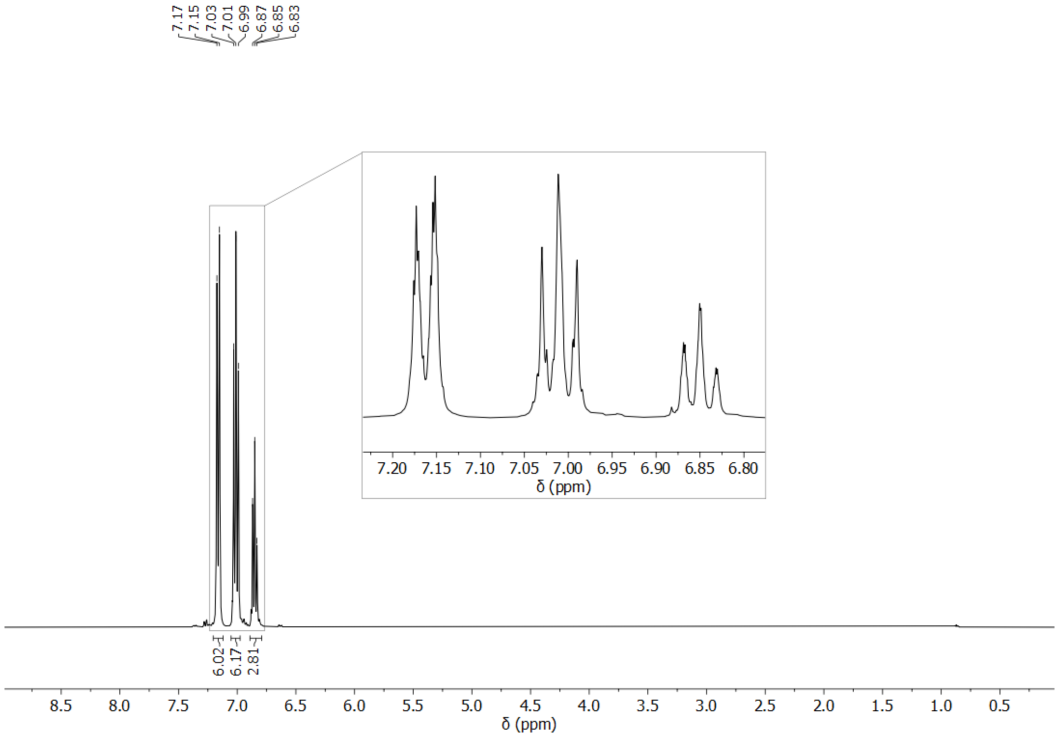

Figure S69. ^1H NMR spectrum of catalytically synthesised triphenylphosphite in C_6D_6.

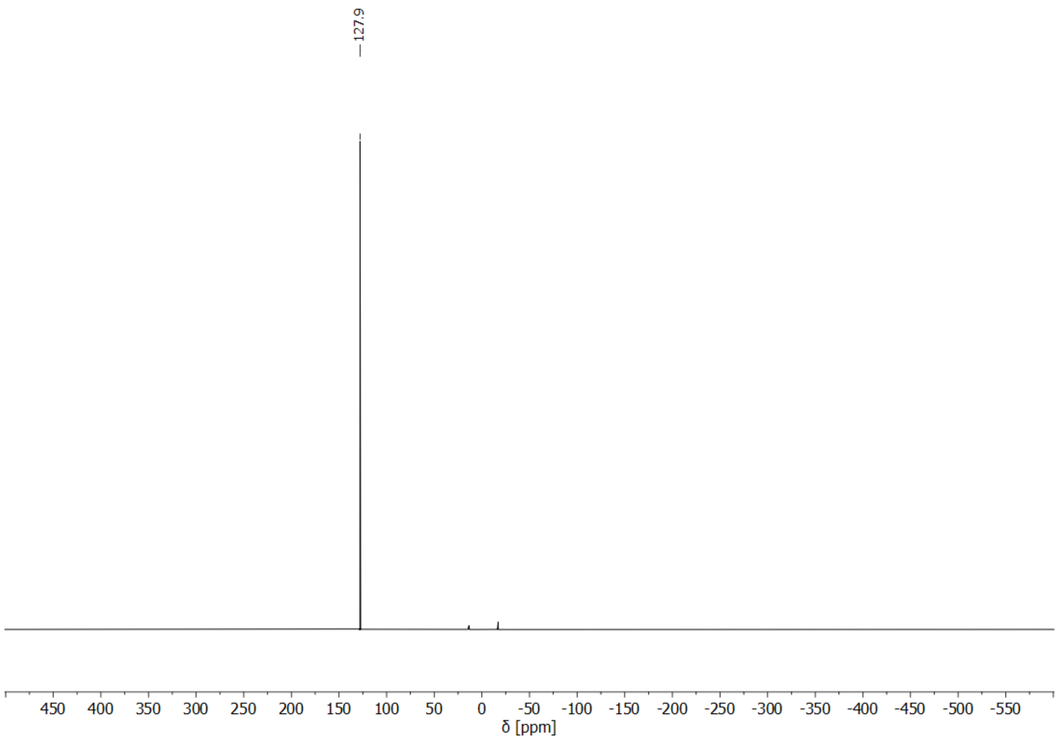

Figure S70. $^{31}P\{^{1}H\}$ NMR spectrum of catalytically synthesised triphenylphosphite in C_6D_6.

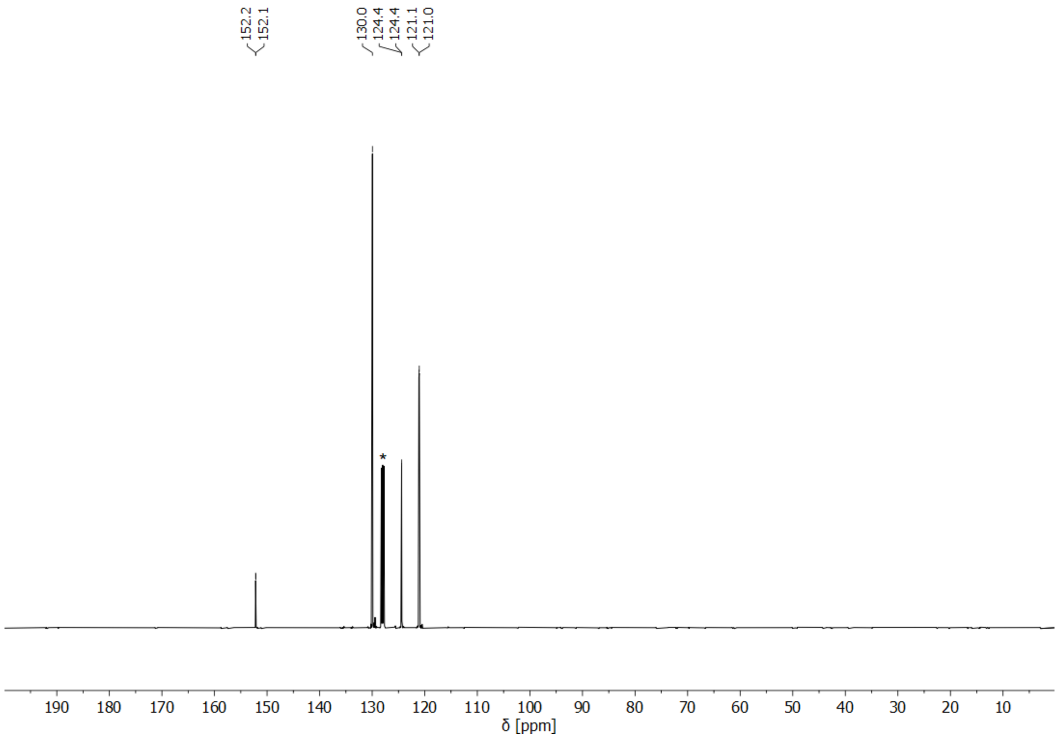

Figure S71. $^{13}C\{^{1}H\}$ NMR spectrum of catalytically synthesised triphenylphosphite in C_6D_6 (*).

5. Electrocatalytic reaction development

5.1. Proof-of-concept experiment

After optimisation of the catalytic system using $Na_2S_2O_8$ as a terminal oxidant, the next goal was to develop a more atom economical electrochemical system in which the stoichiometric oxidant could be replaced by the direct anodic oxidation of the thiolate unit (ArS^-) to the corresponding diaryl disulfide (Ar_2S_2), with hydrogen evolution (HER) as the cathodic counter reaction. To facilitate HER, the concentration of protons was increased by adding additional excess of PhOH (9 eq. per P atom) and the model reaction was scaled up to a total volume of 6 mL (with 0.08 mmol of P_4) to have a greater immersed area of the electrodes. Platinum foil was selected as cathode due to its known low overpotential for HER in tetrabutylammonium electrolytes, and reticulated vitreous carbon (RVC) was chosen as the working electrode due to its greater surface area, which was thought could lead to lower working potentials in a galvanostatic regime and prevent possible P_4 polymerisation side reactions.

5.1.1 Electocatalytic generation of $(PhO)_3P$ and *in situ* measurement of conversion

A 10 mL undivided cell with a stir bar was charged with $^nBu_4NPF_6$ (0.6 mmol, 232.5 mg, 0.1 M), phenol (2.88 mmol, 271.1 mg, 9 eq. per P atom) and a stock solution of bis(4-chlorophenyl)disulfide (0.02 mmol, 800 µL, 0.025 M in MeCN, 6.25 mol% per P atom). To the resulting colourless solution was added *i*Pr_2NEt (1.04 mmol, 181 µL, 3.25 eq. per P atom), followed in order by a stock solution of P_4 (0.08 mmol, 500 µL, 0.16 M in C_6H_6) and MeCN (4519 µL) to reach a total volume of 6 mL. The RVC anode (8 x 27 mm) was pierced with the sharp tip of a graphite pencil rod (Figure S72a-b) and attached to the cell cap along with the platinum foil cathode (Figure S67c-d). The capped electrochemical cell was connected to an *IKA ElectraSyn 2.0* device and a galvanostatic regime of 1.5 mA was set up in the system (Figure S73). The reaction was stirred (300 rpm) at ambient temperature and run until a total charge of 12.3 F/mol of P_4 was consumed. After completion of the electrolysis, a clear colourless supernatant and a grey-brown precipitate were observed.

The resulting reaction mixture was analysed by $^{31}P\{^1H\}$ NMR spectroscopy following addition of Ph_3PO as an internal standard (0.08 mmol, 500 µL, 0.16 M in MeCN) and filtration of the solids. The formation of the target product $(PhO)_3P$ was observed in a 62% yield (Figure S74), corresponding to a TON of 15.

$^{31}P\{^1H\}$ NMR (162 MHz, C_6D_6) δ 130.2 ppm (s).

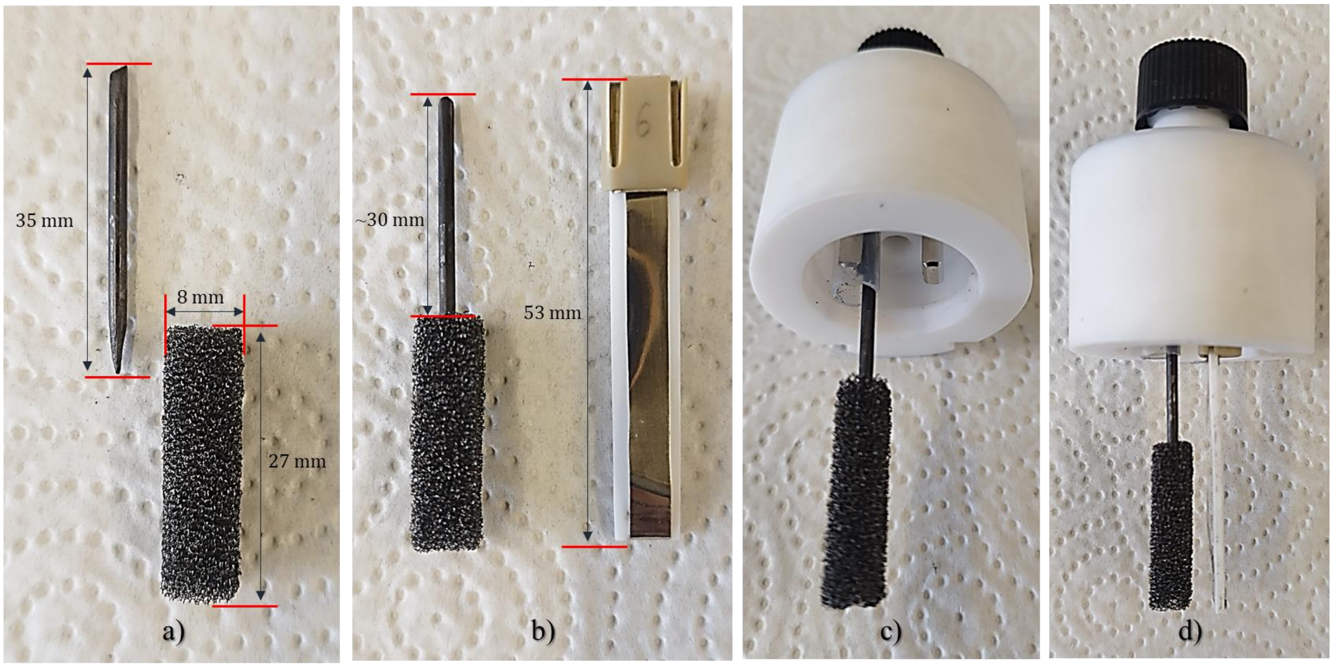

Figure S72. a) Sharp graphite pencil rod (35 mm x 2 mm diameter) used to attach the piece of RVC material (8 x 27 mm) employed as working electrode (anode). b) RVC electrode pierced with the sharp tip (~5 mm) of the pencil rod and IKA platinum foil electrode used as cathode. c) RVC electrode attached to the cell cap with a plastic connection. d) Electrochemical cell cap with both electrodes connected.

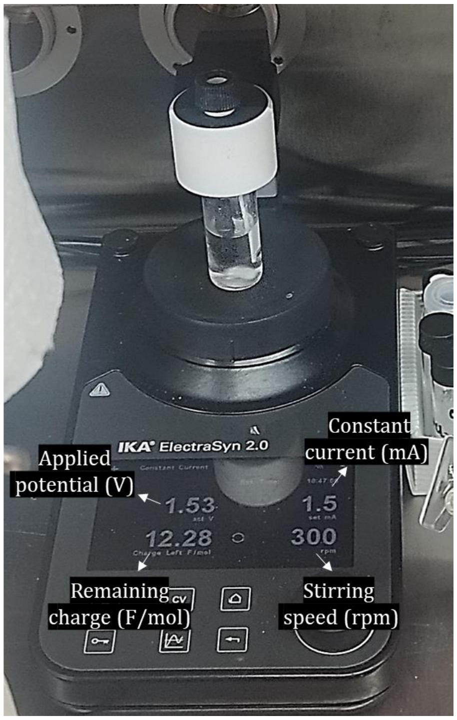

Figure S73. Electrochemical cell with the initial reaction solution connected to the *IKA ElectraSyn 2.0* device inside the glovebox. The initial electrochemical parameters can be observed: 1.5 mA constant current, 12.28 F/mol-P_4 remaining charge (after consumption of 0.02 F/mol from the initial 12.30), 1.53 V variable applied potential and 300 rpm stirring.

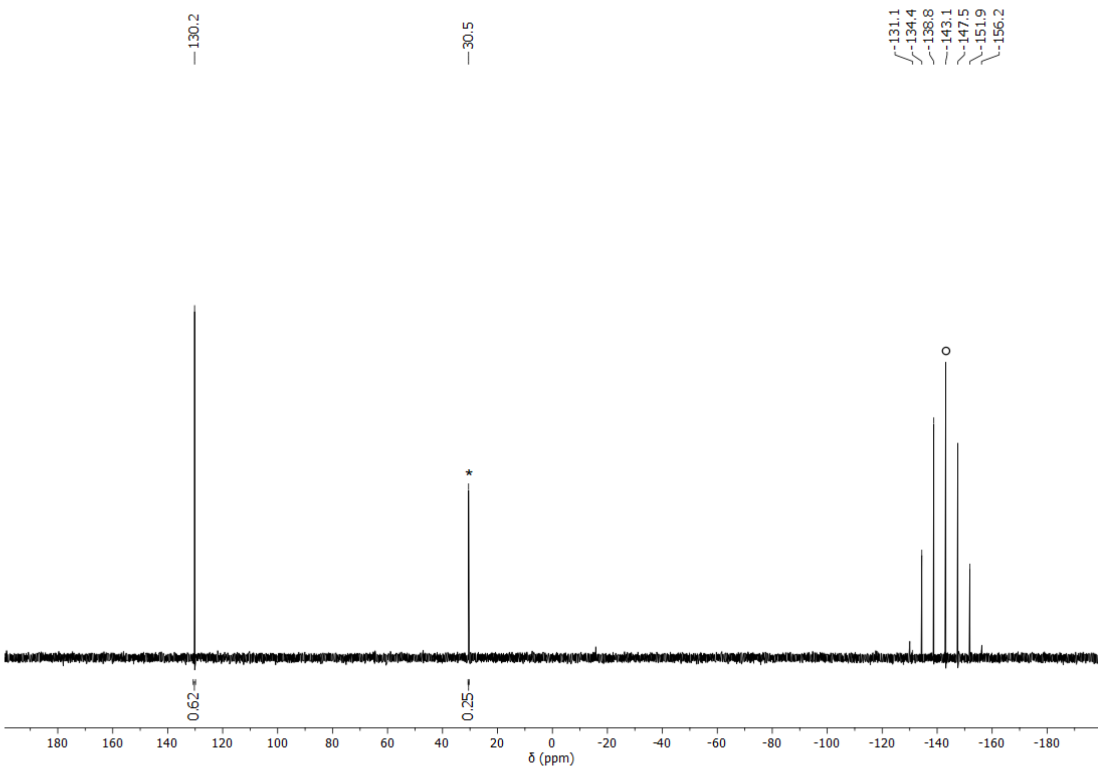

Figure S74. Quantitative single-scan $^{31}P\{^{1}H\}$ NMR spectrum of triphenylphosphite generated by the electrocatalytic functionalisation of P_4 in MeCN (proof-of-concept experiment). * = Ph_3PO (internal standard, 0.08 mmol). °= PF_6 anion (supporting electrolyte).

6. XRD data for $(Ph_2N)_2PS^{Cl}Ar$

Single crystals of $(Ph_2N)_2PS^{Cl}Ar$ suitable for X-ray diffraction were grown from a saturated *n*-hexane solution stored at −30 °C, as described in section 3.3.3.

The single-crystal X-ray diffraction data were recorded on an XtaLAB Synergy R DW system HyPix-Arc 150 diffractometer with Cu-Kα radiation (λ = 1.54184 Å). Crystals were selected under mineral oil, mounted on micro mount loops and quench-cooled using an open flow N_2 cooling device. Semi-empirical multi-scan[78] and analytical[79] absorption corrections were applied to the data.

The structures were solved with the SHELXT[80] solution program using using Olex2[81] as the graphical interface. The models were refined with ShelXL[82] using full matrix least squares minimization on F2.[83] The hydrogen atoms were located in idealized positions and refined isotropically with a riding model. The structure and relevant data are shown in Figure S75 and Table S7, below.

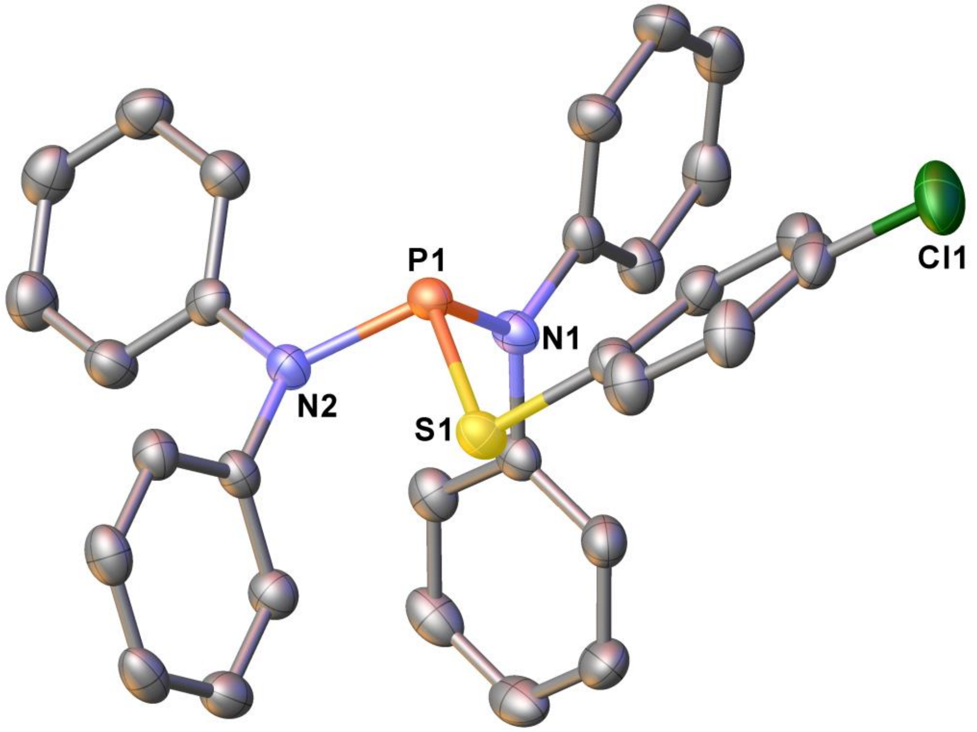

Figure S75. Solid-state molecular structure of $(4\text{-}ClC_6H_4)SP(NPh_2)_2$. Thermal ellipsoids are drawn at 50 % probability level. H atoms are omitted for clarity.

Table S7. Crystallographic data and structure refinement for $(Ph_2N)_2P(SC_6H_4Cl)$.

Formula	$C_{30}H_{24}N_2PSCl$
Formula weight	510.99
Temperature/K	123.01(10)
Crystal system	monoclinic
Space group	$P2_1/n$
a/Å	9.68420(10)
b/Å	22.9797(4)
c/Å	12.2703(2)
α/°	90
β/°	108.993(2)
γ/°	90
Volume/Å^3	2581.97(7)
Z	4
ρ_{calc}g/cm^3	1.315
μ/mm^{-1}	2.812
F(000)	1064.0
Crystal size/mm^3	0.079 × 0.037 × 0.027
Radiation	Cu Kα (λ = 1.54184)
2Θ range for data collection/°	7.694 to 146.262
Index ranges	-11 ≤ h ≤ 8, -28 ≤ k ≤ 27, -14 ≤ l ≤ 15
Reflections collected	28792
Independent reflections	5104 [R_{int} = 0.0341, R_{sigma} = 0.0288]
Data/restraints/parameters	5104/0/316
Goodness-of-fit on F^2	1.042
Final R indexes [I>=2σ (I)]	R_1 = 0.0360, wR_2 = 0.0953
Final R indexes [all data]	R_1 = 0.0443, wR_2 = 0.0995
Largest diff. peak/hole / e Å^{-3}	0.49/-0.28

7. References for supporting information

For refs. 1-69, refer to the main manuscript.

[70] I.-S. Shin, S. Yoon, J. I. Kim, J.-K. Lee, T. H. Kim, H. Kim, *Electrochim. Acta* **2011**, *56*, 6219-6223.

[71] A. Postigo, S. Barata, A. Ogawa, M. Sonoda, "Tetraphenyldiphosphine" in *Encyclopedia of reagents for organic synthesis*, Wiley, Weinheim **2008**.

[72] A. D. Burrows, M. F. Mahon, M. Varrone, *Dalton Trans.* **2004**, 3321-3330.

[73] G. Tang, T. Ji, A.-F. Hu, Y.-F. Zhao, *Synlett* **2008**, 1907-1909.

[74] J. Babin, *Z. Anorg. Allg. Chem.* **1980**, *467*, 218-224.

[75] L. P. Hammett, *J. Am. Chem. Soc.* **1937**, *59*, 96-103.

[76] F. G. Bordwell, *Acc. Chem. Res.* **1988**, *21*, 456-463.

[77] E. M. Alvarez, T. Karl, F. Berger, L. Torkowski, T. Ritter, *Angew. Chem. Int. Ed.* **2021**, *60*, 13609-13613.

[78] a) Sheldrick, G. M. SADABS, Bruker AXS, Madison, USA 2007; b) CrysAlisPro, Scale3 Abspack, Rigaku Oxford Diffraction 2019.

[79] R. C. Clark, J. S. Reid, *Acta Cryst. A* **1995**, *51*, 887–897.

[80] G. M. Sheldrick, *Acta Cryst. A* **2015**, *71*, 3–8.

[81] O. V. Dolomanov, L. J. Bourhis, R. J. Gildea, J. A. K. Howard, H. Puschmann, *Appl. Crystallogr.* **2009**, *42*, 339–341.

[82] G. M. Sheldrick, *Acta Cryst. C* **2015**, *71*, 3–8.

[83] G. M. Sheldrick, *Acta Cryst. A* **2008**, *64*, 112–122.
